# Prevalence of polypharmacy in pregnancy: a systematic review

**DOI:** 10.1136/bmjopen-2022-067585

**Published:** 2023-03-06

**Authors:** Astha Anand, Katherine Phillips, Anuradhaa Subramanian, Siang Ing Lee, Zhaonan Wang, Rebecca McCowan, Utkarsh Agrawal, Adeniyi Frances Fagbamigbe, Catherine Nelson-Piercy, Peter Brocklehurst, Christine Damase-Michel, Maria Loane, Krishnarajah Nirantharakumar, Amaya Azcoaga-Lorenzo, the MuM-PreDiCT Group

**Affiliations:** 1 Institute of Applied Health Research, University of Birmingham, Birmingham, UK; 2 University of Glasgow, Glasgow, UK; 3 Division of Population and Behavioural Sciences, University of Saint Andrews School of Medicine, St. Andrews, UK; 4 Epidemiology and Medical Statistics, University of Ibadan, Ibadan, Nigeria; 5 Institute of Applied Health Sciences, University of Aberdeen, Aberdeen, UK; 6 Guy's and St Thomas' NHS Foundation Trust, London, UK; 7 CHU Toulouse, Université Toulouse III, CHU Toulouse, Toulouse, France; 8 Institute of Nursing and Health Research, University of Ulster, Belfast, UK

**Keywords:** OBSTETRICS, Maternal medicine, EPIDEMIOLOGY

## Abstract

**Objectives:**

The use of medications among pregnant women has been rising over the past few decades but the reporting of polypharmacy has been sporadic. The objective of this review is to identify literature reporting the prevalence of polypharmacy among pregnant women, the prevalence of multimorbidity in women taking multiple medications in pregnancy and associated effects on maternal and offspring outcomes.

**Design:**

MEDLINE and Embase were searched from their inception to 14 September 2021 for interventional trials, observational studies and systematic reviews reporting on the prevalence of polypharmacy or the use of multiple medications in pregnancy were included.

Data on prevalence of polypharmacy, prevalence of multimorbidity, combinations of medications and pregnancy and offspring outcomes were extracted. A descriptive analysis was performed.

**Results:**

Fourteen studies met the review criteria. The prevalence of women being prescribed two or more medications during pregnancy ranged from 4.9% (4.3%–5.5%) to 62.4% (61.3%–63.5%), with a median of 22.5%. For the first trimester, prevalence ranged from 4.9% (4.7%–5.14%) to 33.7% (32.2%–35.1%). No study reported on the prevalence of multimorbidity, or associated pregnancy outcomes in women exposed to polypharmacy.

**Conclusion:**

There is a significant burden of polypharmacy among pregnant women. There is a need for evidence on the combinations of medications prescribed in pregnancy, how this specifically affects women with multiple long-term conditions and the associated benefits and harms.

**Tweetable abstract:**

Our systematic review shows significant burden of polypharmacy in pregnancy but outcomes for women and offspring are unknown.

**PROSPERO registration number:**

CRD42021223966.

**Strengths and limitations of this study**

- A structured and substantial review of the literature, according to a preplanned and comprehensive search.
- Articles screened rigorous inclusion and exclusion criteria.
- As there is no consensus definition, polypharmacy was reported according to a variety of definitions in this review.
- Due to the methodological limitations of included studies, it could not be determined whether medications were prescribed concurrently or whether medication was complied with, meaning the prevalence of polypharmacy may have been overestimated.
- No studies reporting on maternal or offspring outcomes associated with polypharmacy were found.

## Introduction

Medications may be taken in pregnancy for the management of pregnancy-related symptoms (such as nausea and vomiting), pre-existing maternal health conditions or pregnancy-related complications.^1–3^ The use of medications among pregnant women has been rising over the past few decades,^4–6^ which could be attributed to a rise in the prevalence of maternal comorbidities, obesity and, in the UK and other high-income countries, a rise in the average maternal age.^7 8^ With this, the use of multiple medications is also likely to increase.^3^ While many studies have assessed overall medication use among pregnant women, fewer studies have focused on polypharmacy.

Polypharmacy is broadly defined as the use of multiple medications by a single patient, but various definitions are found in the literature. A systematic review of polypharmacy definitions found that studies reported various numerical definitions (ranging from the use of two or more medication to eleven or more medications) and some also incorporated duration or appropriateness of therapy.^9^ As the number of medications taken together increases, medication interactions and adverse events are expected to increase also. It has been reported that, as the number of medications prescribed together increases, as does the number of potentially serious drug–drug interactions.^10^ The use of multiple medication has been reported among specific subpopulation of pregnant women, such as women with psychiatric illness, epilepsy or HIV.^11–13^ However, the polypharmacy rate among general population of pregnant women is not as well understood.

Drug pharmacokinetics are altered in pregnancy due to physiological changes in the expectant mothers. For example, expanded plasma volume and maternal body fat in pregnancy increases the volume of distribution for hydrophilic and lipophilic drugs leading to lower plasma concentration. Moreover, increased hepatic and renal clearance during pregnancy can lead to subtherapeutic drug concentrations.^14 15^

However, few clinical trials are undertaken among pregnant women due to concerns around maternal and fetal safety.^16 17^ It is therefore, unknown whether polypharmacy during pregnancy will worsen known side effects, result in novel adverse events or, indeed, have a synergistic or beneficial effect.^10^ Understanding these effects will allow clinicians and women to make more informed decisions about continuing, starting or stopping medications before and during pregnancy.

The objective of this systematic review was to assess the published literature reporting on the prevalence of polypharmacy among pregnant women, the prevalence of multimorbidity in women taking multiple medications in pregnancy and the effect of multiple medication use on maternal and offspring outcomes.

## Methods

A systematic review of the literature was performed in order to identify relevant studies examining the prevalence of polypharmacy in pregnancy, the most common medication combination, rate of multimorbidity and outcomes among women exposed to polypharmacy.

### Protocol and registration

Protocol for this systematic review has been published on PROSPERO (protocol ID CRD42021223966, available from: https://www.crd.york.ac.uk/prospero/display_record.php?ID=CRD42021223966).^18^

### Eligibility criteria

We included interventional trials, observational studies (cohort studies and case–control studies) and systematic reviews reporting the prevalence of polypharmacy or use of multiple medications in pregnant women, where the prevalence of polypharmacy could be extracted from tables or figures. The study authors’ definition of polypharmacy was used and we retained the study authors’ eligibility criteria for whether over-the-counter (OTC) medications were included. Where polypharmacy was not defined by the authors of the individual studies, we defined polypharmacy to mean the use of two or more medications.

#### Exclusion criteria

We excluded studies focused on specific subpopulations of pregnant women instead of general prevalence of polypharmacy (such as pregnant women with specific medical conditions, or with high-risk pregnancies), as we were interested in the population-based prevalence. We excluded expert opinions, conference abstract, case report, narrative review, laboratory and animal studies. Studies based on non-pregnant women were excluded and unpublished data were not sought.

We did not exclude non-English papers. For any non-English paper identified, native speaker would extract data where possible. Where this was not possible, two independent reviewers (AA and AA-L) extracted the data using an online translation service (Google Translate).

### Outcome measurement

The primary outcome was prevalence of polypharmacy, as defined by the authors, or the use of two or more medications, where polypharmacy was not defined by the authors.

We also assessed the prevalence of multimorbidity and maternal or offspring outcomes among women exposed to polypharmacy. The individual studies’ definition of multimorbidity was used where specified. Where the definition of multimorbidity was not specified by the authors, it was defined as the presence of two or more long-term health conditions, including mental health conditions.

### Search strategy

MEDLINE was searched for relevant papers from 1946 to 14 September 2021 and Embase was searched from 1974 to 14 September 2021. A librarian helped to develop the search strategy. The full search strategy for Embase is provided in online supplemental appendix S1.

10.1136/bmjopen-2022-067585.supp1 Supplementary data

### Study selection and data extraction

Study selection was conducted in two phases. In the first phase, title and abstracts were screened by two independent reviewers against the eligibility criteria (AA screened all papers, SIL, AS, AF, UA and ZW were the second reviewers). We retrieved full-text papers for all potentially eligible studies. In the second phase, full-text papers were assessed by two authors independently (AA and AA-L) against the eligibility criteria. For all eligible studies, two authors (AA and AA-L) independently extracted the data using a piloted data extraction form, and assessed the risk of bias. Discrepancies were reviewed and resolved by a third independent reviewer (ZW).

Data items extracted included: purpose of the study, setting, recruitment, inclusion and exclusion criteria, participant demographics (age, ethnicity, parity, deprivation), definition of polypharmacy, prevalence of polypharmacy, classification system for grouping medications, list of health conditions, follow-up length, any secondary outcomes, funding and conflict of interest.

We used the Newcastle-Ottawa critical appraisal checklist for observational studies to assess risk of bias in the individual studies during the data extraction stage.^19^

### Summary measures and results synthesis

Results are presented as descriptive analysis. The primary outcome is presented as proportion or prevalence. We stratified the analysis according to the various definitions of polypharmacy from the primary studies (eg, two or more medications) and the setting (primary or secondary care). Given the heterogenous nature of the studies, statistical pooling and analysis was not possible. Preferred Reporting Items for Systematic Reviews and Meta-Analyses (PRISMA) checklist for reporting of systematic reviews has been followed (online supplemental appendix S2).

10.1136/bmjopen-2022-067585.supp2 Supplementary data

### Patient and public involvement

Patients were not involved in the development of the research question, study design or selection of outcome measures.

## Results

### Study selection

**Figure 1:**
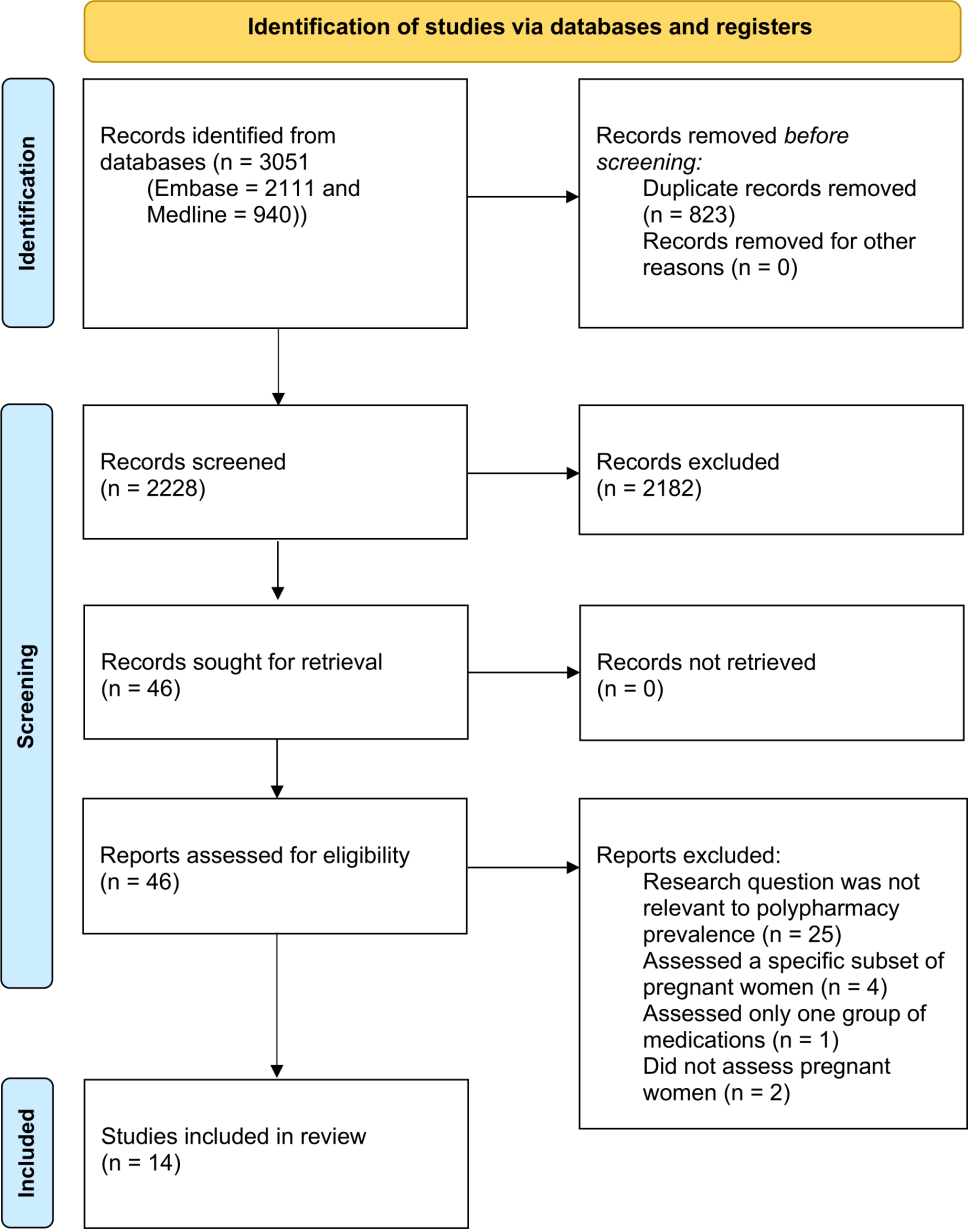
2020 Preferred Reporting Items for Systematic Reviews and Meta-Analyses (PRISMA) flow diagram. Adapted from: Page *et al*.^66^ For more information, visit: http://www.prisma-statement-org/.

We screened 2228 titles and abstracts. Of those, 46 papers were subjected to detailed evaluation in full-text screening,^4 6 20–63^ and 14 met inclusion criteria.^4 6 20–31^ The main reasons for exclusion were an inadequate method of reporting prevalence of polypharmacy or reporting on specific subpopulation of pregnant women. The results from each step of the review process are documented in a PRISMA flow diagram (figure 1).

### Study characteristics

Table 1 shows the characteristics of the included studies. Studies were published between 1991 and 2020. The study populations ranged between 369 and 981 392. Six studies examined the prevalence of polypharmacy using administrative data, seven used surveys to collect self-reported medication use. One study used administrative data for prescription medications and self-report for the use of OTC medications.

**Table 1:** List of included studies and study characteristics

Author	Study design	Country/location	Inclusion criteria	Source (administrative data/self-reported)	Total number of pregnancies	Trimester studied	Polypharmacy definition used in study	Definition of polypharmacy used in review	Medications included or excluded	Prevalence reported
Buitendijk and Bracken^28^	Retrospective survey	USA	All women who made their first prenatal visit to private obstetric or midwifery practice, a health maintenance organisation, or a hospital clinic and were scheduled for delivery at Yale New Haven Hospital	Self-report	4186	Early pregnancy (first trimester)	Polypharmacy not defined by author	≥2	Included OTC medications Excluded vitamins and minerals	33.70%
Olesen *et al* 30	Retrospective cohort	Denmark	Primiparous women identified through Danish National Birth Registry	Administrative data	16 001	Across the three trimesters	More than three medications	≥4 (as defined by the authors)	Excluded vitamins and minerals	2.70%
Gomes *et al* 22	Retrospective survey	Brazil	Pregnant women who gave birth in one of five participating hospitals	Self-report	1620	Across the three trimesters	Polypharmacy not defined by author	>6	Included OTC medications Excluded vitamins and minerals	24.90%
Malm *et al* 24	A retrospective, register-based cohort study	Finland	All women who applied for maternal grants in 1999 and the mother has visited a maternity clinic before the end of the fourth month	Administrative record	43 470	Across the three trimesters	Polypharmacy not defined by author	≥10	Included some, but not all, OTC medications	0.20%
Schirm *et al* 31	Cross-sectional study	Netherlands	Female person (15–50 years older than child) at the same address as child aged 0–5 years, with no other female at the address	Administrative data	7500	Across the three trimesters	Polypharmacy not defined by author	≥2	Excluded OTC medications	62.41%
Refuerzo *et al* 21	Prospective observational	USA	Women who gave birth at a single, university-based, tertiary-care hospital	Self-report	418	Across the three trimesters	Polypharmacy not defined by author	≥2	Included OTC medications	33.50%
Cleary *et al* 26	Retrospective cohort	Ireland	Pregnancy booking and midwife care at tertiary level hospital	Self-report	61 252	Early pregnancy (first trimester)	Polypharmacy not defined by author	≥2	Included OTC medications	11.53%
Mitchell *et al* (NBDPS Study Arm Reported) 4	Cross-sectional study	USA and Canada	NBDPS study controls were randomly selected from birth certificates or from birth hospitals	Self-report	5008	Across the three trimesters	Polypharmacy not defined by author	≥4	Included OTC medications	4.90%
van Gelder *et al* 20	Retrospective cohort study	Netherlands	Female person (15–50 years older than child) at the same address as child aged 0–5 years, with no other female at the address	Administrative record	32 016	First trimester	Polypharmacy not defined by author	≥2	Excluded vitamins and minerals	4.90%
Tinker *et al* 23	Cross-sectional surveys	USA	Non-institutionalised civilian women aged 15–44 years	Self-report	1350	Prior 30 days (pregnancies across three trimesters)	Polypharmacy not defined by author	≥2	Excluded vitamins and minerals	6.10%
Haas *et al* 6	Prospective longitudinal cohort study	USA	Primiparous women, aged 13 years or above, in the first trimester	Self-report	9546	Across the three trimesters	≥5 medications during the same epoch	≥5 (as defined by the authors)	Included OTC medications Analysed medication used when vitamins and minerals included and excluded	13%
Ingstrup *et al* 25	Population-based descriptive study	Denmark	Pregnancies ending in live-born singletons during 1997–2012 to women aged between 15 and 55 years	Administrative record	981 392	Across the three trimesters	Polypharmacy not defined by author	≥2	None mentioned	42.74%
Zhang *et al* 27	Retrospective cohort	China	Singleton deliveries, mothers aged between 12 and 54 years	Administrative data	7946 (2896 pregnancies covering all 3 trimesters)	Across the three trimesters	Polypharmacy not defined by author	≥2	Included OTC medications	9.19%
Obadeji *et al* 29	Cross-sectional study	Nigeria	All consecutive consenting women who came for outpatient antenatal care at a secondary healthcare facility	Administrative data for prescription drug and self-report for OTC	369	Cross-sectional (pregnancies across three trimesters)	Polypharmacy not defined by author	≥3	Included OTC medications	38.30%

NBDPS, National Birth Defects Prevention Study; OTC, over-the-counter.

In seven studies, women were recruited from hospitals (either birth hospital or antenatal clinic).^4 6 21 22 26 28 29^ In the other seven studies, participants were sampled from a national registry or population-based database (such as pharmacy records).^20 23–25 27 30 31^

Mitchell *et al* reported results from two different cohorts: Birth Defect Study (BDS) and National Birth Defects Prevention Study (NBDPS). Both studies contain data from mothers of babies born with birth defects and from a control group of mothers of babies born without birth defects. Mitchell *et al* reported data from both cases and controls in the BDS and from just the controls of the NBDPS. As pregnancies of mothers of babies born with birth defects are unlikely to be representative of the general population of pregnant women, only data from NBDPS were included in the results of this review.

### Risk of bias within studies

Most of the study cohorts were considered representative of the population they were sampling from. Most studies ascertained pregnancy status using hospital or pharmacy records or from birth registries, which were considered likely to be accurate. van Gelder *et al* and Schirm *et al* used a pharmacy database to identify all children born within a given timeframe.^20 31^ Women of reproductive age living at the same address as the child were identified in the database and their prescription data was collected for the 270 days before the child’s date of birth. There is a chance that women could have been misclassified as pregnant if the child was not living with their biological mother.

As discussed above, seven studies relied solely on self-reported medication use to measure outcomes, introducing the potential for recall bias.^4 6 21 22 26 28 29^ The follow-up period was considered adequate for each study. Nine studies reported multiple medication use across the entire pregnancy,^4 6 20 21 23 24 26 29 30^ while three studies reported for early pregnancy (first trimester) only.^19 25 27^ Obadeji *et al* and Tinker *et al* employed a cross-sectional design and included women across all trimesters.^23 29^ Follow-up rates were considered adequate for all studies, with no study having significant numbers of subjects lost to follow-up. Online supplemental table S1 shows the outcome of the risk of bias assessment.

10.1136/bmjopen-2022-067585.supp3 Supplementary data

### Prevalence of polypharmacy

The prevalence of polypharmacy ranged from 0.2% to 62.4%, with a median value of 12.3%. The exclusion of OTC drugs does not change the spread of the prevalence of polypharmacy.

#### Prevalence by polypharmacy definition

**Figure 2:**
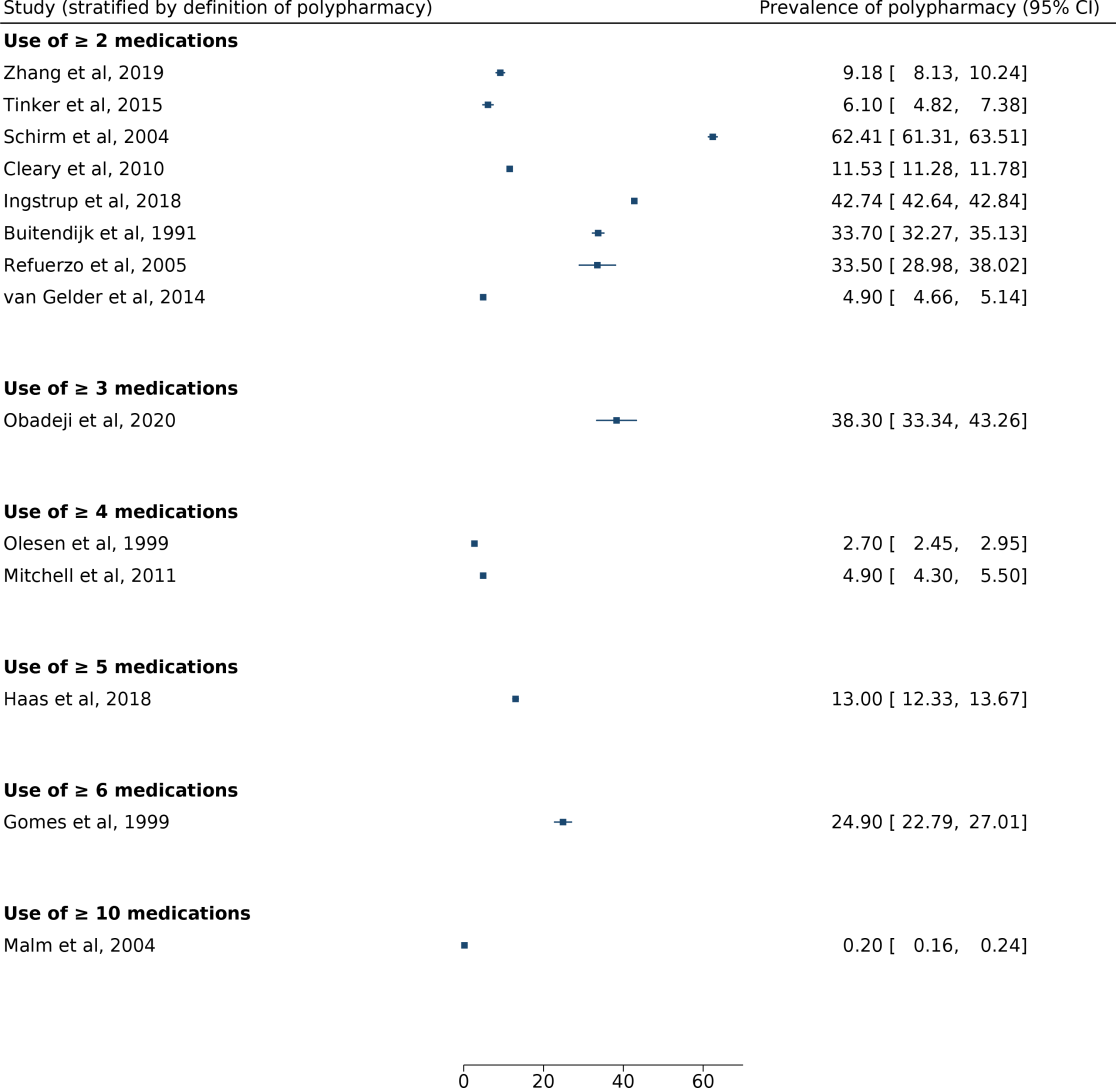
Forest plot showing prevalence of polypharmacy, subdivided by the definition of polypharmacy (number of medications taken).

The prevalence of polypharmacy, defined as the use or two or more medications, ranged from 4.9% (4.3%–5.5%) to 61.3% (61.3%–63.5%) based on eight papers, with a median value of 22.5%^20 21 23 25–28 31^ (figure 2). Only two studies explicitly defined polypharmacy. Olesen *et al* defined it as the use of four or more medications (prevalence 2.7%) and Haas *et al* defined it as the use of five or more medications (prevalence 13%).^6 30^

Other studies did not define polypharmacy, but stratified results by the number of medications taken (figure 2). Mitchell *et al* and Gomes *et al* did not define polypharmacy and only reported the use of four or more medications (15.7%) and six or more drugs (24.9%), respectively.^4 22^ Malm *et al* reported that 0.2% of women purchased 10 or more different medications during the whole period of pregnancy.^24^ Due to heterogeneity within the data, meta-analysis was not undertaken.

#### Prevalence of polypharmacy by trimester

Two studies, Obadeji *et al* and Zhang *et al*, reported polypharmacy use across the whole pregnancy and also subdivided into trimesters. For these two studies, polypharmacy prevalence across the whole pregnancy has been summarised.^27 29^ Obadeji *et al* reported a prevalence of 50.0% (95% CI 21.1% to 79.0%) in the first trimester compared with a prevalence of 38.3% (95% CI 33.4% to 43.26%) across all three trimesters. Zhang *et al* reported a prevalence of 3.8%% (95% CI 3.1% to 4.6%) in the first trimester compared with a prevalence of 9.2% (95% CI 8.3% to 10.2%) across all three trimesters.

**Figure 3:**
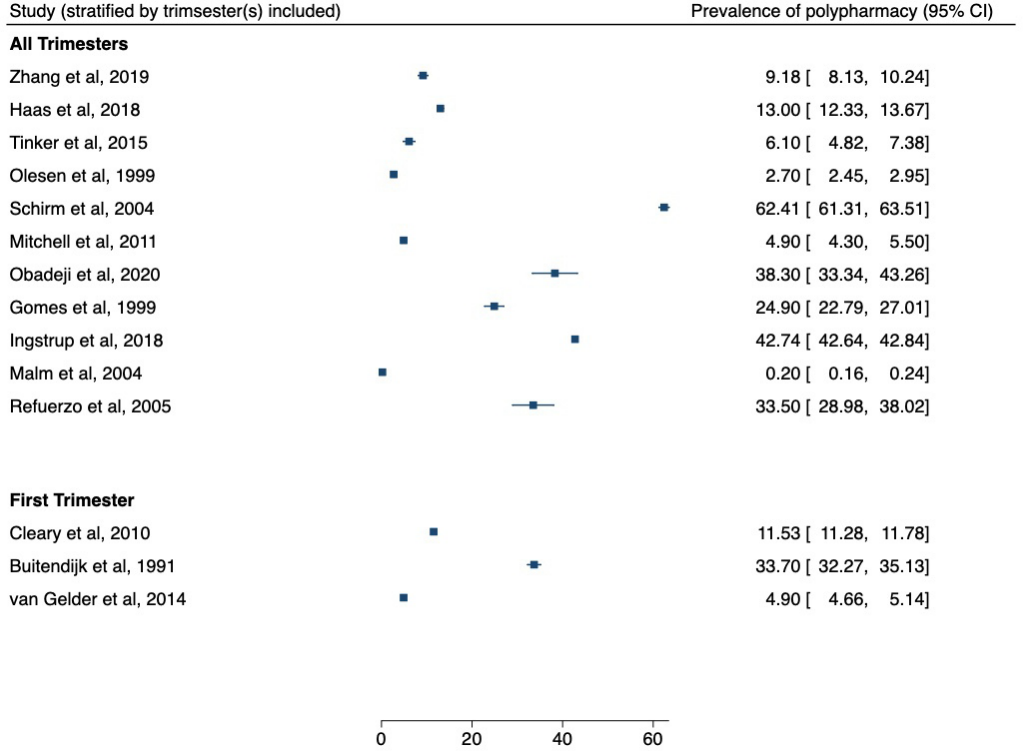
Forest plot showing prevalence of polypharmacy (as defined by the study), for studies which covered all trimesters of the pregnancy and the first trimester.

Due to the design and nature of the study, Van Gelder *et al*, Cleary *et al* and Buitendijk *et al* have reported medication use during early pregnancy or the first trimester period only, reporting polypharmacy prevalence of 4.9% (95% CI 4.7% to 5.1%), 11.5% (95% CI 11.3% to 11.8%) and 33.7% (95% CI 32.2% to 35.1%).^20 28^ In a cross-sectional study, Tinker *et al* cover medication use in the last 30 days only but across the whole pregnancy.^23^ Olesen *et al* cover a period from 12 weeks prenatal to 12 weeks postpartum in the analysis.^30^ Figure 3 shows polypharmacy prevalence when including studies which covered the entire duration of pregnancy.

#### Prevalence of polypharmacy by medications included

While most of the studies reported any possible medication use, van Gelder *et al* report only the teratogenic medications used and not all possible medications.^20^

#### OTC medications

**Figure 4:**
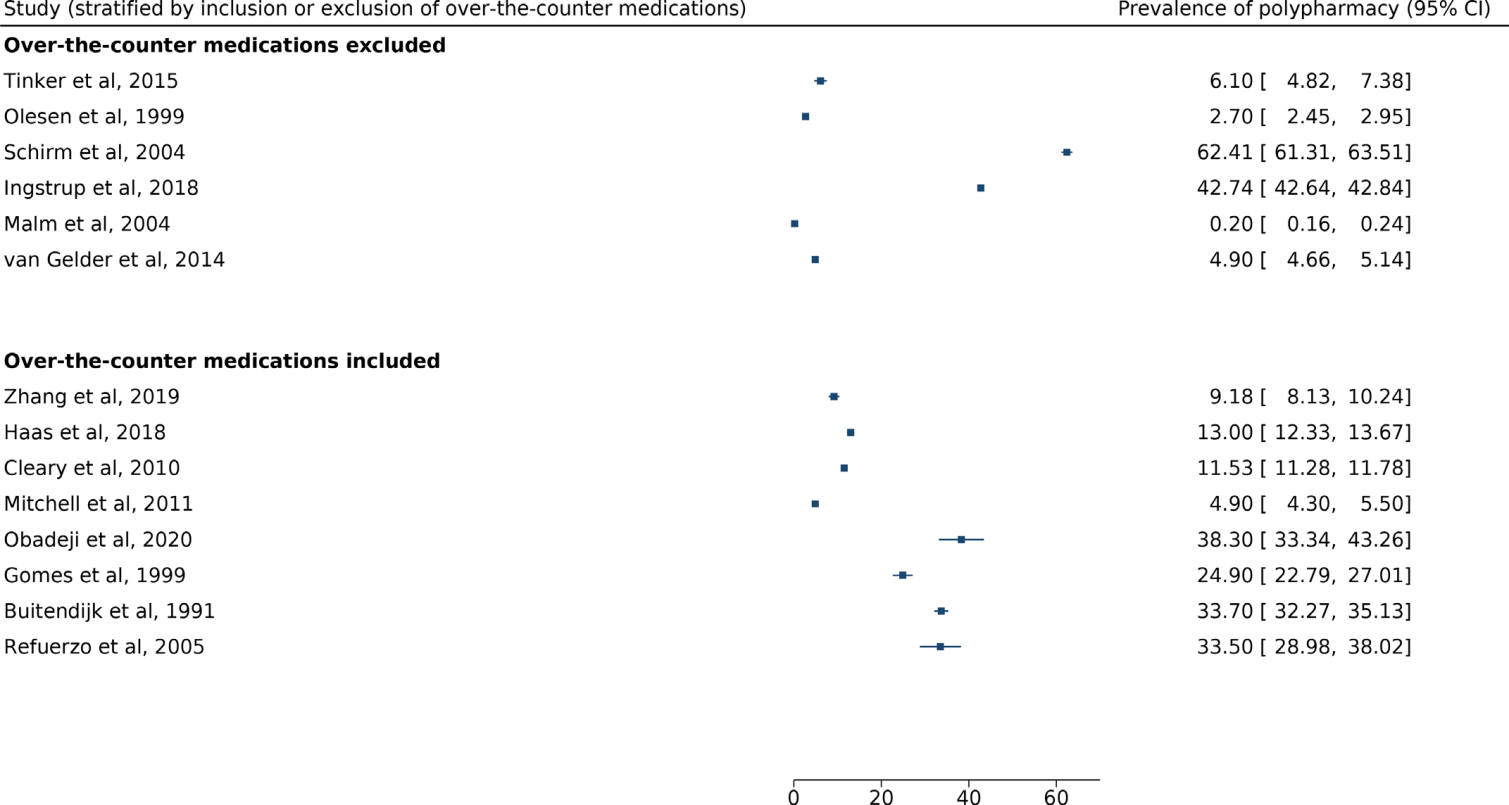
Forest plot showing prevalence of polypharmacy, subdivided by inclusion or exclusion of over-the-counter medications.

Eight studies include OTC medications in their analysis—results for polypharmacy prevalence, subdivided by inclusion of OTC drugs, are shown in figure 4.^4 6 21 22 26–29^ Reported prevalence of polypharmacy for studies that included OTC medications ranged from 4.9% (Mitchell *et al* (95% CI 4.3% to 5.5%)) to 38.3% (Obadeji *et al* (95% CI 33.3% to 43.3%)). Reported prevalence of polypharmacy for studies that excluded OTC medications ranged from 0.2% (Malm *et al* (95% CI 0.2% to 0.2%) to 62.4% (Schirm *et al* (95% CI 61.3% to 63.5%)). Of note, Malm *et al* include some but not all OTC medications, as some medications were reimbursable and therefore were included in the national medication prescription register used for the study.^24^

#### Exclusion of vitamins and minerals

Five studies specifically excluded vitamins and minerals (such as folic acid and iron) from the study design.^20 22 23 28 30^ The definition of routine prenatal vitamins or minerals was determined by the authors of the original studies. Haas *et al* analysed medication use, when vitamins and minerals were included and excluded. When including vitamins and minerals, Haas *et al* report 30.5% (95% CI 29.6% to 31.5%) of women use five or more medication; whereas, only 13% (95% CI 12.3% to 13.7%) use five or more medications if vitamins and minerals are excluded.^6^

### Medications used during pregnancy

The most commonly prescribed or taken medications described in the studies were antiemetics,^4 6 23^ antibiotics^4 6 27–31^ analgesia^4 6 23^ and antacids^23 29 31^ and vitamins or supplements^6 28 31^ However, no studies specified which medications were used in combination or were used by women exposed to polypharmacy.

### Multimorbidity and maternal or offspring outcomes

No studies were found describing which conditions women who were exposed to polypharmacy were treated for, and none specify how many women had multimorbidity or long-term illness. No studies were found that reported on maternal or offspring outcomes.

## Discussion

### Main findings

Studies of multiple medication use in pregnancy reported a wide range in the prevalence of polypharmacy. Where the definition of polypharmacy was two or more medications only, the prevalence of polypharmacy ranged from 5% to 62%. However, the definition of polypharmacy was varied, and most studies were not considered truly representative of all pregnant women.

### Strengths and limitations

This systematic review has several important strengths. We developed a structured and substantial review of the literature, according to preplanned and comprehensive search terms with the help of a librarian, who is trained to undertake searches in large database repositories. Screening was conducted according to a rigorous inclusion and exclusion criteria, and we used two independent reviewers for data extraction to minimise bias. Two databases were searched: MEDLINE and Embase. We did not limit our search to studies published in the English language to minimise language bias, although specific databases in languages other than English were not included.

There are limited studies specifically assessing polypharmacy in pregnancy. There is no consensus on the definition of polypharmacy and polypharmacy is often not explicitly defined in the studies. Where polypharmacy is defined, the definition varies from study to study. Only two studies in this systematic review subdivide polypharmacy use in different trimesters. Exclusion of routine prenatal vitamins is often determined by individual authors. Inclusion of OTC medications is variable and often determined by the data available.

The main caveat from these studies is that it is not clear whether the use of multiple medication in pregnancy was simultaneous or sequential. Additionally, prescription and dispensation of medications do not equate to compliance. Qualitative studies show that women are less likely to use medications when pregnant, especially if potential risks to the fetus and benefits to the mother have not been adequately communicated.^64^

In majority of the studies identified in this systematic review, pregnancy was confirmed retrospectively or identified using birth records. Thus, not all pregnancies were captured and pregnancies resulting in terminations, miscarriages or stillbirth, were excluded. These pregnancy outcomes are clinically important and the use of multiple medications in these groups warrants further assessment.

While some of the studies outline common medications used by pregnant women overall, none of the studies describe the combinations of medications used in pregnancy. Pregnant women have been described as drug orphans, as they are often excluded from clinical trials. The maternal and offspring outcomes following medication exposure during pregnancy are often determined through retrospective observational studies.^16 17^ The association between rates of miscarriage and preterm birth and medications used during pregnancy have been described in women with major psychiatric illnesses^13^; however, none of the studies assessing polypharmacy in this systematic review evaluate the effect of taking multiple medication for the women and their offspring.

### Interpretation

The finding of 5%–62% of pregnant women taking two or more medications is in keeping with a previous systematic review of the literature evaluating individual-level exposures to prescription medications in pregnancy. This review, which included only studies from developed (Organisation for Economic Co-operation and Development (OECD)) countries, found 27%–93% of women filled at least one prescription during pregnancy reflecting high medication use during pregnancy.^65^

The findings of this review should be interpreted with caution. As discussed above, the literature is not necessarily representative of the general pregnant population, inclusion of certain medications was variable and, where polypharmacy was defined, there were differences in the definitions used. This variation is in keeping with the findings of a systematic review of definitions of polypharmacy in older people.^9^ This review also found that, in some instances, safety and appropriateness of medications were taken into account when defining polypharmacy. This is an important consideration in pregnancy, although, as discussed, there is often not adequate safety information available.

Despite this, the median value of one in five women taking two or more medications, indicates that a significant proportion of women are potentially exposed to multiple medication in pregnancy. The lack of studies into combinations of medications taken during pregnancy and the effects of polypharmacy on maternal and offspring outcomes highlights the urgent need for further research in this area.

## Conclusion

The reported prevalence of polypharmacy among pregnant women varies based on the number of medications counted in the definition, the trimester considered and the types of medications included. Commonly, only pregnancies resulting in live birth are reported in studies assessing polypharmacy. This systematic review shows relatively large burden of polypharmacy among pregnant women and highlights the need to evaluate the outcomes for these women and for their offspring. This is especially relevant for women with multiple, long-term conditions, who are more likely to need multiple medications.

## Supplementary Material

Reviewer comments

Author's manuscript

## Data Availability

No data are available.

## References

[1] Kulkarni J , Worsley R , Gilbert H , et al . A prospective cohort study of antipsychotic medications in pregnancy: the first 147 pregnancies and 100 one year old babies. PLoS ONE 2014;9:e94788. 10.1371/journal.pone.0094788 24787688 PMC4008497

[2] Beeson JG , Homer CSE , Morgan C , et al . Multiple morbidities in pregnancy: time for research, innovation, and action. PLOS Med 2018;15:e1002665. 10.1371/journal.pmed.1002665 30252846 PMC6155445

[3] Barnett K , Mercer SW , Norbury M , et al . Epidemiology of multimorbidity and implications for health care, research, and medical education: a cross-sectional study. Lancet 2012;380:37–43. 10.1016/S0140-6736(12)60240-2 22579043

[4] Mitchell AA , Gilboa SM , Werler MM , et al . Medication use during pregnancy, with particular focus on prescription drugs: 1976-2008. Am J Obstet Gynecol 2011;205:51. 10.1016/j.ajog.2011.02.029 PMC3793635 21514558

[5] Headley J , Northstone K , Simmons H , et al . Medication use during pregnancy: data from the Avon longitudinal study of parents and children. Eur J Clin Pharmacol 2004;60:355–61. 10.1007/s00228-004-0775-7 15168103

[6] Haas DM , Marsh DJ , Dang DT , et al . Prescription and other medication use in pregnancy. Obstet Gynecol 2018;131:789–98. 10.1097/AOG.0000000000002579 29630018 PMC5912972

[7] Office for National Statistics . Birth characteristics in england and wales: 2019. 2019. Available: https://www.ons.gov.uk/peoplepopulationandcommunity/birthsdeathsandmarriages/livebirths/bulletins/birthcharacteristicsinenglandandwales/2019

[8] MuM-PreDiCT . Epidemiology of pre-existing multimorbidity in pregnant women in the UK in 2018: a cross sectional study using CPRD SAIL and SMR. 2021. Available: https://docs.google.com/document/d/1mZf9YSqCIZIX8Og2ROy9epgloYQlabtq/edit 10.1186/s12884-022-04442-3 PMC8840793 35148719

[9] Masnoon N , Shakib S , Kalisch-Ellett L , et al . What is polypharmacy? A systematic review of definitions. BMC Geriatr 2017;17:230. 10.1186/s12877-017-0621-2 29017448 PMC5635569

[10] Guthrie B , Makubate B , Hernandez-Santiago V , et al . The rising tide of polypharmacy and drug-drug interactions: population database analysis 1995-2010. BMC Med 2015;13:74. 10.1186/s12916-015-0322-7 25889849 PMC4417329

[11] Okoli C , Schwenk A , Radford M , et al . Polypharmacy and potential drug-drug interactions for people with HIV in the UK from the climate-HIV database. HIV Med 2020;21:471–80. 10.1111/hiv.12879 32671950 PMC7497154

[12] Kinney MO , Morrow J . Epilepsy in pregnancy. BMJ 2016;353:i2880. 10.1136/bmj.i2880 27255543

[13] Peindl KS , Masand P , Mannelli P , et al . Polypharmacy in pregnant women with major psychiatric illness: a pilot study. J Psychiatr Pract 2007;13:385–92. 10.1097/01.pra.0000300124.83945.b8 18032983

[14] Feghali M , Venkataramanan R , Caritis S . Pharmacokinetics of drugs in pregnancy. Semin Perinatol 2015;39:512–9. 10.1053/j.semperi.2015.08.003 26452316 PMC4809631

[15] Pariente G , Leibson T , Carls A , et al . Pregnancy-associated changes in pharmacokinetics: a systematic review. PLoS Med 2016;13:e1002160. 10.1371/journal.pmed.1002160 27802281 PMC5089741

[16] Scaffidi J , Mol BW , Keelan JA . The pregnant women as a drug orphan: a global survey of registered clinical trials of pharmacological interventions in pregnancy. BJOG 2017;124:132–40. 10.1111/1471-0528.14151 27297096

[17] Illamola SM , Bucci-Rechtweg C , Costantine MM , et al . Inclusion of pregnant and breastfeeding women in research-efforts and initiatives. Br J Clin Pharmacol 2018;84:215–22. 10.1111/bcp.13438 28925019 PMC5777434

[18] Astha Anand AS , Lee S , Nirantharakumar K , et al . Prevalence of polypharmacy in pregnancy and associated health outcomes in mothers and offspring crd.york.ac.uk: propero (national institute for health research). 2021. Available: https://www.crd.york.ac.uk/prospero/display_record.php?ID=CRD42021223966)

[19] Wells G , Shea B , O’Connell D , et al . The newcastle-ottawa scale (NOS) for assessing the quality of nonrandomised studies in meta-analyses. 2013. Available: http://www.ohri.ca/programs/clinical_epidemiology/oxford.asp

[20] van Gelder MMHJ , Bos JHJ , Roeleveld N , et al . Drugs associated with teratogenic mechanisms. Part I: dispensing rates among pregnant women in the Netherlands, 1998-2009. Hum Reprod 2014;29:161–7. 10.1093/humrep/det369 24105826

[21] Refuerzo JS , Blackwell SC , Sokol RJ , et al . Use of over-the-counter medications and herbal remedies in pregnancy. Am J Perinatol 2005;22:321–4. 10.1055/s-2005-873235 16118721

[22] Gomes KR , Moron AF , Silva R , et al . Prevalence of use of medicines during pregnancy and its relationship to maternal factors. Rev Saude Publica 1999;33:246–54. 10.1590/s0034-89101999000300005 10456997

[23] Tinker SC , Broussard CS , Frey MT , et al . Prevalence of prescription medication use among non-pregnant women of childbearing age and pregnant women in the United States: NHANES, 1999-2006. Matern Child Health J 2015;19:1097–106. 10.1007/s10995-014-1611-z 25287251 PMC4515960

[24] Malm H , Martikainen J , Klaukka T , et al . Prescription of hazardous drugs during pregnancy. Drug Saf 2004;27:899–908. 10.2165/00002018-200427120-00006 15366977

[25] Ingstrup KG , Liu X , Gasse C , et al . Prescription drug use in pregnancy and variations according to prior psychiatric history. Pharmacoepidemiol Drug Saf 2018;27:105–13. 10.1002/pds.4355 29171114

[26] Cleary BJ , Butt H , Strawbridge JD , et al . Medication use in early pregnancy-prevalence and determinants of use in a prospective cohort of women. Pharmacoepidemiol Drug Saf 2010;19:408–17. 10.1002/pds.1906 20099251

[27] Zhang J , Ung COL , Wagner AK , et al . Medication use during pregnancy in mainland China: a cross-sectional analysis of a national health insurance database. Clin Epidemiol 2019;11:1057–65. 10.2147/CLEP.S230589 31849536 PMC6911329

[28] Buitendijk S , Bracken MB . Medication in early pregnancy: prevalence of use and relationship to maternal characteristics. Am J Obstet Gynecol 1991;165:33–40. 10.1016/0002-9378(91)90218-g 1853911

[29] Obadeji ST , Obadeji A , Bamidele JO , et al . Medication use among pregnant women at a secondary health institution: utilisation patterns and predictors of quantity. Afr Health Sci 2020;20:1206–16. 10.4314/ahs.v20i3.24 33402967 PMC7751527

[30] Olesen C , Steffensen FH , Nielsen GL , et al . Drug use in first pregnancy and lactation: a population-based survey among Danish women. The euromap group. Eur J Clin Pharmacol 1999;55:139–44. 10.1007/s002280050608 10335909

[31] Schirm E , Meijer WM , Tobi H , et al . Drug use by pregnant women and comparable non-pregnant women in the Netherlands with reference to the Australian classification system. Eur J Obstet Gynecol Reprod Biol 2004;114:182–8. 10.1016/j.ejogrb.2003.10.024 15140513

[32] Alani AHHDA , Hassan BAR , Suhaimi AM , et al . Use, awareness, knowledge and beliefs of medication during pregnancy in Malaysia. Osong Public Health Res Perspect 2020;11:373–9. 10.24171/j.phrp.2020.11.6.05 33403200 PMC7752143

[33] Zaki NM , Albarraq AA . Use, attitudes and knowledge of medications among pregnant women: a Saudi study. Saudi Pharm J 2014;22:419–28. 10.1016/j.jsps.2013.09.001 25473330 PMC4246410

[34] Handal M , Engeland A , Rønning M , et al . Use of prescribed opioid analgesics and co-medication with benzodiazepines in women before, during, and after pregnancy: a population-based cohort study. Eur J Clin Pharmacol 2011;67:953–60. 10.1007/s00228-011-1030-7 21484468

[35] Nordeng H , Bayne K , Havnen GC , et al . Use of herbal drugs during pregnancy among 600 norwegian women in relation to concurrent use of conventional drugs and pregnancy outcome. Complement Ther Clin Pract 2011;17:147–51. 10.1016/j.ctcp.2010.09.002 21742280

[36] Hanley GE , Park M , Oberlander TF . Socieconomic status and psychotropic medicine use during pregnancy: a population-based study in British Columbia, Canada. Arch Womens Ment Health 2020;23:689–97. 10.1007/s00737-020-01034-y 32409987

[37] Truong BT , Lupattelli A , Kristensen P , et al . Sick leave and medication use in pregnancy: a European web-based study. BMJ Open 2017;7:e014934. 10.1136/bmjopen-2016-014934 PMC5724093 28775180

[38] Rouamba T , Valea I , Bognini JD , et al . Safety profile of drug use during pregnancy at peripheral health centres in burkina faso: a prospective observational cohort study. Drugs Real World Outcomes 2018;5:193–206. 10.1007/s40801-018-0141-1 30155832 PMC6119166

[39] Zhang J , Ung COL , Guan X , et al . Safety of medication use during pregnancy in mainland China: based on a national health insurance database in 2015. BMC Pregnancy Childbirth 2019;19. 10.1186/s12884-019-2622-y PMC6892234 31795963

[40] Bérard A , Sheehy O . The quebec pregnancy cohort -- prevalence of medication use during gestation and pregnancy outcomes. PLoS One 2014;9:e93870. 10.1371/journal.pone.0093870 24705674 PMC3976411

[41] Farooq MO , Reddy SK , Raghu Prasada MS , et al . Prescription pattern of the drugs among pregnant inpatients in tertiary care hospital. J Pharm Res 2014;8:981–5.

[42] Rathod AM , Rathod RM , Jha RK , et al . Prescribing trends in antenatal care at a tertiary level teaching hospital of Vidarbha region. Res J Pharm Biol Chem Sci 2012;3:865–72.

[43] Agarwal M , Nayeem M , Safhi MM , et al . Prescribing pattern of drugs in the department of obstetrics and gynecology in expected mothers in Jazan Region, KSA. Int J Pharm Pharm Sci 2014;6:658–61.

[44] Makiabadi F , Rajeswari R , Jayashree AK . Prescribing pattern of drugs in department of obstetrics and gynecology at a tertiary care teaching hospital, Bangalore, India. PJMHS 2021;15:1265–9. 10.53350/pjmhs211551265

[45] Vafai Y , Yeung EH , Sundaram R , et al . Prenatal medication use in a prospective pregnancy cohort by pre-pregnancy obesity status. J Matern Fetal Neonatal Med 2022;35:5799–806. 10.1080/14767058.2021.1893296 33706661 PMC8802334

[46] Sripada R , Suresh Kumar SV , Devanna N , et al . Pattern of possible drug-drug interactions among different specialties at an indian tertiary care teaching hospital. Ijrps 2020;11:3988–92. 10.26452/ijrps.v11i3.2591

[47] Gharoro EP , Igbafe AA . Pattern of drug use amongst antenatal patients in Benin City, Nigeria. Med Sci Monit 2000;6:84–7. 11208289

[48] Lee E , Maneno MK , Smith L , et al . National patterns of medication use during pregnancy. Pharmacoepidemiol Drug Saf 2006;15:537–45. 10.1002/pds.1241 16700083

[49] Palmsten K , Hernández-Díaz S , Chambers CD , et al . The most commonly dispensed prescription medications among pregnant women enrolled in the U.S. Medicaid program. Obstet Gynecol 2015;126:465–73. 10.1097/AOG.0000000000000982 26244530 PMC4651654

[50] Havard A , Barbieri S , Hanly M , et al . Medications used disproportionately during pregnancy: priorities for research on the risks and benefits of medications when used during pregnancy. Pharmacoepidemiol Drug Saf 2021;30:53–64. 10.1002/pds.5131 32935407

[51] Glavind J , Greve T , de Wolff MG , et al . Medication used in Denmark in the latent phase of labor-do we know what we are doing? Sex Reprod Healthc 2020;25:100515. 10.1016/j.srhc.2020.100515 32361536

[52] de Jonge L , Zetstra-van der Woude PA , Bos HJ , et al . Identifying associations between maternal medication use and birth defects using a case-population approach: an exploratory study on signal detection. Drug Saf 2013;36:1069–78. 10.1007/s40264-013-0082-2 23828658

[53] Ilic M , Nordeng H , Lupattelli A . Medical care contact for infertility and related medication use during pregnancy – a European, cross-sectional web-based study. Nor J Epidemiol 2021;29:97–106. 10.5324/nje.v29i1-2.4051

[54] Baraka MA , Steurbaut S , Coomans D , et al . Ethnic differences in drug utilization pattern during pregnancy: a cross-sectional study. J Matern Fetal Neonatal Med 2013;26:900–7. 10.3109/14767058.2013.765843 23350574

[55] Irvine L , Flynn RWV , Libby G , et al . Drugs dispensed in primary care during pregnancy: a record-linkage analysis in Tayside, Scotland. Drug Saf 2010;33:593–604. 10.2165/11532330-000000000-00000 20553060

[56] Araujo M , Hurault-Delarue C , Sommet A , et al . Drug prescriptions in French pregnant women between 2015 and 2016: a study in the EGB database. Therapies 2021;76:239–47. 10.1016/j.therap.2020.07.002 32736872

[57] Girit N , Tugrul I , Demirci B , et al . Drug exposure in early pregnancy might be related to the effects of increased maternal progesterone in implantation period. J Psychosom Obstet Gynaecol 2018;39:7–10. 10.1080/0167482X.2017.1289370 28635531

[58] Bornhauser C , Quack Lotscher Katharina C , Seifert B , et al . Diet, medication use and drug intake during pregnancy: data from the consecutive Swiss health surveys of 2007 and 2012. Swiss Med Wkly 2017;147:51–2. 10.4414/smw.2017.14572 29282704

[59] Galappatthy P , Ranasinghe P , Liyanage CK , et al . Core prescribing indicators and the most commonly prescribed medicines in a tertiary health care setting in a developing country. Adv Pharmacol Pharm Sci 2021;2021:6625377. 10.1155/2021/6625377 33564747 PMC7867447

[60] Merlob P , Stahl B , Kaplan B . Children born to mothers using multiple drug therapy during their pregnancy. Int J Risk Saf Med 1996;8:237–41. 10.3233/JRS-1996-8307 23511983

[61] Eze UI , Eferakeya AE , Oparah AC , et al . Assessment of prescription profile of pregnant women visiting antenatal clinics. Pharm Pract (Granada) 2007;5:135–9. 10.4321/s1886-36552007000300007 25214930 PMC4154748

[62] Belay M , Kahaliw W , Ergetie Z . Assessment of drug utilization pattern during pregnancy in adama riferral hospital, Oromia region, Ethiopia. Int J Pharm Sci Res 2013;4:1905–11.

[63] van Gelder MMHJ , Vorstenbosch S , Te Winkel B , et al . Using web-based questionnaires to assess medication use during pregnancy: a validation study in 2 prospectively enrolled cohorts. Am J Epidemiol 2018;187:326–36. 10.1093/aje/kwx239 29401360

[64] Lynch MM , Amoozegar JB , McClure EM , et al . Improving safe use of medications during pregnancy: the roles of patients, physicians, and pharmacists. Qual Health Res 2017;27:2071–80. 10.1177/1049732317732027 28974142 PMC5819595

[65] Daw JR , Hanley GE , Greyson DL , et al . Prescription drug use during pregnancy in developed countries: a systematic review. Pharmacoepidemiol Drug Saf 2011;20:895–902. 10.1002/pds.2184 21774029 PMC3423446

[66] Page MJ , McKenzie JE , Bossuyt PM , et al . The PRISMA 2020 statement: an updated guideline for reporting systematic reviews. BMJ 2021;372:71. 10.1136/bmj.n71 PMC8005924 33782057

