# Supplementary material for: Prevalence of polypharmacy in pregnancy: a systematic review
Source: BMJ Open. 2023 Mar 6;13(3):e067585. doi: 10.1136/bmjopen-2022-067585 (PMC9990613; doi:10.1136/bmjopen-2022-067585)
Supplement: Supplementary data [file bmjopen-2022-067585supp001.pdf]

Appendix 1 – Search strategy

The search strategy for Embase and MEDLINE is shown below.

1. polypharmacy/
2. multiple medicatio*.mp.
3. multiple medicine*.mp.
4. multiple drug*.mp.
5. many medicatio*.mp.
6. many medicine*.mp.
7. many drug*.mp.
8. (more adj4 medication*).mp.
9. polydrug*.mp.
10. polymedication.mp.
11. polypharmacy.mp.
12. multi-drug therapy.mp.
13. multidrug therapy.mp.
14. multiple pharmacotherapy.mp.
15. poly pharmacy.mp.
16. polypragmasia.mp.
17. polypragmasy.mp.
18. exp pregnancy/
19. exp Pregnancy Complications/ or exp Pregnancy Disorders/
20. pregnan*.mp.
21. mothers/
22. perinatal.mp.
23. maternal.mp.
24. obstetric*.mp.
25. or/1-17
26. or/18-24
27. 25 and 26
