# Supplementary material for: Prevalence of polypharmacy in pregnancy: a systematic review
Source: BMJ Open. 2023 Mar 6;13(3):e067585. doi: 10.1136/bmjopen-2022-067585 (PMC9990613; doi:10.1136/bmjopen-2022-067585)
Supplement: Supplementary data [file bmjopen-2022-067585supp003.pdf]

Table S1- Summary of Newcastle-Ottawa Quality Assessment Scale Score for Included Studies

	Selection		Outcome		
**Author**	**Representativeness of the cohort**	**Ascertainment of pregnancy**	**Assessment of polypharmacy**	**Was follow-up long enough**	**Adequacy of follow-up**
**Buitendijk 1991 (29)**	*	*	-	*	*
**Olesen 1998 (31)**	*	*	*	*	*
**Gomes 1999 (22)**	*	*	-	*	*
**Malm 2004 (24)**	*	*	*	*	*
**Schirm 2004 (32)**	*	-	*	*	*
**Refuerzo 2005 (21)**	*	*	-	*	*
**Cleary 2010 (26)**	*	*	-	*	*
**Mitchell 2011 (27)**	*	*	-	*	*
**Van Gelder 2014 (20)**	*	-	*	*	*
**Tinker 2016 (23)**	-	-	-	*	*
**Haas 2018 (6)**	*	*	-	*	*
**Ingstrup 2018 (24)**	*	*	*	*	*
**Zhang 2019 (27)**	*	*	*	*	*
**Obadeji 2020 (29)**	*	*	*	*	*

* Indicates adequate quality in domain. A maximum of one star can be given for each domain
