# Supplementary material for: Prevalence of polypharmacy in pregnancy: a systematic review
Source: BMJ Open. 2023 Mar 6;13(3):e067585. doi: 10.1136/bmjopen-2022-067585 (PMC9990613; doi:10.1136/bmjopen-2022-067585)
Supplement: Author's manuscript [file bmjopen-2022-067585.draft_revisions.pdf]

BMJ Open

BMJ Open is committed to open peer review. As part of this commitment we make the peer review history of every article we publish publicly available.

When an article is published we post the peer reviewers' comments and the authors' responses online. We also post the versions of the paper that were used during peer review. These are the versions that the peer review comments apply to.

The versions of the paper that follow are the versions that were submitted during the peer review process. They are not the versions of record or the final published versions. They should not be cited or distributed as the published version of this manuscript.

BMJ Open is an open access journal and the full, final, typeset and author-corrected version of record of the manuscript is available on our site with no access controls, subscription charges or pay-per-view fees (http://bmjopen.bmj.com).

If you have any questions on BMJ Open's open peer review process please email info.bmjopen@bmj.com

BMJ Open

Prevalence of polypharmacy in pregnancy: a systematic review

Journal:	*BMJ Open*
Manuscript ID	bmjopen-2022-067585
Article Type:	Original research
Date Submitted by the Author:	18-Aug-2022
Complete List of Authors:	Anand, Astha; University of Birmingham Institute of Applied Health Research<br>Phillips, Katherine; University of Birmingham, Applied health research<br>Subramanian, Anuradhaa; University of Birmingham Institute of Applied Health Research<br>Lee, Siang Ing; University of Birmingham Institute of Applied Health Research<br>Wang, Zhaonan; University of Birmingham Institute of Applied Health Research<br>McCowan, Rebecca; University of Glasgow<br>Agrawal, Utkarsh; University of St Andrews, School of Medicine<br>Fagbamigbe, Adeniyi; University of St Andrews, PBS; University of Ibadan, EMS<br>Nelson-Piercy, Catherine; Guy's and St Thomas' NHS Foundation Trust<br>Brocklehurst, Peter; University of Birmingham Institute of Applied Health Research<br>Damase-Michel, Christine; CHU Toulouse, CHU Toulouse, Université Toulouse III<br>Loane , Maria ; University of Ulster Institute of Nursing and Health Research<br>Nirantharakumar, Krishnarajah; University of Birmingham Institute of Applied Health Research<br>Azcoaga-Lorenzo, Amaya; University of Saint Andrews School of Medicine, Division of Population and Behavioural Sciences
Keywords:	OBSTETRICS, Maternal medicine < OBSTETRICS, EPIDEMIOLOGY

SCHOLARONE™
Manuscripts

I, the Submitting Author has the right to grant and does grant on behalf of all authors of the Work (as defined in the below author licence), an exclusive licence and/or a non-exclusive licence for contributions from authors who are: i) UK Crown employees; ii) where BMJ has agreed a CC-BY licence shall apply, and/or iii) in accordance with the terms applicable for US Federal Government officers or employees acting as part of their official duties; on a worldwide, perpetual, irrevocable, royalty-free basis to BMJ Publishing Group Ltd ("BMJ") its licensees and where the relevant Journal is co-owned by BMJ to the co-owners of the Journal, to publish the Work in this journal and any other BMJ products and to exploit all rights, as set out in our licence.

The Submitting Author accepts and understands that any supply made under these terms is made by BMJ to the Submitting Author unless you are acting as an employee on behalf of your employer or a postgraduate student of an affiliated institution which is paying any applicable article publishing charge ("APC") for Open Access articles. Where the Submitting Author wishes to make the Work available on an Open Access basis (and intends to pay the relevant APC), the terms of reuse of such Open Access shall be governed by a Creative Commons licence – details of these licences and which Creative Commons licence will apply to this Work are set out in our licence referred to above.

Other than as permitted in any relevant BMJ Author's Self Archiving Policies, I confirm this Work has not been accepted for publication elsewhere, is not being considered for publication elsewhere and does not duplicate material already published. I confirm all authors consent to publication of this Work and authorise the granting of this licence.

For peer review only

Prevalence of polypharmacy in pregnancy: a systematic review

Running title: Polypharmacy in Pregnancy

Authors: Astha Anand[1*], Katherine Phillips[1], Anuradhaa Subramanian[1], Siang Ing Lee[1], Zhaonan Wang[1], Rebecca McCowan[3], Utkarsh Agrawal[2], Adeniyi Francis Fagbamigbe[2, 4], Catherine Nelson-Piercy [5], Peter Brocklehurst[1], Christine Damase-Michel[6,7], Maria Loane[8], Krishnarajah Nirantharakumar[1,9], Amaya Azcoaga-Lorenzo[2#] on behalf of the MuM-PreDiCT Group

* First author
\# Senior author

Affiliations

1. Institute of Applied Health Research, University of Birmingham, Birmingham, UK.
2. Division of Population and Behavioural Sciences, School of Medicine, University of St Andrews, UK
3. University of Glasgow College of Medicine, Veterinary & Life Sciences, University of Glasgow, Glasgow, UK.
4. Department of Epidemiology and Medical Statistics, College of Medicine, University of Ibadan, Nigeria
5. Guy's and St. Thomas' NHS Foundation Trust, UK
6. Medical and Clinical Pharmacology, School of Medicine, Université Toulouse III, France
7. INSERM, Center for Epidemiology and Research in Population Health (CERPOP), CIC 1436, France
8. The Institute of Nursing and Health Research, Ulster University, UK
9. Midlands Health Data Research UK, Birmingham, UK.

Orcid ID

Astha Anand 0000-0003-0494-321X, Amaya Azcoaga-Lorenzo 0000-0003-3307-878X, Katherine Phillips 0000-0003-0674-605X , Anuradhaa Subramanian 0000-0001-8875- 7363, Siang Ing Lee 0000-0002-2332-5452, Rebecca McCowan 0000-0002-2379-892X, Utkarsh Agrawal 0000-0001-5181-6120, Adeniyi Francis Fagbamigbe 0000-0001-9184-8258, , , Catherine Nelson-Piercy 0000-0001-9311-1196, Peter Brocklehurst 0000-0002-9950-6751, Christine Damase-Michel 0000-0001-5018-0108, Maria Loane 0000-0002-1206-3637, Helen Dolk 0000-0001-6639-5904, Krishnarajah Nirantharakumar 0000-0002-6816-1279

For peer review only - http://bmjopen.bmj.com/site/about/guidelines.xhtml

Polypharmacy in pregnancy

Corresponding author: Amaya Azcoaga-Lorenzo, Division of Population and Behavioural Sciences, School of Medicine, University of St Andrews, KY16 9AJ, UK. Email: aal22@st-andrews.ac.uk.

Word count: 2945

Abstract

Objectives

The use of medications amongst pregnant women has been rising over the past few decades but the reporting of polypharmacy has been sporadic. The objective of this review is to identify literature reporting the prevalence of polypharmacy amongst pregnant women, the prevalence of multimorbidity in women taking multiple medications in pregnancy, and associated effects on maternal and offspring outcomes.

Design

MEDLINE and Embase were searched from their inception up to 14th September 2021for intervention al trials, observational studies and systematic reviews reporting on the prevalence of polypharmacy or the use of multiple medications in pregnancy were included. Data on prevalence of polypharmacy, prevalence of multimorbidity, combinations of medications and pregnancy and offspring outcomes were extracted. A descriptive analysis was performed.

Results

Fourteen studies met the review criteria. Prevalence of women being prescribed two or more medications during pregnancy ranged from 4.9% (4.3%-5.5%) to 61.3% (61.3%-63.5%), with a median of 22.5%. For the first trimester, prevalence ranged from 4.9% (4.7%- 5.14%) to 33.7% (32.2%-35.1%). No study reported on prevalence of multimorbidity, or associated pregnancy outcomes in women exposed to polypharmacy.

Conclusion

There is a significant burden of polypharmacy amongst pregnant women. There is a need for evidence on the combinations of medications prescribed in pregnancy, how this specifically affects women with multiple long-term conditions and the associated benefits and harms.

Article Summary

Strengths and Limitations of this study

- A structured and substantial review of the literature, according to a pre-planned and comprehensive search.
- Articles screened rigorous inclusion and exclusion criteria.
- As there is no consensus definition, polypharmacy was reported according to a variety of definitions in this review.

For peer review only - http://bmjopen.bmj.com/site/about/guidelines.xhtml

- Due to the methodological limitations of included studies, it could not be determined whether medications were prescribed concurrently or whether medication was complied with, meaning prevalence of polypharmacy may have been over-estimated
- No studies reporting on maternal or offspring outcomes associated with polypharmacy were found

Funding

This work was funded by the Strategic Priority Fund "Tackling multimorbidity at scale" programme [grant number MR/W014432/1]

Keywords

polypharmacy, pregnancy, maternity, epidemiology, multimorbidity, systematic review

For peer review only

Tweetable abstract

Our systematic review shows significant burden of polypharmacy in pregnancy but outcomes for women and offspring are unknown.

For peer review only

Introduction

Medications may be prescribed in pregnancy for the management of pregnancy-related symptoms (such as nausea and vomiting), pre-existing maternal health conditions or pregnancy-related complications (1-3). The use of medications amongst pregnant women has been rising over the past few decades (4-6), which could be attributed to a rise in the prevalence of maternal comorbidities, obesity and, in the UK and other high income countries, a rise in the average maternal age (7, 8). With rising medication use, the use of multiple medications is also likely to increase (3).Whilst many studies have assessed overall medication use amongst pregnant women, fewer studies have focused on polypharmacy.

Polypharmacy is broadly defined as the use of multiple medications by a single patient, but various definitions are found in the literature. A systematic review of definitions of polypharmacy found that studies reported various numerical definitions and some incorporating duration or appropriateness of therapy into their definition (9). As the number of medications taken together increases, medication interactions and adverse events are expected to increase also. The use of multiple medication has been reported amongst specific subpopulation of pregnant women, such as women with psychiatric illness, epilepsy or human immunodeficiency virus (HIV) (10-12). However, the polypharmacy rate amongst general population of pregnant women is not as well understood.

Drug pharmacokinetics are altered in pregnancy due to physiological changes in the expectant mothers(13, 14). However, few clinical trials are undertaken amongst pregnant women due to concerns around maternal and fetal safety (15, 16). Even fewer studies assess the outcome of polypharmacy. It is unclear what the effect of combining medications might be. It is unknown whether these combinations worsen known side effects, result in novel adverse events or indeed have a synergistic or beneficial effect (17). Understanding these effects will allow clinicians and women to make more informed decisions about continuing, starting or stopping medications before and during pregnancy.

The objective of this systematic review was to assess the published literature reporting on the prevalence of polypharmacy amongst pregnant women, the prevalence of multimorbidity in women taking multiple medications in pregnancy, and the effect of multiple medication use on maternal and offspring outcomes.

For peer review only

Methods

A systematic review of the literature was performed in order to identify relevant studies examining the prevalence of polypharmacy in pregnancy, the most common medication combination, rate of multimorbidity and outcomes amongst women exposed to polypharmacy.

Protocol and registration

Protocol for this systematic review has been published on Prospero (Protocol ID CRD42021223966, Available from: https://www.crd.york.ac.uk/prospero/display_record.php?ID=CRD42021223966)(18).

Eligibility Criteria

We included interventional trials, observational studies (cohort studies and case control studies) and systematic reviews reporting the prevalence of polypharmacy or use of multiple medications in pregnant women, where the prevalence of polypharmacy could be extracted from tables or figures. The study authors' definition of polypharmacy was used. Where polypharmacy was not defined by the authors of the individual studies, we defined polypharmacy to mean the use of two or more medications.

Exclusion Criteria

We excluded studies focused on specific subpopulations of pregnant women instead of general prevalence of polypharmacy (such as pregnant women with specific medical conditions, or with high-risk pregnancies), as we were interested in the population-based prevalence. We excluded expert opinions, conference abstract, case report, narrative review, laboratory and animal studies. Studies based on non-pregnant women were excluded and unpublished data were not sought.

We did not exclude non-English papers. For any non-English paper identified, native speaker would extract data where possible. Where this was not possible, two independent reviewers (AA and AAL) extracted the data using an online translation service (Google Translate).

Outcome measurement

The primary outcome was prevalence of polypharmacy, as defined by the authors, or the use of two or more medications, where polypharmacy was not defined by the authors.

We also assessed the prevalence of multimorbidity and maternal or offspring outcomes amongst women exposed to polypharmacy. The individual studies' definition of multimorbidity was used where specified. Where the definition of multimorbidity was not specified by the authors, it was defined as the presence of two or more long term health conditions, including mental health conditions.

Search strategy

MEDLINE was searched for relevant papers from 1946 to 14th September 2021 and Embase was searched from 1974 to 14th September 2021. An experienced librarian helped to

For peer review only - http://bmjopen.bmj.com/site/about/guidelines.xhtml

develop the search strategy. The full search strategy for Embase is provided in Appendix S1.

Study selection and data extraction

Study selection was conducted in two phases. In the first phase, title and abstracts were screened by two independent reviewers against the eligibility criteria (AA screened all papers, SIL, AS, AAF, UA and ZW were the second reviewers). We retrieved full-text papers for all potentially eligible studies. In the second phase, full-text papers were assessed by two authors independently (AA and AAL) against the eligibility criteria. For all eligible studies, two authors (AA and AAL) independently extracted the data using a piloted data extraction form, and assessed the risk of bias. Discrepancies were reviewed and resolved by a third independent reviewer (ZW).

Data items extracted included: purpose of the study, setting, recruitment, inclusion and exclusion criteria, participant demographics (age, ethnicity, parity, deprivation), definition of polypharmacy, prevalence of polypharmacy, classification system for grouping medications, list of health conditions, follow-up length, any secondary outcomes, funding, and conflict of interest.

We used the Newcastle-Ottawa critical appraisal checklist for observational studies to assess risk of bias in the individual studies during the data extraction stage.

Summary measures and results synthesis

Results are presented as descriptive analysis. Primary outcome is presented as proportion or prevalence. We stratified the analysis according to the various definitions of polypharmacy from the primary studies (e.g., 2 or more medications) and the setting (primary or secondary care). Given the heterogenous nature of the studies, statistical pooling and analysis was not possible. PRISMA checklist for reporting of systematic reviews has been followed (Appendix S2).

Patient and Public involvement

Patients were not involved in the development of the research question, study design or selection of outcome measures.

For peer review only

Results

Study Selection

We screened 2,228 titles and abstracts. Of those, 46 papers were subjected to detailed evaluation in full text screening (6, 19-63), and 14 met inclusion criteria (6, 19-31). The main reasons for exclusion were an inadequate method of reporting prevalence of polypharmacy or reporting on specific subpopulation of pregnant women. The results from each step of the review process are documented in a PRISMA flow diagram (Figure 1).

Study Characteristics

Table 1 shows the characteristics of the included studies. Studies were published between 1991 and 2020. The study populations ranged between 369 and 981,392. Six studies examined prevalence of polypharmacy using administrative data, seven used surveys to collect self-reported medication use. One study used administrative data for prescription medications and self-report for the use of over the counter (OTC) medications.

In seven studies, women were recruited from hospitals (either birth hospital or antenatal clinic). (6, 20, 21, 25, 26, 28, 29) In the other seven studies, participants were sampled from a national registry or population-based database (such as pharmacy records). (19, 22-24, 27, 30, 31)

Mitchell et al. reported results from two different cohorts; Birth Defect Study (BDS)(64) and National Birth Defects Prevention Study (NBDPS)(65). BDS included both cases of mothers with children born with birth defects and population-based control and therefore oversampled children born with birth defect and was not representative of the general population. For the NBDPS cohort, Mitchell et al reported results for population-based controls only and so results for polypharmacy use for Mitchell et al (shown in figures 1-3), reflect the NBDPS study only. (26)

For peer review only

For peer review only

For peer review only - http://bmjopen.bmj.com/site/about/guidelines.xhtml

Table 1 – List of included studies and study characteristics

Author	Study Design	Country/ Location	Inclusion criteria	Source (administrative data/self-reported)	Total number of pregnancies	Trimester studied	Polypharmacy definition	Definition of polypharmacy used	Prevalence reported
Haas et al (2018) (6)	Prospective longitudinal cohort study	USA	Primiparous women, aged 13 or over, in the first trimester	Self-report	9546	Across the three trimesters	≥ 5 medications during the same epoch	≥ 5 (as defined by the authors)	13%
van Gelder et al (2014) (19)	Retrospective cohort study	Netherlands	Female person (15-50 years older than child) at the same address as child aged 0 -5 years, with no other female at the address	Administrative record	32016	First trimester	Polypharmacy not defined by author	≥ 2	4.90%
Refuerzo et al (2005) (20)	Prospective observational	USA	Women who gave birth at a single, university-based, tertiary-care hospital	Self-report	418	Across the three trimesters	Polypharmacy not defined by author	≥ 2	33.50%
Gomes et al (1999) (21)	Retrospective survey	Brazil	Pregnant women who gave birth in one of 5 participating hospitals	Self-report	1620	Across the three trimesters	Polypharmacy not defined by author	>6	24.90%
Tinker et al (2016) (22)	Cross-sectional surveys	USA	Non-institutionalised civilian women aged 15-44	Self-report	1350	Prior 30 days (Pregnancies across three trimesters)	Polypharmacy not defined by author	≥ 2	6.10%
Malm et al (2004) (23)	A retrospective, register-based cohort study	Finland	All women who applied for maternal grants in 1999 and the mother has visited a maternity clinic before the end of the fourth month	Administrative record	43470	Across the three trimesters	Polypharmacy not defined by author	≥ 10	0.20%
Ingstrup et al (2018) (24)	Population based-descriptive study	Denmark	Pregnancies ending in live-born singletons during 1997-2012 to women aged between 15-55	Administrative record	981392	Across the three trimesters	Polypharmacy not defined by author	≥ 2	42.74%
Cleary et al (2010) (25)	Retrospective cohort	Ireland	Pregnancy booking and midwife care at tertiary level hospital	Self-report	61252	Early pregnancy (first trimester)	Polypharmacy not defined by author	≥ 2	29.40%
Mitchell et al (2011) (NBDPS	Cross-sectional study	USA and Canada	NBDPS study- controls were randomly selected from birth certificates or from birth hospitals	Self-report	5008	Across the three trimesters	Polypharmacy not defined by author	≥ 4	4.90%

For peer review only - http://bmjopen.bmj.com/site/about/guidelines.xhtml

Polypharmacy in pregnancy

Study Arm Reported) (26)									
Zhang et al. (2019) (27)	Retrospective cohort	China	Singleton deliveries, mothers aged between 12 to 54	Administrative data	7946 (2896 pregnancies covering all three trimesters)	Across the three trimesters	Polypharmacy not defined by author	≥ 2	9.19%
Buitendijk et al (1991) (28)	Retrospective survey	USA	All women who made their first prenatal visit to private obstetric or midwifery practice, a health maintenance organization, or a hospital clinic and were scheduled for delivery at Yale-New Haven Hospital	Self-report	4186	Early pregnancy (first trimester)	Polypharmacy not defined by author	≥ 2	33.70%
Obadeji et al (2020) (29)	Cross sectional study	Nigeria	All consecutive consenting women who came for outpatient antenatal care at a secondary health care facility	Administrative data for prescription drug and self-report for OTC	369	Cross-sectional (Pregnancies across three trimesters)	Polypharmacy not defined by author	≥ 3	38.30%
Olesen et al (1998) (30)	Retrospective cohort	Denmark	Primiparous women identified through Danish National Birth Registry	Administrative data	16001	Across the three trimesters	More than 3 medications	≥ 4 (as defined by the authors)	2.70%
Schirm et al (2004) (31)	Cross-sectional study	Netherlands	Female person (15-50 years older than child) at the same address as child aged 0 -5 years, with no other female at the address	Administrative data	7500	Across the three trimesters	Polypharmacy not defined by author	≥ 2	62.41%

For peer review only - http://bmjopen.bmj.com/site/about/guidelines.xhtml

Risk of bias within studies

Most of the study cohorts were considered representative of the population they were sampling from. Most studies ascertained pregnancy status using hospital or pharmacy records or from birth registries, which were considered likely to be accurate. Schirm et al and Van Gelder et al used a pharmacy database to identify all children born within a given timeframe. Women of reproductive age living at the same address as the child were identified in the database and their prescription data was collected for the 270 days before the child's date of birth. There is a chance that women could have been misclassified as pregnant if the child was not living with their biological mother (19, 31).

As discussed above, seven studies relied solely on self-reported medication use to measure outcomes, introducing the potential for recall bias (6, 20, 21, 25, 26, 28, 29). The follow-up period was considered adequate for each study. Nine studies reported multiple medication use across the entire pregnancy (Figure 4) (6, 20, 21, 23, 24, 26, 27, 30, 31), while three studies reported for early pregnancy (first trimester) only (19, 25, 28). Obadeji et al and Tinker et al employed a cross-sectional design and included women across all trimesters. (22, 29) Follow-up rates were considered adequate for all studies, with no study having significant numbers of subjects lost to follow up. Table S1 shows the outcome of the risk of bias assessment.

Prevalence of polypharmacy

The prevalence of polypharmacy-ranged from 0.2% - 62.4%, with a median value of 12.3%. Exclusion of over-the-counter drugs does not change the spread of the prevalence of polypharmacy (Figure 3).

Prevalence by polypharmacy definition

The prevalence of polypharmacy, defined as the use or 2 or more medications, ranged from 4.9% (4.3%-5.5%) to 61.3% (61.3%-63.5%) based on eight papers, with a median value of 22.5% (19, 20, 22, 24, 25, 27, 28, 31) (Figure 2). Only two studies explicitly defined polypharmacy. Olesen et al. defined it as the use of four or more medications (prevalence 2.7%) and Haas et al. defined it as the use of five or more medications (prevalence 13%) (6, 30).

Other studies did not define polypharmacy, but stratified results by the number of medications taken (Figure 2). Mitchell et al and Gomes et al did not define polypharmacy and only reported the use of four or more medications (15.7%) and six or more drugs (24.9%), respectively. (21, 26) Malm et al (2004) reported that 0.2% of women purchased ten or more different medications during the whole period of pregnancy. (23) Due to heterogeneity within the data, meta-analysis was not undertaken.

Prevalence of Polypharmacy by Trimester breakdown

Two studies, Obadeji et al and Zhang et al, reported polypharmacy use across the whole pregnancy and also subdivided into trimesters. For these two studies, polypharmacy prevalence across the whole pregnancy has been summarised. (27, 29) Obadeji et al reported a prevalence of 50.0% (95% CI 21.1%-79.0%) in the first trimester compared to a prevalence of 39.8% (95% CI 34.8%-44.8%) across all three trimesters. Zhang et al reported a

For peer review only - http://bmjopen.bmj.com/site/about/guidelines.xhtml

prevalence of 3.8%% (95% CI 3.1%-4.6%) in the first trimester compared to a prevalence of 9.2% (95% CI 8.3%-10.2%) across all three trimesters.

Due to the design and nature of the study, Van Gelder et al, Cleary et al and Buitendijk et al have reported medication use during early pregnancy or the first trimester period only, reporting polypharmacy prevalence of 4.9% (95% CI 4.7%-5.1%), 11.5% (95% CI 11.3%-11.8%) and 33.7% (95% CI 32.2%-35.1%). (19, 28) In a cross-sectional study, Tinker et al. cover medication use in the last 30 days only but across the whole pregnancy. (22) Olesen et al cover a period from 12 weeks prenatal to 12 weeks postpartum in the analysis. (30) Figure 4 shows polypharmacy prevalence when including studies which covered the entire duration of pregnancy.

Prevalence of polypharmacy by Medications included

Whilst most of the studies reported any possible medication use, van Gelder et al report only the teratogenic medications used and not all possible medications. (19)

Eight studies include over-the-counter medications in their analysis – results for polypharmacy prevalence, subdivided by inclusion of over-the-counter drugs, are shown in Figure 3. (6, 20, 21, 25-29) Reported prevalence of polypharmacy for studies that included OTC medications ranged from 4.9% (Mitchell et al (95% CI 4.3%-5.5%)) to 38.3% (Obadeji et al (95% CI 33.3%-43.3%)). Reported prevalence of polypharmacy for studies that excluded OTC medications ranged from 0.2% (Malm et al (95% CI 0.2%-0.2%) to 62.4% (Schirm et al (61.3%-63.5%)). Of note, Malm et al include some but not all OTC medications, as some medications were reimbursable and therefore were included in the national medication prescription register used for the study. (23)

Five studies specifically excluded vitamins and minerals (such as folic acid and iron) from the study design. (19, 21, 22, 28, 30) The definition of routine prenatal vitamins or minerals was determined by the authors of the original studies. Haas et al analysed medication use, when vitamins and minerals were included and excluded. When including vitamins and minerals, Haas et al report 30.5% (95% CI 29.6%-31.5%) of women use 5 or more medication; whereas, only 13% (95% CI 12.3%-13.7%) use 5 or more medications if vitamins and minerals are excluded, as shown in Figure 3 (6)

Eight studies described the different medications used or prescribed to pregnant women. (19, 20, 24-26, 28, 29, 31) However, none specified which medications were used in combination or were used by women exposed to polypharmacy.

Multimorbidity and maternal or offspring outcomes

No studies were found describing which conditions women who were exposed to polypharmacy were treated for, and none specify how many women had multimorbidity or long-term illness. No studies were found which reported on maternal or offspring outcomes.

For peer review only - http://bmjopen.bmj.com/site/about/guidelines.xhtml

Discussion

Main findings

Studies of multiple medication use in pregnancy reported a wide range in the prevalence of polypharmacy. Where the definition of polypharmacy was two or more medications only, the prevalence of polypharmacy ranged from 5%-62%. However, the definition of polypharmacy was varied, and most studies were not considered truly representative of all pregnant women.

Strengths and limitations

This systematic review has several important strengths. We developed a structured and substantial review of the literature, according to pre-planned and comprehensive search terms with the help of an experienced librarian. Screening was conducted according to a rigorous inclusion and exclusion criteria, and we used two independent reviewers for data extraction to minimise bias. Two databases were searched: MEDLINE and Embase. We did not limit our search to studies published in the English language to minimise language bias, although specific databases in languages other than English were not included.

There are limited studies specifically assessing polypharmacy in pregnancy are limited. There is no consensus on the definition of polypharmacy and polypharmacy is often not explicitly defined in the studies. Where polypharmacy is defined, the definition varies from study to study. Only two studies in this systematic review subdivide polypharmacy use in different trimesters. Exclusion of routine prenatal vitamins is often determined by individual authors. Inclusion of OTC medications is variable and often determined by the data available.

The main caveat from these studies is that it is not clear whether use of multiple medication in pregnancy was simultaneous or sequential. Additionally, prescription and dispensation of medications do not equate to compliance. Qualitative studies show that women are less likely to use medications when pregnant, especially if potential risks to the fetus and benefits to the mother have not been adequately communicated (64).

In majority of the studies identified in this systematic review, pregnancy was confirmed retrospectively or identified using birth records. Thus, not all pregnancies were captured and pregnancies resulting in terminations, miscarriages or still birth, were excluded. These pregnancy outcomes are clinically important and the use of multiple medications in these groups warrants further assessment.

Whilst some of the studies outline common medications used by pregnant women overall, none of the studies describe the combinations of medications used in pregnancy. Pregnant women have been described as drug orphans, as they are often excluded from clinical trials. The maternal and offspring outcomes following medication exposure during pregnancy are often determined through retrospective observational studies (15, 16). Pregnancy outcomes of a few common combinations of medications in specific groups of pregnant women are known (12) however, none of the studies assessing polypharmacy in this systematic review evaluate the effect of taking multiple medication for the women and their offspring.

For peer review only - http://bmjopen.bmj.com/site/about/guidelines.xhtml

Interpretation

The finding of 5-62% of pregnant women taking two or more medications is in keeping with a previous systematic review of the literature evaluating individual-level exposures to prescription medications in pregnancy. This review, which included only studies from developed (OECD) countries, found 27-93% of women filled at least one prescription during pregnancy (65).

The findings of this review should be interpreted with caution. As discussed above, the literature is not necessarily representative of the general pregnant population, inclusion of certain medications was variable and, where polypharmacy was defined, there were differences in the definitions used. This variation is in keeping with the findings of a systematic review of definitions of polypharmacy in older people (9). This review also found that, in some instances, safety and appropriateness of medications were taken into account when defining polypharmacy. This is an important consideration in pregnancy, although, as discussed, there is often not adequate safety information available.

Despite this, the median value of one in five women taking two or more medications, indicates that a significant proportion of women are potentially exposed to multiple medication in pregnancy. The lack of studies into combinations of medications taken during pregnancy and the effects of polypharmacy on maternal and offspring outcomes highlights the urgent need for further research in this area.

Conclusion

The reported prevalence of polypharmacy amongst pregnant women is variable, depending on which medications were included. Commonly, only pregnancies resulting in live birth are reported in studies assessing polypharmacy. This systematic review shows relatively large burden of polypharmacy amongst pregnant women and highlights the need to evaluate the outcomes for these women and for their offspring. This is especially relevant for women with multiple, long-term conditions, who are more likely to need multiple medications.

Figures

Figure 1. 2020 PRISMA flow diagram
Figure 2. Forest plot showing prevalence of polypharmacy, subdivided by the definition of polypharmacy (number of medications taken)
Figure 3. Forest plot showing prevalence of polypharmacy, subdivided by inclusion or exclusion of over-the-counter medications
Figure 4. Forest plot showing prevalence of polypharmacy (as defined by the study), for studies which covered all trimesters of the pregnancy and the first trimester

Supporting Information

Table S1. Newcastle-Ottawa Quality Assessment Scale
Appendix S1. Search strategy.
Appendix S2. Prisma checklist.

For peer review only - http://bmjopen.bmj.com/site/about/guidelines.xhtml

Statements and Declarations

Funding

This work was funded by the Strategic Priority Fund “Tackling multimorbidity at scale” programme [grant number MR/W014432/1] delivered by the Medical Research Council and the National Institute for Health Research in partnership with the Economic and Social Research Council and in collaboration with the Engineering and Physical Sciences Research Council.

Competing Interests

None declared

For peer review only

Acknowledgements

The authors thank Ms. Vicki Cormie, Senior Librarian (Academic Liaison) at University of St. Andrews for help with the search strategy. The authors thank Professor Helen Dolk for advice and comments on the manuscript.

Author Contributions

KN, AA and AAL conceived the study and AA, KN and AAL designed the protocol. AA and AAL performed the literature search. AA, ZW, AS, SIL, UA, AAF, RM and AAL selected the studies and extracted the relevant information. AA synthesised the data and wrote the first draft of the paper. AA, KP, SIL, AS, RM, CNP, PB, CDM, HD, ML, KN and AAL critically revised successive drafts of the paper. AA is the guarantor of the review.

Details of ethics approval

None required

Data Sharing Statement

No additional data are available

Polypharmacy in pregnancy

For peer review only

For peer review only - http://bmjopen.bmj.com/site/about/guidelines.xhtml

References

1. Kulkarni J, Worsley R, Gilbert H, Gavrilidis E, Van Rheenen TE, Wang W, et al. A prospective cohort study of antipsychotic medications in pregnancy: The first 147 pregnancies and 100 one year old babies. PLoS ONE. 2014;9(5):e94788.
2. Beeson JG, Homer CSE, Morgan C, Menendez C. Multiple morbidities in pregnancy: Time for research, innovation, and action. PLOS Medicine. 2018;15(9):e1002665.
3. Barnett K, Mercer SW, Norbury M, Watt G, Wyke S, Guthrie B. Epidemiology of multimorbidity and implications for health care, research, and medical education: a cross-sectional study. Lancet. 2012;380(9836):37-43.
4. Mitchell AA, Gilboa SM, Werler MM, Kelley KE, Louik C, Hernandez-Diaz S. Medication use during pregnancy, with particular focus on prescription drugs: 1976-2008. American Journal of Obstetrics and Gynecology. 2011;205(1):51.
5. Headley J, Northstone K, Simmons H, Golding J. Medication use during pregnancy: data from the Avon Longitudinal Study of Parents and Children. Eur J Clin Pharmacol. 2004;60(5):355-61.
6. Haas DM, Marsh DJ, Dang DT, Parker CB, Wing DA, Simhan HN, et al. Prescription and Other Medication Use in Pregnancy. Obstetrics and gynecology. 2018;131(5):789-98.
7. Statistics OfN. Birth characteristics in England and Wales: 2019 ons.gov.uk: Office for National Statistics; 2019 [Available from: https://www.ons.gov.uk/peoplepopulationandcommunity/birthsdeathsandmarriages/livebirths/bulletins/birthcharacteristicsinenglandandwales/2019.
8. MuM-PreDiCT. Epidemiology of pre-existing multimorbidity in pregnant women in the UK in 2018: a cross sectional study using CPRD, SAIL and SMR 2021 [Available from: https://docs.google.com/document/d/1mZf9YSqCIZIX8Og2ROy9epgloYQlabtq/edit.
9. Masnoon N, Shakib S, Kalisch-Ellett L, Caughey GE. What is polypharmacy? A systematic review of definitions. BMC Geriatrics. 2017;17(1):230.
10. Okoli C, Schwenk A, Radford M, Myland M, Taylor S, Darley A, et al. Polypharmacy and potential drug-drug interactions for people with HIV in the UK from the Climate-HIV database. HIV Med. 2020;21(8):471-80.
11. Kinney MO, Morrow J. Epilepsy in pregnancy. BMJ. 2016;353:i2880.
12. Peindl KS, Masand P, Mannelli P, Narasimhan M, Patkar A. Polypharmacy in pregnant women with major psychiatric illness: a pilot study. J Psychiatr Pract. 2007;13(6):385-92.
13. Feghali M, Venkataramanan R, Caritis S. Pharmacokinetics of drugs in pregnancy. Semin Perinatol. 2015;39(7):512-9.
14. Pariente G, Leibson T, Carls A, Adams-Webber T, Ito S, Koren G. Pregnancy-Associated Changes in Pharmacokinetics: A Systematic Review. PLoS medicine. 2016;13(11):e1002160-e.
15. Scaffidi J, Mol BW, Keelan JA. The pregnant women as a drug orphan: a global survey of registered clinical trials of pharmacological interventions in pregnancy. Bjog. 2017;124(1):132-40.
16. Illamola SM, Bucci-Rechtweg C, Costantine MM, Tsilou E, Sherwin CM, Zajicek A. Inclusion of pregnant and breastfeeding women in research - efforts and initiatives. Br J Clin Pharmacol. 2018;84(2):215-22.
17. Guthrie B, Makubate B, Hernandez-Santiago V, Dreischulte T. The rising tide of polypharmacy and drug-drug interactions: population database analysis 1995–2010. BMC Medicine. 2015;13(1):74.

For peer review only - http://bmjopen.bmj.com/site/about/guidelines.xhtml

18. Astha Anand AS, Siang Lee, Krishnarajah Nirantharakumar, Amaya Azcoaga-Lorenzo. Prevalence of polypharmacy in pregnancy and associated health outcomes in mothers and offspring crd.york.ac.uk: Propero (National Institute for Health Research); 2021 [Available from: https://www.crd.york.ac.uk/prospero/display_record.php?ID=CRD42021223966).
19. Van Gelder MMHJ, Bos JHJ, Roeleveld N, De Jong-Van Den Berg LTW. Drugs associated with teratogenic mechanisms. Part I: Dispensing rates among pregnant women in the Netherlands, 1998-2009. Human Reproduction. 2014;29(1):161-7.
20. Refuerzo JS, Blackwell SC, Sokol RJ, Lajeunesse L, Firchau K, Kruger M, et al. Use of over-the-counter medications and herbal remedies in pregnancy. American journal of perinatology. 2005;22(6):321-4.
21. Gomes KR, Moron AF, Silva R, Siqueira AA. Prevalence of use of medicines during pregnancy and its relationship to maternal factors. Revista de saude publica. 1999;33(3):246-54.
22. Tinker SC, Broussard CS, Frey MT, Gilboa SM. Prevalence of prescription medication use among non-pregnant women of childbearing age and pregnant women in the United States: NHANES, 1999-2006. Maternal and child health journal. 2015;19(5):1097-106.
23. Malm H, Martikainen J, Klaukka T, Neuvonen PJ. Prescription of hazardous drugs during pregnancy. Drug safety. 2004;27(12):899-908.
24. Ingstrup KG, Liu X, Gasse C, Debost JCP, Munk-Olsen T. Prescription drug use in pregnancy and variations according to prior psychiatric history. Pharmacoepidemiology and Drug Safety. 2018;27(1):105-13.
25. Cleary BJ, Butt H, Strawbridge JD, Gallagher PJ, Fahey T, Murphy DJ. Medication use in early pregnancy-prevalence and determinants of use in a prospective cohort of women. Pharmacoepidemiology and drug safety. 2010;19(4):408-17.
26. Mitchell AA, Gilboa SM, Werler MM, Kelley KE, Louik C, Hernandez-Diaz S, et al. Medication use during pregnancy, with particular focus on prescription drugs: 1976-2008. American journal of obstetrics and gynecology. 2011;205(1):51.e1-8.
27. Zhang J, Ung COL, Wagner AK, Guan X, Shi L. Medication use during pregnancy in mainland china: A cross-sectional analysis of a national health insurance database. Clinical Epidemiology. 2019;11:1057-65.
28. Buitendijk S, Bracken MB. Medication in early pregnancy: prevalence of use and relationship to maternal characteristics. American journal of obstetrics and gynecology. 1991;165(1):33-40.
29. Obadeji ST, Obadeji A, Bamidele JO, Ajayi FT. Medication use among pregnant women at a secondary health institution: Utilisation patterns and predictors of quantity. African Health Sciences. 2020;20(3):1206-16.
30. Olesen C, Steffensen FH, Nielsen GL, De Jong-Van Den Berg L, Olsen J, Sorensen HT, et al. Drug use in first pregnancy and lactation: A population-based survey among Danish women. European Journal of Clinical Pharmacology. 1999;55(2):139-44.
31. Schirm E, Meijer WM, Tobi H, de Jong-van den Berg LTW. Drug use by pregnant women and comparable non-pregnant women in The Netherlands with reference to the Australian classification system. European journal of obstetrics, gynecology, and reproductive biology. 2004;114(2):182-8.
32. Alani AHHDA, Suhaimi AM, Hassan BAR, Mohammed AH. Use, awareness, knowledge and beliefs of medication during pregnancy in Malaysia. Osong Public Health and Research Perspectives. 2021;11(6):373-9.
33. Zaki NM, Albarraq AA. Use, attitudes and knowledge of medications among pregnant women: A Saudi study. Saudi Pharmaceutical Journal. 2014;22(5):419-28.

For peer review only - http://bmjopen.bmj.com/site/about/guidelines.xhtml

34. Handal M, Engeland A, Ronning M, Skurtveit S, Furu K. Use of prescribed opioid analgesics and co-medication with benzodiazepines in women before, during, and after pregnancy: a population-based cohort study. European journal of clinical pharmacology. 2011;67(9):953-60.
35. Nordeng H, Bayne K, Havnen GC, Paulsen BS. Use of herbal drugs during pregnancy among 600 Norwegian women in relation to concurrent use of conventional drugs and pregnancy outcome. Complementary therapies in clinical practice. 2011;17(3):147-51.
36. Hanley GE, Park M, Oberlander TF. Socieconomic status and psychotropic medicine use during pregnancy: a population-based study in British Columbia, Canada. Archives of Women's Mental Health. 2020;23(5):689-97.
37. Truong BT, Lupattelli A, Kristensen P, Nordeng H. Sick leave and medication use in pregnancy: A European web-based study. BMJ Open. 2017;7(8):e014934.
38. Rouamba T, Valea I, Bognini JD, Kpoda H, Mens PF, Gomes MF, et al. Safety Profile of Drug Use During Pregnancy at Peripheral Health Centres in Burkina Faso: A Prospective Observational Cohort Study. Drugs - Real World Outcomes. 2018;5(3):193-206.
39. Zhang J, Ung COL, Guan X, Shi L. Safety of medication use during pregnancy in mainland China: Based on a national health insurance database in 2015. BMC Pregnancy and Childbirth. 2019;19(1):459.
40. Berard A, Sheehy O. Quebec pregnancy cohort: Prevalence of medication use during gestation and pregnancy outcomes. Therapie. 2014;69(1):71-81.
41. Farooq MO, Reddy SK, Raghu Prasada MS, Nagraj SA, Karupakula S, Natarajan DK. Prescription pattern of the drugs among pregnant inpatients in tertiary care hospital. Journal of Pharmacy Research. 2014;8(7):981-5.
42. Rathod AM, Rathod RM, Jha RK, Gupta VK, Tabish A, Diptendu S. Prescribing trends in antenatal care at a tertiary level teaching hospital of Vidarbha region. Research Journal of Pharmaceutical, Biological and Chemical Sciences. 2012;3(3):865-72.
43. Agarwal M, Nayeem M, Safhi MM, Gupta N, Makeen HA, Sumaily JM. Prescribing pattern of drugs in the department of obstetrics and gynecology in expected mothers in Jazan Region, KSA. International Journal of Pharmacy and Pharmaceutical Sciences. 2014;6(1):658-61.
44. Makiabadi F, Rajeswari R, Jayashree AK. Prescribing Pattern of Drugs in Department of Obstetrics and Gynecology at A Tertiary Care Teaching Hospital, Bangalore, India. Pakistan Journal of Medical and Health Sciences. 2021;15(5):1265-9.
45. Vafai Y, Yeung EH, Hinkle SN, Grewal J, Zhang C, Grantz KL, et al. Prenatal medication use in a prospective pregnancy cohort by pre-pregnancy obesity status. Journal of Maternal-Fetal and Neonatal Medicine. 2021.
46. Sripada R, Suresh Kumar SV, Devanna N, Kandula RR. Pattern of possible drug-drug interactions among different specialties at an indian tertiary care teaching hospital. International Journal of Research in Pharmaceutical Sciences. 2020;11(3):3988-92.
47. Gharoro EP, Igbafe AA. Pattern of drug use amongst antenatal patients in Benin City, Nigeria. Medical science monitor : international medical journal of experimental and clinical research. 2000;6(1):84-7.
48. Lee E, Maneno MK, Smith L, Weiss SR, Zuckerman IH, Wutoh AK, et al. National patterns of medication use during pregnancy. Pharmacoepidemiology and drug safety. 2006;15(8):537-45.
49. Palmsten K, Hernandez-Diaz S, Chambers CD, Mogun H, Lai S, Gilmer TP, et al. The most commonly dispensed prescription medications among pregnant women enrolled in the U.S. Medicaid Program. Obstetrics and Gynecology. 2015;126(3):465-73.
50. Havard A, Barbieri S, Hanly M, Perez-Concha O, Tran DT, Kennedy D, et al. Medications used disproportionately during pregnancy: Priorities for research on the risks

For peer review only - http://bmjopen.bmj.com/site/about/guidelines.xhtml

and benefits of medications when used during pregnancy. Pharmacoepidemiology and Drug Safety. 2021;30(1):53-64.
51. Glavind J, Greve T, de Wolff MG, Hansen MK, Henriksen TB. Medication used in Denmark in the latent phase of labor - Do we know what we are doing? Sexual and Reproductive Healthcare. 2020;25:100515.
52. De Jonge L, Zetstra-Van Der Woude PA, Bos HJ, De Jong-Van Den Berg LTW, Bakker MK. Identifying associations between maternal medication use and birth defects using a case-population approach: An exploratory study on signal detection. Drug Safety. 2013;36(11):1069-78.
53. Ilic M, Lupattelli A, Nordeng H. Medical care contact for infertility and related medication use during pregnancy - a european, cross-sectional web-based study. Norsk Epidemiologi. 2021;29(1-2):97-106.
54. Baraka MA, Steurbaut S, Coomans D, Dupont AG. Ethnic differences in drug utilization pattern during pregnancy: A cross-sectional study. Journal of Maternal-Fetal and Neonatal Medicine. 2013;26(9):900-7.
55. Irvine L, Flynn RWV, Libby G, Crombie IK, Evans JMM. Drugs dispensed in primary care during pregnancy: a record-linkage analysis in Tayside, Scotland. Drug safety. 2010;33(7):593-604.
56. Araujo M, Hurault-Delarue C, Sommet A, Damase-Michel C, Benevent J, Lacroix I. Drug prescriptions in French pregnant women between 2015 and 2016: A study in the EGB database. Therapies. 2020.
57. Girit N, Tugrul I, Demirci B, Bozkurt O, Dost T, Birincioglu M, et al. Drug exposure in early pregnancy might be related to the effects of increased maternal progesterone in implantation period. Journal of Psychosomatic Obstetrics and Gynecology. 2018;39(1):7-10.
58. Bornhauser C, Quack Lotscher Katharina C, Seifert B, Simoes-Wust AP. Diet, medication use and drug intake during pregnancy: Data from the consecutive swiss health surveys of 2007 and 2012. Swiss Medical Weekly. 2017;147(51-52):w14572.
59. Galappatthy P, Ranasinghe P, Liyanage CK, Wijayabandara M, Warapitiya DS, Jayakody RL, et al. Core prescribing indicators and the most commonly prescribed medicines in a tertiary health care setting in a developing country. Advances in Pharmacological and Pharmaceutical Sciences. 2021;2021:6625377.
60. Merlob P, Stahl B, Kaplan B. Children born to mothers using multiple drug therapy during their pregnancy. International Journal of Risk and Safety in Medicine. 1996;8(3):237-41.
61. Eze UI, Eferakeya AE, Oparah AC, Enato EF. Assessment of prescription profile of pregnant women visiting antenatal clinics. Pharmacy Practice. 2007;5(3):135-9.
62. Belay M, Kahaliw W, Ergetie Z. Assessment of drug utilization pattern during pregnancy in adama riferral hospital, oromia region, ethiopia. International Journal of Pharmaceutical Sciences and Research. 2013;4(5):1905-11.
63. Van Gelder MMHJ, Vorstenbosch S, Te Winkel B, Van Puijenbroek EP, Roeleveld N. Using Web-Based Questionnaires to Assess Medication Use during Pregnancy: A Validation Study in 2 Prospectively Enrolled Cohorts. American Journal of Epidemiology. 2018;187(2):326-36.
64. Lynch MM, Amoozegar JB, McClure EM, Squiers LB, Broussard CS, Lind JN, et al. Improving Safe Use of Medications During Pregnancy: The Roles of Patients, Physicians, and Pharmacists. Qual Health Res. 2017;27(13):2071-80.
65. Daw JR, Hanley GE, Greyson DL, Morgan SG. Prescription drug use during pregnancy in developed countries: a systematic review. Pharmacoepidemiol Drug Saf. 2011;20(9):895-902.

For peer review only - http://bmjopen.bmj.com/site/about/guidelines.xhtml

For peer review only

For peer review only - http://bmjopen.bmj.com/site/about/guidelines.xhtml

Polypharmacy in pregnancy

For peer review only - http://bmjopen.bmj.com/site/about/guidelines.xhtml

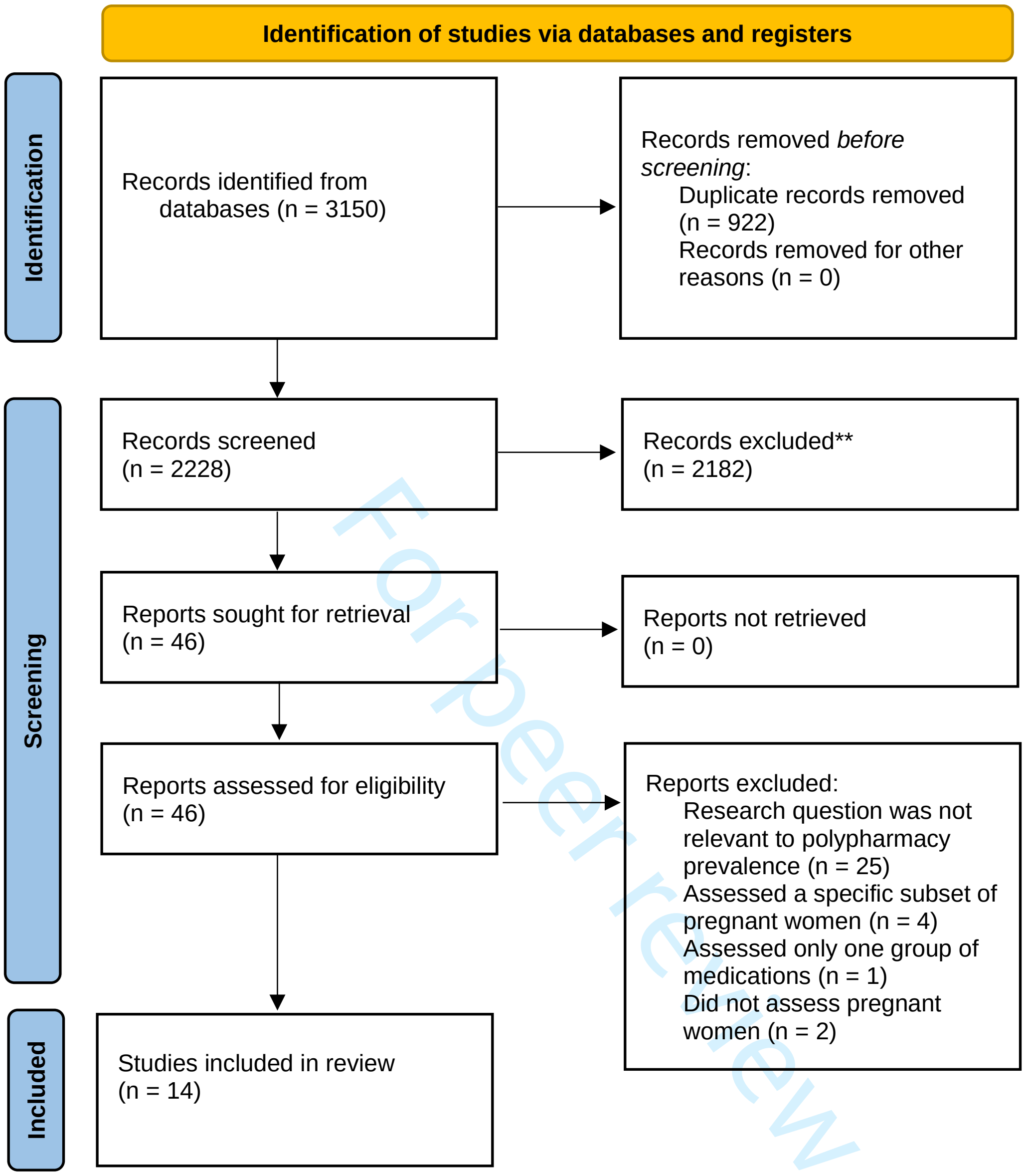

.

From: Page MJ, McKenzie JE, Bossuyt PM, Boutron I, Hoffmann TC, Mulrow CD, et al. The PRISMA 2020 statement: an updated guideline for reporting systematic reviews. BMJ 2021;372:n71. doi: 10.1136/bmj.n71

For peer review only

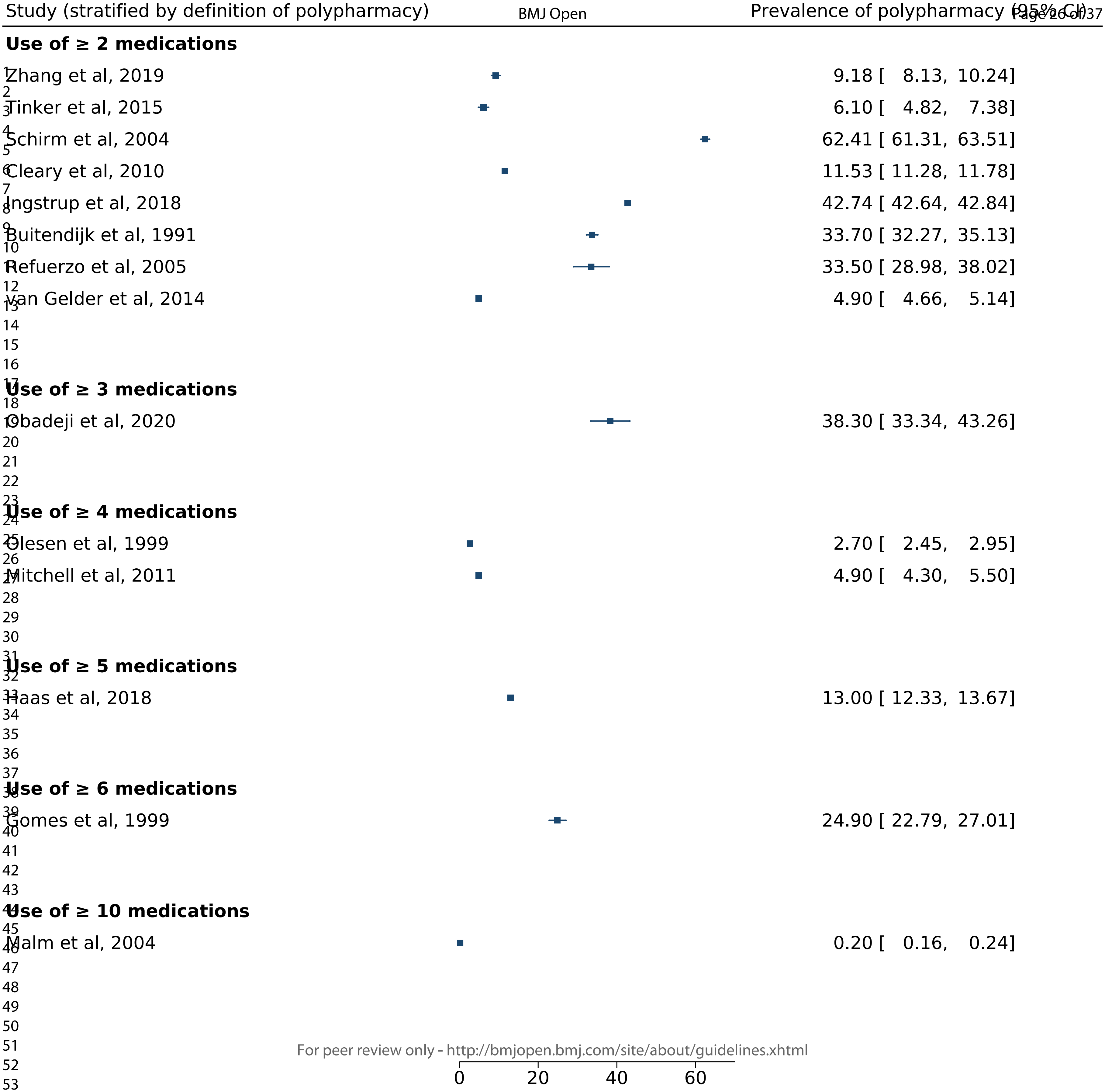
Study (stratified by definition of polypharmacy)
Prevalence of polypharmacy (95% CI)
Use of ≥ 2 medications
Zhang et al, 2019 9.18 [8.13, 10.24]
Tinker et al, 2015 6.10 [4.82, 7.38]
Schirm et al, 2004 62.41 [61.31, 63.51]
Cleary et al, 2010 11.53 [11.28, 11.78]
Ingstrup et al, 2018 42.74 [42.64, 42.84]
Buitendijk et al, 1991 33.70 [32.27, 35.13]
Refuerzo et al, 2005 33.50 [28.98, 38.02]
van Gelder et al, 2014 4.90 [4.66, 5.14]
Use of ≥ 3 medications
Obadeji et al, 2020 38.30 [33.34, 43.26]
Use of ≥ 4 medications
Olesen et al, 1999 2.70 [2.45, 2.95]
Mitchell et al, 2011 4.90 [4.30, 5.50]
Use of ≥ 5 medications
Haas et al, 2018 13.00 [12.33, 13.67]
Use of ≥ 6 medications
Gomes et al, 1999 24.90 [22.79, 27.01]
Use of ≥ 10 medications
Malm et al, 2004 0.20 [0.16, 0.24]
0
20
40
60

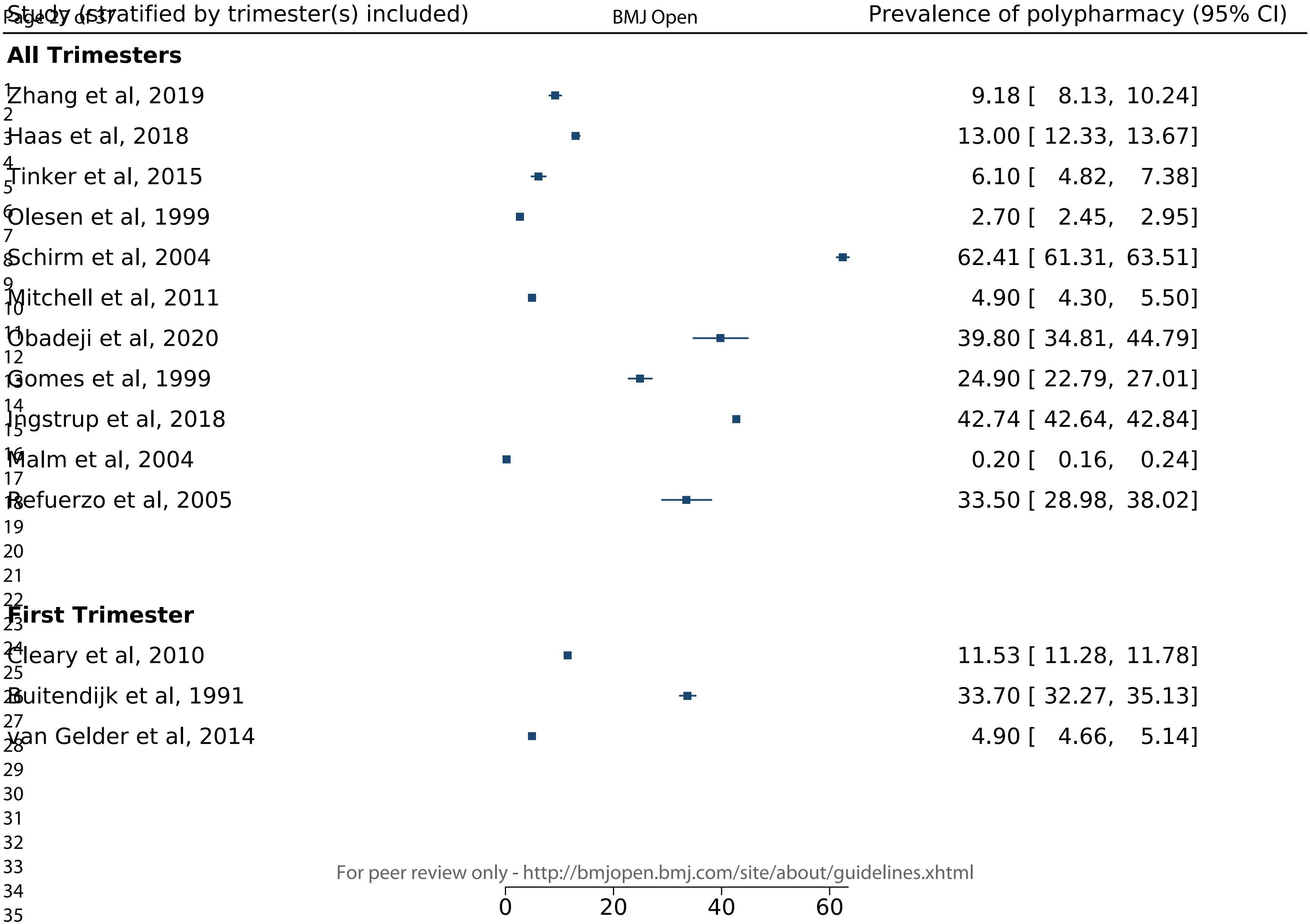

For peer review only - http://bmjopen.bmj.com/site/about/guidelines.xhtml

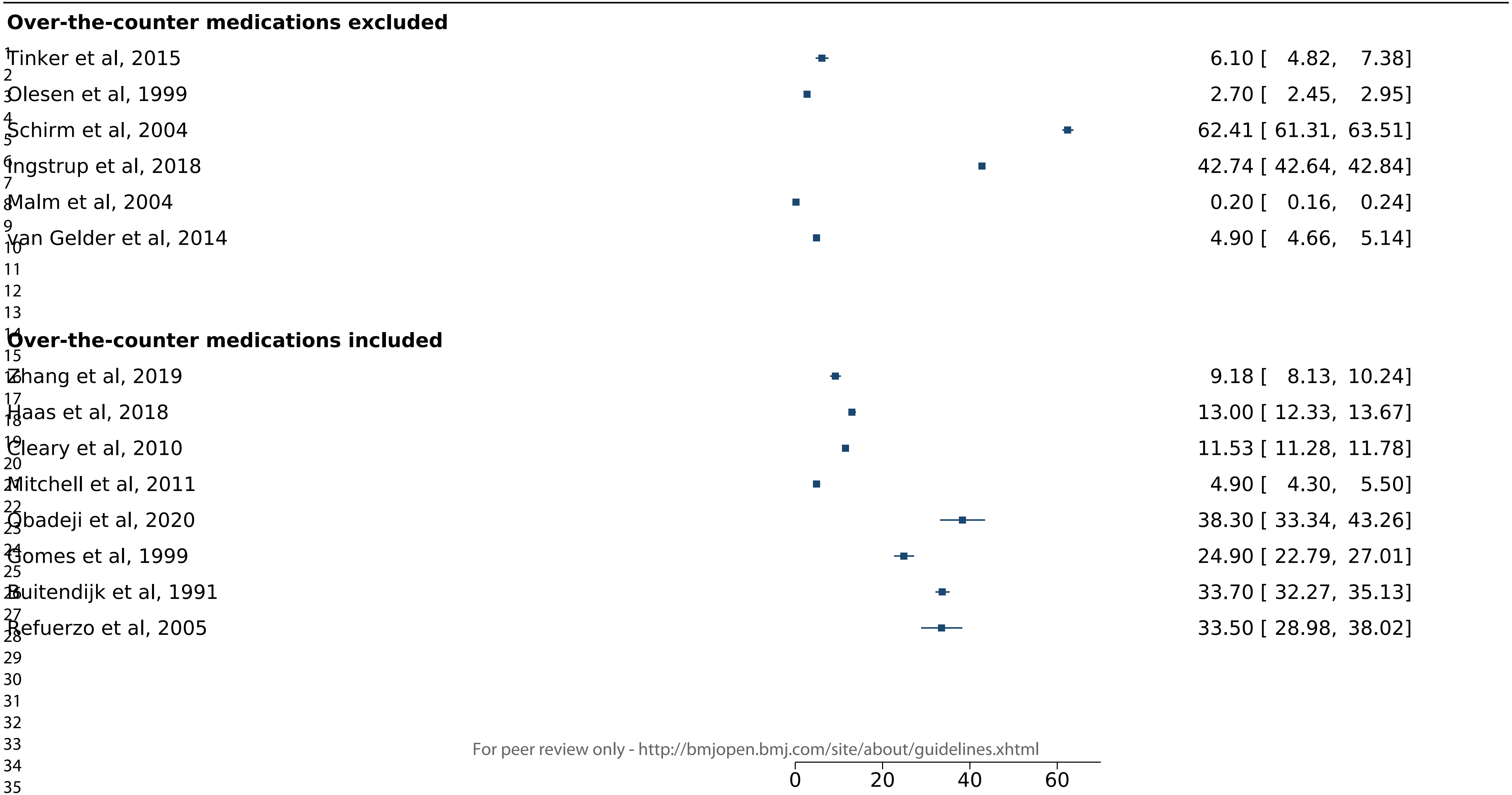

For peer review only - http://bmjopen.bmj.com/site/about/guidelines.xhtml

Table S1- Newcastle-Ottawa Quality Assessment Scale

	Selection		Outcome		
**Author**	**Representativeness of the cohort**	**Ascertainment of pregnancy**	**Assessment of polypharmacy**	**Was follow-up long enough**	**Adequacy of follow-up**
**Haas 2018 (6)**	*	*	-	*	*
**Van Gelder 2014 (19)**	*	-	*	*	*
**Refuerzo 2005 (20)**	*	*	-	*	*
**Gomes 1999 (21)**	*	*	-	*	*
**Tinker 2016 (22)**	-	-	-	*	*
**Malm 2004 (23)**	*	*	*	*	*
**Ingstrup 2018 (24)**	*	*	*	*	*
**Cleary 2010 (25)**	*	*	-	*	*
**Mitchell 2011 (26)**	*	*	-	*	*
**Zhang 2019 (27)**	*	*	*	*	*
**Buitendijk 1991 (28)**	*	*	-	*	*
**Obadeji 2020 (29)**	*	*	*	*	*
**Olesen 1998 (30)**	*	*	*	*	*
**Schirm 2004 (31)**	*	-	*	*	*

* Indicates adequate quality in domain. A maximum of one star can be given for each domain

For peer review only

Appendix S1 – Search strategy

The search strategy for Embase is shows below.

1. polypharmacy/
2. multiple medicatio*.mp.
3. multiple medicine*.mp.
4. multiple drug*.mp.
5. many medicatio*.mp.
6. many medicine*.mp.
7. many drug*.mp.
8. (more adj4 medication*).mp.
9. polydrug*.mp.
10. polymedication.mp.
11. polypharmacy.mp.
12. multi-drug therapy.mp.
13. multidrug therapy.mp.
14. multiple pharmacotherapy.mp.
15. poly pharmacy.mp.
16. polypragmasia.mp.
17. polypragmasy.mp.
18. exp pregnancy/
19. exp Pregnancy Complications/ or exp Pregnancy Disorders/
20. pregnan*.mp.
21. mothers/
22. perinatal.mp.
23. maternal.mp.
24. obstetric*.mp.
25. or/1-17
26. or/18-24
27. 25 and 26

For peer review only

Appendix S2 – Prisma Checklist

Section and Topic	Item #	Checklist item	Location where item is reported
**TITLE**			
Title	1	Identify the report as a systematic review.	Title, abstract, methods
**ABSTRACT**			
Abstract	2	See the PRISMA 2020 for Abstracts checklist.	See below
**INTRODUCTION**			
Rationale	3	Describe the rationale for the review in the context of existing knowledge.	Introduction
Objectives	4	Provide an explicit statement of the objective(s) or question(s) the review addresses.	Introduction
**METHODS**			
Eligibility criteria	5	Specify the inclusion and exclusion criteria for the review and how studies were grouped for the syntheses.	Methods – eligibility criteria
Information sources	6	Specify all databases, registers, websites, organisations, reference lists and other sources searched or consulted to identify studies. Specify the date when each source was last searched or consulted.	Methods – search strategy
Search strategy	7	Present the full search strategies for all databases, registers and websites, including any filters and limits used.	Methods – search strategy, Appendix S1
Selection process	8	Specify the methods used to decide whether a study met the inclusion criteria of the review, including how many reviewers screened each record and each report retrieved, whether they worked independently, and if applicable, details of automation tools used in the process.	Methods - study selection and data abstraction, author contribution
Data collection process	9	Specify the methods used to collect data from reports, including how many reviewers collected data from each report, whether they worked independently, any processes for obtaining or confirming data from study investigators, and if applicable, details of automation tools used in the process.	Methods - study selection and data abstraction, author contribution
Data items	10a	List and define all outcomes for which data were sought. Specify whether all results that were compatible with each outcome domain in each study were sought (e.g., for all measures, time points, analyses), and if not, the methods used to decide which results to collect.	Methods - study selection and data abstraction, outcome measurement
	10b	List and define all other variables for which data were sought (e.g., participant and intervention characteristics, funding sources). Describe any assumptions made about any missing or unclear information.	Methods - study selection and data

For peer review only - http://bmjopen.bmj.com/site/about/guidelines.xhtml

Section and Topic	Item #	Checklist item	Location where item is reported
			abstraction, exclusion criteria
Study risk of bias assessment	11	Specify the methods used to assess risk of bias in the included studies, including details of the tool(s) used, how many reviewers assessed each study and whether they worked independently, and if applicable, details of automation tools used in the process.	Methods - study selection and data abstraction
Effect measures	12	Specify for each outcome the effect measure(s) (e.g., risk ratio, mean difference) used in the synthesis or presentation of results.	Methods – outcome measurement
Synthesis methods	13a	Describe the processes used to decide which studies were eligible for each synthesis (e.g., tabulating the study intervention characteristics and comparing against the planned groups for each synthesis (item #5)).	Methods - study selection and data abstraction and summary measures and results synthesis
	13b	Describe any methods required to prepare the data for presentation or synthesis, such as handling of missing summary statistics, or data conversions.	Methods - summary measures and results synthesis
	13c	Describe any methods used to tabulate or visually display results of individual studies and syntheses.	Methods - summary measures and results synthesis
	13d	Describe any methods used to synthesize results and provide a rationale for the choice(s). If meta-analysis was performed, describe the model(s), method(s) to identify the presence and extent of statistical heterogeneity, and software package(s) used.	Methods - summary measures and results synthesis
	13e	Describe any methods used to explore possible causes of heterogeneity among study results (e.g., subgroup analysis, meta-regression).	N/a
	13f	Describe any sensitivity analyses conducted to assess robustness of the synthesized results.	N/a
Reporting bias assessment	14	Describe any methods used to assess risk of bias due to missing results in a synthesis (arising from reporting biases).	Methods - study selection and data abstraction (risk of bias)
Certainty assessment	15	Describe any methods used to assess certainty (or confidence) in the body of evidence for an outcome.	Methods - study selection and data abstraction (risk of bias)
**RESULTS**			
Study selection	16a	Describe the results of the search and selection process, from the number of records identified in the search to the number of studies included in the review, ideally using a flow diagram.	Figure 1, Results

For peer review only

Section and Topic	Item #	Checklist item	Location where item is reported
	16b	Cite studies that might appear to meet the inclusion criteria, but which were excluded, and explain why they were excluded.	Results, references
Study characteristics	17	Cite each included study and present its characteristics.	Results, Table s1
Risk of bias in studies	18	Present assessments of risk of bias for each included study.	Table S1, Results – risk of bias
Results of individual studies	19	For all outcomes, present, for each study: (a) summary statistics for each group (where appropriate) and (b) an effect estimate and its precision (e.g., confidence/credible interval), ideally using structured tables or plots.	Results, Figures 3-4
Results of syntheses	20a	For each synthesis, briefly summarise the characteristics and risk of bias among contributing studies.	Results
	20b	Present results of all statistical syntheses conducted. If meta-analysis was done, present for each the summary estimate and its precision (e.g. confidence/credible interval) and measures of statistical heterogeneity. If comparing groups, describe the direction of the effect.	N/a
	20c	Present results of all investigations of possible causes of heterogeneity among study results.	Results
	20d	Present results of all sensitivity analyses conducted to assess the robustness of the synthesized results.	Results
Reporting biases	21	Present assessments of risk of bias due to missing results (arising from reporting biases) for each synthesis assessed.	Results – risk of bias
Certainty of evidence	22	Present assessments of certainty (or confidence) in the body of evidence for each outcome assessed.	Results, Figures 3-4
**DISCUSSION**			
Discussion	23a	Provide a general interpretation of the results in the context of other evidence.	Discussion - interpretation
	23b	Discuss any limitations of the evidence included in the review.	Discussion – strengths and limitations
	23c	Discuss any limitations of the review processes used.	Discussion – strengths and limitations
	23d	Discuss implications of the results for practice, policy, and future research.	Conclusion
**OTHER INFORMATION**			
Registration and protocol	24a	Provide registration information for the review, including register name and registration number, or state that the review was not registered.	Methods – protocol and registration
	24b	Indicate where the review protocol can be accessed, or state that a protocol was not prepared.	Methods – protocol and registration and references

Section and Topic	Item #	Checklist item	Location where item is reported
	24c	Describe and explain any amendments to information provided at registration or in the protocol.	N/a
Support	25	Describe sources of financial or non-financial support for the review, and the role of the funders or sponsors in the review.	Funding
Competing interests	26	Declare any competing interests of review authors.	Disclosure of interest
Availability of data, code and other materials	27	Report which of the following are publicly available and where they can be found: template data collection forms; data extracted from included studies; data used for all analyses; analytic code; any other materials used in the review.	N/a

From: Page MJ, McKenzie JE, Bossuyt PM, Boutron I, Hoffmann TC, Mulrow CD, et al. The PRISMA 2020 statement: an updated guideline for reporting systematic reviews. BMJ 2021;372:n71. doi: 10.1136/bmj.n71

For more information, visit: http://www.prisma-statement.org/

For peer review only

Section and Topic	Item #	Checklist item	Reported (Yes/No)
**TITLE**			
Title	1	Identify the report as a systematic review.	Yes
**BACKGROUND**			
Objectives	2	Provide an explicit statement of the main objective(s) or question(s) the review addresses.	Yes
**METHODS**			
Eligibility criteria	3	Specify the inclusion and exclusion criteria for the review.	Yes
Information sources	4	Specify the information sources (e.g. databases, registers) used to identify studies and the date when each was last searched.	Yes
Risk of bias	5	Specify the methods used to assess risk of bias in the included studies.	Yes
Synthesis of results	6	Specify the methods used to present and synthesise results.	Yes
**RESULTS**			
Included studies	7	Give the total number of included studies and participants and summarise relevant characteristics of studies.	Yes
Synthesis of results	8	Present results for main outcomes, preferably indicating the number of included studies and participants for each. If meta-analysis was done, report the summary estimate and confidence/credible interval. If comparing groups, indicate the direction of the effect (i.e. which group is favoured).	Yes
**DISCUSSION**			
Limitations of evidence	9	Provide a brief summary of the limitations of the evidence included in the review (e.g. study risk of bias, inconsistency and imprecision).	Yes
Interpretation	10	Provide a general interpretation of the results and important implications.	Yes
**OTHER**			
Funding	11	Specify the primary source of funding for the review.	Yes
Registration	12	Provide the register name and registration number.	Prospero protocol cited in methods and references

From: Page MJ, McKenzie JE, Bossuyt PM, Boutron I, Hoffmann TC, Mulrow CD, et al. The PRISMA 2020 statement: an updated guideline for reporting systematic reviews. BMJ 2021;372:n71. doi: 10.1136/bmj.n71

For peer review only

PRISMA 2020 Checklist

Section and Topic	Item #	Checklist item	Location where item is reported
**TITLE**			
Title	1	Identify the report as a systematic review.	Title p1
**ABSTRACT**			
Abstract	2	See the PRISMA 2020 for Abstracts checklist.	Abstract p2
**INTRODUCTION**			
Rationale	3	Describe the rationale for the review in the context of existing knowledge.	Introduction p5
Objectives	4	Provide an explicit statement of the objective(s) or question(s) the review addresses.	Introduction p5
**METHODS**			
Eligibility criteria	5	Specify the inclusion and exclusion criteria for the review and how studies were grouped for the syntheses.	Methods p6
Information sources	6	Specify all databases, registers, websites, organisations, reference lists and other sources searched or consulted to identify studies. Specify the date when each source was last searched or consulted.	Methods p6
Search strategy	7	Present the full search strategies for all databases, registers and websites, including any filters and limits used.	Appendix S1
Selection process	8	Specify the methods used to decide whether a study met the inclusion criteria of the review, including how many reviewers screened each record and each report retrieved, whether they worked independently, and if applicable, details of automation tools used in the process.	Methods p7
Data collection process	9	Specify the methods used to collect data from reports, including how many reviewers collected data from each report, whether they worked independently, any processes for obtaining or confirming data from study investigators, and if applicable, details of automation tools used in the process.	Methods p7
Data items	10a	List and define all outcomes for which data were sought. Specify whether all results that were compatible with each outcome domain in each study were sought (e.g. for all measures, time points, analyses), and if not, the methods used to decide which results to collect.	Methods p7
	10b	List and define all other variables for which data were sought (e.g. participant and intervention characteristics, funding sources). Describe any assumptions made about any missing or unclear information.	Methods p7
Study risk of bias assessment	11	Specify the methods used to assess risk of bias in the included studies, including details of the tool(s) used, how many reviewers assessed each study and whether they worked independently, and if applicable, details of automation tools used in the process.	Methods p7
Effect measures	12	Specify for each outcome the effect measure(s) (e.g. risk ratio, mean difference) used in the synthesis or presentation of results.	Methods p7
Synthesis methods	13a	Describe the processes used to decide which studies were eligible for each synthesis (e.g. tabulating the study intervention characteristics and comparing against the planned groups for each synthesis (item #5)).	Methods p7
	13b	Describe any methods required to prepare the data for presentation or synthesis, such as handling of missing summary statistics, or data conversions.	Methods p7
	13c	Describe any methods used to tabulate or visually display results of individual studies and syntheses.	Methods p7
	13d	Describe any methods used to synthesize results and provide a rationale for the choice(s). If meta-analysis was performed, describe the model(s), method(s) to identify the presence and extent of statistical heterogeneity, and software package(s) used.	Methods p7
	13e	Describe any methods used to explore possible causes of heterogeneity among study results (e.g. subgroup analysis, meta-regression).	N/A
	13f	Describe any sensitivity analyses conducted to assess robustness of the synthesized results.	N/A
Reporting bias	14	Describe any methods used to assess risk of bias due to missing results in a synthesis (arising from reporting biases).	N/A

For peer review only

PRISMA 2020 Checklist

Section and Topic	Item #	Checklist item	Location where item is reported
assessment			narrative synthesis
Certainty assessment	15	Describe any methods used to assess certainty (or confidence) in the body of evidence for an outcome.	NA narrative synthesis
**RESULTS**			
Study selection	16a	Describe the results of the search and selection process, from the number of records identified in the search to the number of studies included in the review, ideally using a flow diagram.	Figure 1
	16b	Cite studies that might appear to meet the inclusion criteria, but which were excluded, and explain why they were excluded.	Results p8
Study characteristics	17	Cite each included study and present its characteristics.	Results p8 Table 1
Risk of bias in studies	18	Present assessments of risk of bias for each included study.	Results p8
Results of individual studies	19	For all outcomes, present, for each study: (a) summary statistics for each group (where appropriate) and (b) an effect estimate and its precision (e.g. confidence/credible interval), ideally using structured tables or plots.	Table 1
Results of syntheses	20a	For each synthesis, briefly summarise the characteristics and risk of bias among contributing studies.	N/A
	20b	Present results of all statistical syntheses conducted. If meta-analysis was done, present for each the summary estimate and its precision (e.g. confidence/credible interval) and measures of statistical heterogeneity. If comparing groups, describe the direction of the effect.	N/A
	20c	Present results of all investigations of possible causes of heterogeneity among study results.	N/A
	20d	Present results of all sensitivity analyses conducted to assess the robustness of the synthesized results.	N/A
Reporting biases	21	Present assessments of risk of bias due to missing results (arising from reporting biases) for each synthesis assessed.	N/A
Certainty of evidence	22	Present assessments of certainty (or confidence) in the body of evidence for each outcome assessed.	N/A
**DISCUSSION**			
Discussion	23a	Provide a general interpretation of the results in the context of other evidence.	Discussion p12
	23b	Discuss any limitations of the evidence included in the review.	Discussion p11
	23c	Discuss any limitations of the review processes used.	Discussion p11
	23d	Discuss implications of the results for practice, policy, and future research.	Discussion p12
**OTHER INFORMATION**			
Registration and protocol	24a	Provide registration information for the review, including register name and registration number, or state that the review was not registered.	Methods p6
	24b	Indicate where the review protocol can be accessed, or state that a protocol was not prepared.	Methods p6
	24c	Describe and explain any amendments to information provided at registration or in the protocol.	N/A

For peer review only

PRISMA 2020 Checklist

Section and Topic	Item #	Checklist item	Location where item is reported
Support	25	Describe sources of financial or non-financial support for the review, and the role of the funders or sponsors in the review.	Statements and declarations p13
Competing interests	26	Declare any competing interests of review authors.	Statements and declarations p13
Availability of data, code and other materials	27	Report which of the following are publicly available and where they can be found: template data collection forms; data extracted from included studies; data used for all analyses; analytic code; any other materials used in the review.	Supporting information

From: Page MJ, McKenzie JE, Bossuyt PM, Boutron I, Hoffmann TC, Mulrow CD, et al. The PRISMA 2020 statement: an updated guideline for reporting systematic reviews. BMJ 2021;372:n71. doi: 10.1136/bmj.n71

For more information, visit: http://www.prisma-statement.org/

For peer review only

BMJ Open

Prevalence of polypharmacy in pregnancy: a systematic review

Journal:	*BMJ Open*
Manuscript ID	bmjopen-2022-067585.R1
Article Type:	Original research
Date Submitted by the Author:	03-Nov-2022
Complete List of Authors:	Anand, Astha; University of Birmingham Institute of Applied Health Research<br>Phillips, Katherine; University of Birmingham, Applied health research<br>Subramanian, Anuradhaa; University of Birmingham Institute of Applied Health Research<br>Lee, Siang Ing; University of Birmingham Institute of Applied Health Research<br>Wang, Zhaonan; University of Birmingham Institute of Applied Health Research<br>McCowan, Rebecca; University of Glasgow<br>Agrawal, Utkarsh; University of St Andrews, School of Medicine<br>Fagbamigbe, Adeniyi; University of St Andrews, PBS; University of Ibadan, EMS<br>Nelson-Piercy, Catherine; Guy's and St Thomas' NHS Foundation Trust<br>Brocklehurst, Peter; University of Birmingham Institute of Applied Health Research<br>Damase-Michel, Christine; CHU Toulouse, CHU Toulouse, Université Toulouse III<br>Loane , Maria ; University of Ulster Institute of Nursing and Health Research<br>Nirantharakumar, Krishnarajah; University of Birmingham Institute of Applied Health Research<br>Azcoaga-Lorenzo, Amaya; University of Saint Andrews School of Medicine, Division of Population and Behavioural Sciences
**Primary Subject Heading**:	Obstetrics and gynaecology
Secondary Subject Heading:	Pharmacology and therapeutics, Epidemiology
Keywords:	OBSTETRICS, Maternal medicine < OBSTETRICS, EPIDEMIOLOGY

SCHOLARONE™
Manuscripts

I, the Submitting Author has the right to grant and does grant on behalf of all authors of the Work (as defined in the below author licence), an exclusive licence and/or a non-exclusive licence for contributions from authors who are: i) UK Crown employees; ii) where BMJ has agreed a CC-BY licence shall apply, and/or iii) in accordance with the terms applicable for US Federal Government officers or employees acting as part of their official duties; on a worldwide, perpetual, irrevocable, royalty-free basis to BMJ Publishing Group Ltd ("BMJ") its licensees and where the relevant Journal is co-owned by BMJ to the co-owners of the Journal, to publish the Work in this journal and any other BMJ products and to exploit all rights, as set out in our licence.

The Submitting Author accepts and understands that any supply made under these terms is made by BMJ to the Submitting Author unless you are acting as an employee on behalf of your employer or a postgraduate student of an affiliated institution which is paying any applicable article publishing charge ("APC") for Open Access articles. Where the Submitting Author wishes to make the Work available on an Open Access basis (and intends to pay the relevant APC), the terms of reuse of such Open Access shall be governed by a Creative Commons licence – details of these licences and which Creative Commons licence will apply to this Work are set out in our licence referred to above.

Other than as permitted in any relevant BMJ Author's Self Archiving Policies, I confirm this Work has not been accepted for publication elsewhere, is not being considered for publication elsewhere and does not duplicate material already published. I confirm all authors consent to publication of this Work and authorise the granting of this licence.

For peer review only

Prevalence of polypharmacy in pregnancy: a systematic review

Running title: Polypharmacy in Pregnancy

Authors: Astha Anand[1*], Katherine Phillips[1], Anuradhaa Subramanian[1], Siang Ing Lee[1], Zhaonan Wang[1], Rebecca McCowan[3], Utkarsh Agrawal[2], Adeniyi Francis Fagbamigbe[2, 4], Catherine Nelson-Piercy [5], Peter Brocklehurst[1], Christine Damase-Michel[6,7], Maria Loane[8], Krishnarajah Nirantharakumar[1,9], Amaya Azcoaga-Lorenzo[2#] on behalf of the MuM-PreDiCT Group

* First author
# Senior author

Affiliations

1. Institute of Applied Health Research, University of Birmingham, Birmingham, UK.
2. Division of Population and Behavioural Sciences, School of Medicine, University of St Andrews, UK
3. University of Glasgow College of Medicine, Veterinary & Life Sciences, University of Glasgow, Glasgow, UK.
4. Department of Epidemiology and Medical Statistics, College of Medicine, University of Ibadan, Nigeria
5. Guy's and St. Thomas' NHS Foundation Trust, UK
6. Medical and Clinical Pharmacology, School of Medicine, Université Toulouse III, France
7. INSERM, Center for Epidemiology and Research in Population Health (CERPOP), CIC 1436, France
8. The Institute of Nursing and Health Research, Ulster University, UK
9. Midlands Health Data Research UK, Birmingham, UK.

Orcid ID

Astha Anand 0000-0003-0494-321X, Amaya Azcoaga-Lorenzo 0000-0003-3307-878X, Katherine Phillips 0000-0003-0674-605X , Anuradhaa Subramanian 0000-0001-8875- 7363, Siang Ing Lee 0000-0002-2332-5452, Rebecca McCowan 0000-0002-2379-892X, Utkarsh Agrawal 0000-0001-5181-6120, Adeniyi Francis Fagbamigbe 0000-0001-9184-8258, , , Catherine Nelson-Piercy 0000-0001-9311-1196, Peter Brocklehurst 0000-0002-9950-6751, Christine Damase-Michel 0000-0001-5018-0108, Maria Loane 0000-0002-1206-3637, Helen Dolk 0000-0001-6639-5904, Krishnarajah Nirantharakumar 0000-0002-6816-1279

For peer review only - http://bmjopen.bmj.com/site/about/guidelines.xhtml

Corresponding author: Amaya Azcoaga-Lorenzo, Division of Population and Behavioural Sciences, School of Medicine, University of St Andrews, KY16 9AJ, UK. Email: aal22@st-andrews.ac.uk.

Word count: 2945

Abstract

Objectives

The use of medications amongst pregnant women has been rising over the past few decades but the reporting of polypharmacy has been sporadic. The objective of this review is to identify literature reporting the prevalence of polypharmacy amongst pregnant women, the prevalence of multimorbidity in women taking multiple medications in pregnancy, and associated effects on maternal and offspring outcomes.

Design

MEDLINE and Embase were searched from their inception up to 14th September 2021for intervention al trials, observational studies and systematic reviews reporting on the prevalence of polypharmacy or the use of multiple medications in pregnancy were included. Data on prevalence of polypharmacy, prevalence of multimorbidity, combinations of medications and pregnancy and offspring outcomes were extracted. A descriptive analysis was performed.

Results

Fourteen studies met the review criteria. Prevalence of women being prescribed two or more medications during pregnancy ranged from 4.9% (4.3%-5.5%) to 62.4% (61.3%-63.5%), with a median of 22.5%. For the first trimester, prevalence ranged from 4.9% (4.7%- 5.14%) to 33.7% (32.2%-35.1%). No study reported on prevalence of multimorbidity, or associated pregnancy outcomes in women exposed to polypharmacy.

Conclusion

There is a significant burden of polypharmacy amongst pregnant women. There is a need for evidence on the combinations of medications prescribed in pregnancy, how this specifically affects women with multiple long-term conditions and the associated benefits and harms.

Article Summary

Strengths and Limitations of this study

- A structured and substantial review of the literature, according to a pre-planned and comprehensive search.
- Articles screened rigorous inclusion and exclusion criteria.
- As there is no consensus definition, polypharmacy was reported according to a variety of definitions in this review.

For peer review only - http://bmjopen.bmj.com/site/about/guidelines.xhtml

- Due to the methodological limitations of included studies, it could not be determined whether medications were prescribed concurrently or whether medication was complied with, meaning prevalence of polypharmacy may have been over-estimated
- No studies reporting on maternal or offspring outcomes associated with polypharmacy were found

Funding

This work was funded by the Strategic Priority Fund "Tackling multimorbidity at scale" programme [grant number MR/W014432/1]

Keywords

polypharmacy, pregnancy, maternity, epidemiology, multimorbidity, systematic review

For peer review only

Tweetable abstract

Our systematic review shows significant burden of polypharmacy in pregnancy but outcomes for women and offspring are unknown.

For peer review only

Introduction

Medications may be taken in pregnancy for the management of pregnancy-related symptoms (such as nausea and vomiting), pre-existing maternal health conditions or pregnancy-related complications (1-3). The use of medications amongst pregnant women has been rising over the past few decades (4-6), which could be attributed to a rise in the prevalence of maternal comorbidities, obesity and, in the UK and other high income countries, a rise in the average maternal age (7, 8). With this, the use of multiple medications is also likely to increase (3).Whilst many studies have assessed overall medication use amongst pregnant women, fewer studies have focused on polypharmacy.

Polypharmacy is broadly defined as the use of multiple medications by a single patient, but various definitions are found in the literature. A systematic review of polypharmacy definitions found that studies reported various numerical definitions (ranging from the use of two or more medication to eleven or more medications) and some also incorporated duration or appropriateness of therapy (9). As the number of medications taken together increases, medication interactions and adverse events are expected to increase also. It has been reported that, as the number of medications prescribed together increases, as does the number of potentially serious drug-drug interactions(10). The use of multiple medication has been reported amongst specific subpopulation of pregnant women, such as women with psychiatric illness, epilepsy or human immunodeficiency virus (HIV) (11-13).
However, the polypharmacy rate amongst general population of pregnant women is not as well understood.

Drug pharmacokinetics are altered in pregnancy due to physiological changes in the expectant mothers. For example, expanded plasma volume and maternal body fat in pregnancy increases the volume of distribution for hydrophilic and lipophilic drugs leading to lower plasma concentration. Moreover, increased hepatic and renal clearance during pregnancy can lead to subtherapeutic drug concentrations. (14, 15).

However, few clinical trials are undertaken amongst pregnant women due to concerns around maternal and fetal safety (16, 17). It is therefore, unknown whether polypharmacy during pregnancy will worsen known side effects, result in novel adverse events or, indeed, have a synergistic or beneficial effect (10). Understanding these effects will allow clinicians and women to make more informed decisions about continuing, starting or stopping medications before and during pregnancy.

The objective of this systematic review was to assess the published literature reporting on the prevalence of polypharmacy amongst pregnant women, the prevalence of multimorbidity in women taking multiple medications in pregnancy, and the effect of multiple medication use on maternal and offspring outcomes.

For peer review only - http://bmjopen.bmj.com/site/about/guidelines.xhtml

Methods

A systematic review of the literature was performed in order to identify relevant studies examining the prevalence of polypharmacy in pregnancy, the most common medication combination, rate of multimorbidity and outcomes amongst women exposed to polypharmacy.

Protocol and registration

Protocol for this systematic review has been published on Prospero (Protocol ID CRD42021223966, Available from: https://www.crd.york.ac.uk/prospero/display_record.php?ID=CRD42021223966)(18).

Eligibility Criteria

We included interventional trials, observational studies (cohort studies and case control studies) and systematic reviews reporting the prevalence of polypharmacy or use of multiple medications in pregnant women, where the prevalence of polypharmacy could be extracted from tables or figures. The study authors' definition of polypharmacy was used and we retained the study authors' eligibility criteria for whether over-the-counter medications were included. Where polypharmacy was not defined by the authors of the individual studies, we defined polypharmacy to mean the use of two or more medications.

Exclusion Criteria

We excluded studies focused on specific subpopulations of pregnant women instead of general prevalence of polypharmacy (such as pregnant women with specific medical conditions, or with high-risk pregnancies), as we were interested in the population-based prevalence. We excluded expert opinions, conference abstract, case report, narrative review, laboratory and animal studies. Studies based on non-pregnant women were excluded and unpublished data were not sought.

We did not exclude non-English papers. For any non-English paper identified, native speaker would extract data where possible. Where this was not possible, two independent reviewers (AA and AAL) extracted the data using an online translation service (Google Translate).

Outcome measurement

The primary outcome was prevalence of polypharmacy, as defined by the authors, or the use of two or more medications, where polypharmacy was not defined by the authors.

We also assessed the prevalence of multimorbidity and maternal or offspring outcomes amongst women exposed to polypharmacy. The individual studies' definition of multimorbidity was used where specified. Where the definition of multimorbidity was not specified by the authors, it was defined as the presence of two or more long term health conditions, including mental health conditions.

For peer review only - http://bmjopen.bmj.com/site/about/guidelines.xhtml

Search strategy

MEDLINE was searched for relevant papers from 1946 to 14th September 2021 and Embase was searched from 1974 to 14th September 2021. A librarian helped to develop the search strategy. The full search strategy for Embase is provided in Appendix S1.

Study selection and data extraction

Study selection was conducted in two phases. In the first phase, title and abstracts were screened by two independent reviewers against the eligibility criteria (AA screened all papers, SIL, AS, AAF, UA and ZW were the second reviewers). We retrieved full-text papers for all potentially eligible studies. In the second phase, full-text papers were assessed by two authors independently (AA and AAL) against the eligibility criteria. For all eligible studies, two authors (AA and AAL) independently extracted the data using a piloted data extraction form, and assessed the risk of bias. Discrepancies were reviewed and resolved by a third independent reviewer (ZW).

Data items extracted included: purpose of the study, setting, recruitment, inclusion and exclusion criteria, participant demographics (age, ethnicity, parity, deprivation), definition of polypharmacy, prevalence of polypharmacy, classification system for grouping medications, list of health conditions, follow-up length, any secondary outcomes, funding, and conflict of interest.

We used the Newcastle-Ottawa critical appraisal checklist for observational studies to assess risk of bias in the individual studies during the data extraction stage. (19)

Summary measures and results synthesis

Results are presented as descriptive analysis. Primary outcome is presented as proportion or prevalence. We stratified the analysis according to the various definitions of polypharmacy from the primary studies (e.g., 2 or more medications) and the setting (primary or secondary care). Given the heterogenous nature of the studies, statistical pooling and analysis was not possible. PRISMA checklist for reporting of systematic reviews has been followed (Appendix S2).

Patient and Public involvement

Patients were not involved in the development of the research question, study design or selection of outcome measures.

For peer review only - http://bmjopen.bmj.com/site/about/guidelines.xhtml

Results

Study Selection

We screened 2,228 titles and abstracts. Of those, 46 papers were subjected to detailed evaluation in full text screening (6, 20-64), and 14 met inclusion criteria (6, 20-32). The main reasons for exclusion were an inadequate method of reporting prevalence of polypharmacy or reporting on specific subpopulation of pregnant women. The results from each step of the review process are documented in a PRISMA flow diagram (Figure 1).

Study Characteristics

Table 1 shows the characteristics of the included studies. Studies were published between 1991 and 2020. The study populations ranged between 369 and 981,392. Six studies examined prevalence of polypharmacy using administrative data, seven used surveys to collect self-reported medication use. One study used administrative data for prescription medications and self-report for the use of over the counter (OTC) medications.

In seven studies, women were recruited from hospitals (either birth hospital or antenatal clinic). (6, 21, 22, 26, 27, 29, 30) In the other seven studies, participants were sampled from a national registry or population-based database (such as pharmacy records). (20, 23-25, 28, 31, 32)

Mitchell et al. reported results from two different cohorts; Birth Defect Study (BDS) and National Birth Defects Prevention Study (NBDPS). Both studies contain data from mothers of babies born with birth defects and from a control group of mothers of babies born without birth defects. Mitchell et al reported data from both cases and controls in the BDS and from just the controls of the NBDPS. As pregnancies of mothers of babies born with birth defects are unlikely to be representative of the general population of pregnant women, only data from NBDPS were included in the results of this review.

For peer review only

Table 1 – List of included studies and study characteristics

Author	Study Design	Country / Location	Inclusion criteria	Source (administrative data/self-reported)	Total number of pregnancies	Trimester studied	Polypharmacy definition used in study	Definition of polypharmacy used in review	Medications included or excluded	Prevalence reported
Buitendijk et al (1991) (29)	Retrospective survey	USA	All women who made their first prenatal visit to private obstetric or midwifery practice, a health maintenance organization, or a hospital clinic and were scheduled for delivery at Yale-New Haven Hospital	Self-report	4186	Early pregnancy (first trimester)	Polypharmacy not defined by author	≥ 2	Included OTC medications Excluded vitamins and minerals	33.70%
Olesen et al (1998) (31)	Retrospective cohort	Denmark	Primiparous women identified through Danish National Birth Registry	Administrative data	16001	Across the three trimesters	More than 3 medications	≥ 4 (as defined by the authors)	Excluded vitamins and minerals	2.70%
Gomes et al (1999) (22)	Retrospective survey	Brazil	Pregnant women who gave birth in one of 5 participating hospitals	Self-report	1620	Across the three trimesters	Polypharmacy not defined by author	>6	Included OTC medications Excluded vitamins and minerals	24.90%
Malm et al (2004) (24)	A retrospective, register-based cohort study	Finland	All women who applied for maternal grants in 1999 and the mother has visited a maternity clinic before the end of the fourth month	Administrative record	43470	Across the three trimesters	Polypharmacy not defined by author	≥ 10	Included some, but not all, OTC medications	0.20%
Schirm et al (2004) (32)	Cross-sectional study	Netherlands	Female person (15-50 years older than child) at the same address as child aged 0 -5 years, with no other female at the address	Administrative data	7500	Across the three trimesters	Polypharmacy not defined by author	≥ 2	Excluded OTC medications	62.41%
Refuerzo et al (2005) (21)	Prospective observational	USA	Women who gave birth at a single, university-based, tertiary-care hospital	Self-report	418	Across the three trimesters	Polypharmacy not defined by author	≥ 2	Included OTC medications	33.50%
Cleary et al (2010) (26)	Retrospective cohort	Ireland	Pregnancy booking and midwife care at tertiary level hospital	Self-report	61252	Early pregnancy (first trimester)	Polypharmacy not defined by author	≥ 2	Included OTC medications	29.40%

For peer review only - http://bmjopen.bmj.com/site/about/guidelines.xhtml

Mitchell et al (2011) (NBDPS Study Arm Reported) (27)	Cross-sectional study	USA and Canada	NBDPS study- controls were randomly selected from birth certificates or from birth hospitals	Self-report	5008	Across the three trimesters	Polypharmacy not defined by author	≥ 4	Included OTC medications	4.90%
van Gelder et al (2014) (20)	Retrospective cohort study	Netherlands	Female person (15-50 years older than child) at the same address as child aged 0 -5 years, with no other female at the address	Administrative record	32016	First trimester	Polypharmacy not defined by author	≥ 2	Excluded vitamins and minerals	4.90%
Tinker et al (2016) (23)	Cross-sectional surveys	USA	Non-institutionalised civilian women aged 15-44	Self-report	1350	Prior 30 days (Pregnancies across three trimesters)	Polypharmacy not defined by author	≥ 2	Excluded vitamins and minerals	6.10%
Haas et al (2018) (6)	Prospective longitudinal cohort study	USA	Primiparous women, aged 13 or over, in the first trimester	Self-report	9546	Across the three trimesters	≥ 5 medications during the same epoch	≥ 5 (as defined by the authors)	Included OTC medications Analysed medication used when vitamins and minerals included and excluded	13%
Ingstrup et al (2018) (25)	Population based-descriptive study	Denmark	Pregnancies ending in live-born singletons during 1997-2012 to women aged between 15-55	Administrative record	981392	Across the three trimesters	Polypharmacy not defined by author	≥ 2	None mentioned	42.74%
Zhang et al. (2019) (28)	Retrospective cohort	China	Singleton deliveries, mothers aged between 12 to 54	Administrative data	7946 (2896 pregnancies covering all three trimesters)	Across the three trimesters	Polypharmacy not defined by author	≥ 2	Included OTC medications	9.19%
Obadeji et al (2020) (30)	Cross sectional study	Nigeria	All consecutive consenting women who came for outpatient antenatal care at a secondary health care facility	Administrative data for prescription drug and self-report for OTC	369	Cross-sectional (Pregnancies across three trimesters)	Polypharmacy not defined by author	≥ 3	Included OTC medications	38.30%

For peer review only - http://bmjopen.bmj.com/site/about/guidelines.xhtml

Risk of bias within studies

Most of the study cohorts were considered representative of the population they were sampling from. Most studies ascertained pregnancy status using hospital or pharmacy records or from birth registries, which were considered likely to be accurate. Schirm et al and Van Gelder et al used a pharmacy database to identify all children born within a given timeframe (20) (32).Women of reproductive age living at the same address as the child were identified in the database and their prescription data was collected for the 270 days before the child's date of birth. There is a chance that women could have been misclassified as pregnant if the child was not living with their biological mother.

As discussed above, seven studies relied solely on self-reported medication use to measure outcomes, introducing the potential for recall bias (6, 21, 22, 26, 27, 29, 30). The follow-up period was considered adequate for each study. Nine studies reported multiple medication use across the entire pregnancy (6, 20, 21, 23, 24, 26, 27, 30, 31), while three studies reported for early pregnancy (first trimester) only (19, 25, 28). Obadeji et al and Tinker et al employed a cross-sectional design and included women across all trimesters. (22, 29) Follow-up rates were considered adequate for all studies, with no study having significant numbers of subjects lost to follow up. Table S1 shows the outcome of the risk of bias assessment.

Prevalence of polypharmacy

The prevalence of polypharmacy-ranged from 0.2% - 62.4%, with a median value of 12.3%. Exclusion of over-the-counter drugs does not change the spread of the prevalence of polypharmacy.

Prevalence by polypharmacy definition

The prevalence of polypharmacy, defined as the use or 2 or more medications, ranged from 4.9% (4.3%-5.5%) to 61.3% (61.3%-63.5%) based on eight papers, with a median value of 22.5% (20, 21, 23, 25, 26, 28, 29, 32) (Figure 2). Only two studies explicitly defined polypharmacy. Olesen et al. defined it as the use of four or more medications (prevalence 2.7%) and Haas et al. defined it as the use of five or more medications (prevalence 13%) (6, 31).

Other studies did not define polypharmacy, but stratified results by the number of medications taken (Figure 2). Mitchell et al and Gomes et al did not define polypharmacy and only reported the use of four or more medications (15.7%) and six or more drugs (24.9%), respectively. (22, 27) Malm et al (2004) reported that 0.2% of women purchased ten or more different medications during the whole period of pregnancy. (24) Due to heterogeneity within the data, meta-analysis was not undertaken.

Prevalence of Polypharmacy by Trimester

Two studies, Obadeji et al and Zhang et al, reported polypharmacy use across the whole pregnancy and also subdivided into trimesters. For these two studies, polypharmacy prevalence across the whole pregnancy has been summarised. (28, 30) Obadeji et al reported a prevalence of 50.0% (95% CI 21.1%-79.0%) in the first trimester compared to a prevalence of 39.8% (95% CI 34.8%-44.8%) across all three trimesters. Zhang et al reported a

For peer review only - http://bmjopen.bmj.com/site/about/guidelines.xhtml

prevalence of 3.8%% (95% CI 3.1%-4.6%) in the first trimester compared to a prevalence of 9.2% (95% CI 8.3%-10.2%) across all three trimesters.

Due to the design and nature of the study, Van Gelder et al, Cleary et al and Buitendijk et al have reported medication use during early pregnancy or the first trimester period only, reporting polypharmacy prevalence of 4.9% (95% CI 4.7%-5.1%), 11.5% (95% CI 11.3%-11.8%) and 33.7% (95% CI 32.2%-35.1%). (20, 29) In a cross-sectional study, Tinker et al. cover medication use in the last 30 days only but across the whole pregnancy. (23) Olesen et al cover a period from 12 weeks prenatal to 12 weeks postpartum in the analysis. (31) Figure 3 shows polypharmacy prevalence when including studies which covered the entire duration of pregnancy.

Prevalence of polypharmacy by Medications included

Whilst most of the studies reported any possible medication use, van Gelder et al report only the teratogenic medications used and not all possible medications. (20)

Over-the-counter medications

Eight studies include over-the-counter medications in their analysis – results for polypharmacy prevalence, subdivided by inclusion of over-the-counter drugs, are shown in Figure 4. (6, 21, 22, 26-30) Reported prevalence of polypharmacy for studies that included OTC medications ranged from 4.9% (Mitchell et al (95% CI 4.3%-5.5%)) to 38.3% (Obadeji et al (95% CI 33.3%-43.3%)). Reported prevalence of polypharmacy for studies that excluded OTC medications ranged from 0.2% (Malm et al (95% CI 0.2%-0.2%) to 62.4% (Schirm et al (61.3%-63.5%)). Of note, Malm et al include some but not all OTC medications, as some medications were reimbursable and therefore were included in the national medication prescription register used for the study. (24)

Exclusion of vitamins and minerals

Five studies specifically excluded vitamins and minerals (such as folic acid and iron) from the study design. (20, 22, 23, 29, 31) The definition of routine prenatal vitamins or minerals was determined by the authors of the original studies. Haas et al analysed medication use, when vitamins and minerals were included and excluded. When including vitamins and minerals, Haas et al report 30.5% (95% CI 29.6%-31.5%) of women use 5 or more medication; whereas, only 13% (95% CI 12.3%-13.7%) use 5 or more medications if vitamins and minerals are excluded. (6)

Medications used during pregnancy

The most commonly prescribed or taken medications described in the studies were anti-emetics (6, 23) (27), antibiotics (6) (27) (28) (29) (30) (31) (32) analgesia (6, 23) (27) and antacids (23) (30) (32) and vitamins or supplements (6) (29) (32) However, no studies specified which medications were used in combination or were used by women exposed to polypharmacy.

Multimorbidity and maternal or offspring outcomes

No studies were found describing which conditions women who were exposed to polypharmacy were treated for, and none specify how many women had multimorbidity or

For peer review only - http://bmjopen.bmj.com/site/about/guidelines.xhtml

long-term illness. No studies were found which reported on maternal or offspring outcomes.

For peer review only

Discussion

Main findings

Studies of multiple medication use in pregnancy reported a wide range in the prevalence of polypharmacy. Where the definition of polypharmacy was two or more medications only, the prevalence of polypharmacy ranged from 5%-62%. However, the definition of polypharmacy was varied, and most studies were not considered truly representative of all pregnant women.

Strengths and limitations

This systematic review has several important strengths. We developed a structured and substantial review of the literature, according to pre-planned and comprehensive search terms with the help of a librarian, who is trained to undertake searches in large database repositories. Screening was conducted according to a rigorous inclusion and exclusion criteria, and we used two independent reviewers for data extraction to minimise bias. Two databases were searched: MEDLINE and Embase. We did not limit our search to studies published in the English language to minimise language bias, although specific databases in languages other than English were not included.

There are limited studies specifically assessing polypharmacy in pregnancy. There is no consensus on the definition of polypharmacy and polypharmacy is often not explicitly defined in the studies. Where polypharmacy is defined, the definition varies from study to study. Only two studies in this systematic review subdivide polypharmacy use in different trimesters. Exclusion of routine prenatal vitamins is often determined by individual authors. Inclusion of OTC medications is variable and often determined by the data available.

The main caveat from these studies is that it is not clear whether use of multiple medication in pregnancy was simultaneous or sequential. Additionally, prescription and dispensation of medications do not equate to compliance. Qualitative studies show that women are less likely to use medications when pregnant, especially if potential risks to the fetus and benefits to the mother have not been adequately communicated (65).

In majority of the studies identified in this systematic review, pregnancy was confirmed retrospectively or identified using birth records. Thus, not all pregnancies were captured and pregnancies resulting in terminations, miscarriages or still birth, were excluded. These pregnancy outcomes are clinically important and the use of multiple medications in these groups warrants further assessment.

Whilst some of the studies outline common medications used by pregnant women overall, none of the studies describe the combinations of medications used in pregnancy. Pregnant women have been described as drug orphans, as they are often excluded from clinical trials. The maternal and offspring outcomes following medication exposure during pregnancy are often determined through retrospective observational studies (16, 17). Association between rates of miscarriage and pre-term birth and medications used during pregnancy have been described in women with major psychiatric illnesses (13) however, none of the studies assessing polypharmacy in this systematic review evaluate the effect of taking multiple medication for the women and their offspring.

For peer review only - http://bmjopen.bmj.com/site/about/guidelines.xhtml

Interpretation

The finding of 5-62% of pregnant women taking two or more medications is in keeping with a previous systematic review of the literature evaluating individual-level exposures to prescription medications in pregnancy. This review, which included only studies from developed (OECD) countries, found 27-93% of women filled at least one prescription during pregnancy reflecting high medication use during pregnancy (66).

The findings of this review should be interpreted with caution. As discussed above, the literature is not necessarily representative of the general pregnant population, inclusion of certain medications was variable and, where polypharmacy was defined, there were differences in the definitions used. This variation is in keeping with the findings of a systematic review of definitions of polypharmacy in older people (9). This review also found that, in some instances, safety and appropriateness of medications were taken into account when defining polypharmacy. This is an important consideration in pregnancy, although, as discussed, there is often not adequate safety information available.

Despite this, the median value of one in five women taking two or more medications, indicates that a significant proportion of women are potentially exposed to multiple medication in pregnancy. The lack of studies into combinations of medications taken during pregnancy and the effects of polypharmacy on maternal and offspring outcomes highlights the urgent need for further research in this area.

Conclusion

The reported prevalence of polypharmacy amongst pregnant women varies based on the number of medications counted in the definition, the trimester considered and the types of medications included. Commonly, only pregnancies resulting in live birth are reported in studies assessing polypharmacy. This systematic review shows relatively large burden of polypharmacy amongst pregnant women and highlights the need to evaluate the outcomes for these women and for their offspring. This is especially relevant for women with multiple, long-term conditions, who are more likely to need multiple medications.

Figures

Figure 1. 2020 PRISMA flow diagram
Figure 2. Forest plot showing prevalence of polypharmacy, subdivided by the definition of polypharmacy (number of medications taken)
Figure 3. Forest plot showing prevalence of polypharmacy (as defined by the study), for studies which covered all trimesters of the pregnancy and the first trimester
Figure 4. Forest plot showing prevalence of polypharmacy, subdivided by inclusion or exclusion of over-the-counter medications

Supporting Information

Table S1. Newcastle-Ottawa Quality Assessment Scale

For peer review only - http://bmjopen.bmj.com/site/about/guidelines.xhtml

Appendix S1. Search strategy.
Appendix S2. Prisma checklist.

Statements and Declarations

Funding

This work was funded by the Strategic Priority Fund "Tackling multimorbidity at scale" programme [grant number MR/W014432/1] delivered by the Medical Research Council and the National Institute for Health Research in partnership with the Economic and Social Research Council and in collaboration with the Engineering and Physical Sciences Research Council.

Competing Interests

None declared

Acknowledgements

The authors thank Ms. Vicki Cormie, Senior Librarian (Academic Liaison) at University of St. Andrews for help with the search strategy. The authors thank Professor Helen Dolk for advice and comments on the manuscript.

Author Contributions

KN, AA and AAL conceived the study and AA, KN and AAL designed the protocol. AA and AAL performed the literature search. AA, ZW, AS, SIL, UA, AAF, RM and AAL selected the studies and extracted the relevant information. AA synthesised the data and wrote the first draft of the paper. AA, KP, SIL, AS, RM, CNP, PB, CDM, ML, KN and AAL critically revised successive drafts of the paper. AA is the guarantor of the review.

Details of ethics approval

None required

Data Sharing Statement

No additional data are available

For peer review only

References

1. Kulkarni J, Worsley R, Gilbert H, Gavrilidis E, Van Rheenen TE, Wang W, et al. A prospective cohort study of antipsychotic medications in pregnancy: The first 147 pregnancies and 100 one year old babies. PLoS ONE. 2014;9(5):e94788.
2. Beeson JG, Homer CSE, Morgan C, Menendez C. Multiple morbidities in pregnancy: Time for research, innovation, and action. PLOS Medicine. 2018;15(9):e1002665.
3. Barnett K, Mercer SW, Norbury M, Watt G, Wyke S, Guthrie B. Epidemiology of multimorbidity and implications for health care, research, and medical education: a cross-sectional study. Lancet. 2012;380(9836):37-43.
4. Mitchell AA, Gilboa SM, Werler MM, Kelley KE, Louik C, Hernandez-Diaz S. Medication use during pregnancy, with particular focus on prescription drugs: 1976-2008. American Journal of Obstetrics and Gynecology. 2011;205(1):51.
5. Headley J, Northstone K, Simmons H, Golding J. Medication use during pregnancy: data from the Avon Longitudinal Study of Parents and Children. Eur J Clin Pharmacol. 2004;60(5):355-61.
6. Haas DM, Marsh DJ, Dang DT, Parker CB, Wing DA, Simhan HN, et al. Prescription and Other Medication Use in Pregnancy. Obstetrics and gynecology. 2018;131(5):789-98.
7. Statistics OfN. Birth characteristics in England and Wales: 2019 ons.gov.uk: Office for National Statistics; 2019 [Available from: https://www.ons.gov.uk/peoplepopulationandcommunity/birthsdeathsandmarriages/livebirths/bulletins/birthcharacteristicsinenglandandwales/2019.
8. MuM-PreDiCT. Epidemiology of pre-existing multimorbidity in pregnant women in the UK in 2018: a cross sectional study using CPRD, SAIL and SMR 2021 [Available from: https://docs.google.com/document/d/1mZf9YSqCIZIX8Og2ROy9epgloYQlabtq/edit.
9. Masnoon N, Shakib S, Kalisch-Ellett L, Caughey GE. What is polypharmacy? A systematic review of definitions. BMC Geriatrics. 2017;17(1):230.
10. Guthrie B, Makubate B, Hernandez-Santiago V, Dreischulte T. The rising tide of polypharmacy and drug-drug interactions: population database analysis 1995–2010. BMC Medicine. 2015;13(1):74.
11. Okoli C, Schwenk A, Radford M, Myland M, Taylor S, Darley A, et al. Polypharmacy and potential drug-drug interactions for people with HIV in the UK from the Climate-HIV database. HIV Med. 2020;21(8):471-80.
12. Kinney MO, Morrow J. Epilepsy in pregnancy. BMJ. 2016;353:i2880.
13. Peindl KS, Masand P, Mannelli P, Narasimhan M, Patkar A. Polypharmacy in pregnant women with major psychiatric illness: a pilot study. J Psychiatr Pract. 2007;13(6):385-92.
14. Feghali M, Venkataramanan R, Caritis S. Pharmacokinetics of drugs in pregnancy. Semin Perinatol. 2015;39(7):512-9.
15. Pariente G, Leibson T, Carls A, Adams-Webber T, Ito S, Koren G. Pregnancy-Associated Changes in Pharmacokinetics: A Systematic Review. PLoS medicine. 2016;13(11):e1002160-e.
16. Scaffidi J, Mol BW, Keelan JA. The pregnant women as a drug orphan: a global survey of registered clinical trials of pharmacological interventions in pregnancy. Bjog. 2017;124(1):132-40.
17. Illamola SM, Bucci-Rechtweg C, Costantine MM, Tsilou E, Sherwin CM, Zajicek A. Inclusion of pregnant and breastfeeding women in research - efforts and initiatives. Br J Clin Pharmacol. 2018;84(2):215-22.

For peer review only - http://bmjopen.bmj.com/site/about/guidelines.xhtml

18. Astha Anand AS, Siang Lee, Krishnarajah Nirantharakumar, Amaya Azcoaga-Lorenzo. Prevalence of polypharmacy in pregnancy and associated health outcomes in mothers and offspring crd.york.ac.uk: Propero (National Institute for Health Research); 2021 [Available from: https://www.crd.york.ac.uk/prospero/display_record.php?ID=CRD42021223966).
19. Wells G, Shea B, O'Connell D, Peterson J, Welch V, Losos M, Tugwell P: The Newcastle-Ottawa Scale (NOS) for assessing the quality of nonrandomised studies in meta-analyses. 2013, http://www.ohri.ca/programs/clinical_epidemiology/oxford.asp,.
20. Van Gelder MMHJ, Bos JHJ, Roeleveld N, De Jong-Van Den Berg LTW. Drugs associated with teratogenic mechanisms. Part I: Dispensing rates among pregnant women in the Netherlands, 1998-2009. Human Reproduction. 2014;29(1):161-7.
21. Refuerzo JS, Blackwell SC, Sokol RJ, Lajeunesse L, Firchau K, Kruger M, et al. Use of over-the-counter medications and herbal remedies in pregnancy. American journal of perinatology. 2005;22(6):321-4.
22. Gomes KR, Moron AF, Silva R, Siqueira AA. Prevalence of use of medicines during pregnancy and its relationship to maternal factors. Revista de saude publica. 1999;33(3):246-54.
23. Tinker SC, Broussard CS, Frey MT, Gilboa SM. Prevalence of prescription medication use among non-pregnant women of childbearing age and pregnant women in the United States: NHANES, 1999-2006. Maternal and child health journal. 2015;19(5):1097-106.
24. Malm H, Martikainen J, Klaukka T, Neuvonen PJ. Prescription of hazardous drugs during pregnancy. Drug safety. 2004;27(12):899-908.
25. Ingstrup KG, Liu X, Gasse C, Debost JCP, Munk-Olsen T. Prescription drug use in pregnancy and variations according to prior psychiatric history. Pharmacoepidemiology and Drug Safety. 2018;27(1):105-13.
26. Cleary BJ, Butt H, Strawbridge JD, Gallagher PJ, Fahey T, Murphy DJ. Medication use in early pregnancy-prevalence and determinants of use in a prospective cohort of women. Pharmacoepidemiology and drug safety. 2010;19(4):408-17.
27. Mitchell AA, Gilboa SM, Werler MM, Kelley KE, Louik C, Hernandez-Diaz S, et al. Medication use during pregnancy, with particular focus on prescription drugs: 1976-2008. American journal of obstetrics and gynecology. 2011;205(1):51.e1-8.
28. Zhang J, Ung COL, Wagner AK, Guan X, Shi L. Medication use during pregnancy in mainland china: A cross-sectional analysis of a national health insurance database. Clinical Epidemiology. 2019;11:1057-65.
29. Buitendijk S, Bracken MB. Medication in early pregnancy: prevalence of use and relationship to maternal characteristics. American journal of obstetrics and gynecology. 1991;165(1):33-40.
30. Obadeji ST, Obadeji A, Bamidele JO, Ajayi FT. Medication use among pregnant women at a secondary health institution: Utilisation patterns and predictors of quantity. African Health Sciences. 2020;20(3):1206-16.
31. Olesen C, Steffensen FH, Nielsen GL, De Jong-Van Den Berg L, Olsen J, Sorensen HT, et al. Drug use in first pregnancy and lactation: A population-based survey among Danish women. European Journal of Clinical Pharmacology. 1999;55(2):139-44.
32. Schirm E, Meijer WM, Tobi H, de Jong-van den Berg LTW. Drug use by pregnant women and comparable non-pregnant women in The Netherlands with reference to the Australian classification system. European journal of obstetrics, gynecology, and reproductive biology. 2004;114(2):182-8.

For peer review only - http://bmjopen.bmj.com/site/about/guidelines.xhtml

33. Alani AHHDA, Suhaimi AM, Hassan BAR, Mohammed AH. Use, awareness, knowledge and beliefs of medication during pregnancy in Malaysia. Osong Public Health and Research Perspectives. 2021;11(6):373-9.
34. Zaki NM, Albarraq AA. Use, attitudes and knowledge of medications among pregnant women: A Saudi study. Saudi Pharmaceutical Journal. 2014;22(5):419-28.
35. Handal M, Engeland A, Ronning M, Skurtveit S, Furu K. Use of prescribed opioid analgesics and co-medication with benzodiazepines in women before, during, and after pregnancy: a population-based cohort study. European journal of clinical pharmacology. 2011;67(9):953-60.
36. Nordeng H, Bayne K, Havnen GC, Paulsen BS. Use of herbal drugs during pregnancy among 600 Norwegian women in relation to concurrent use of conventional drugs and pregnancy outcome. Complementary therapies in clinical practice. 2011;17(3):147-51.
37. Hanley GE, Park M, Oberlander TF. Socieconomic status and psychotropic medicine use during pregnancy: a population-based study in British Columbia, Canada. Archives of Women's Mental Health. 2020;23(5):689-97.
38. Truong BT, Lupattelli A, Kristensen P, Nordeng H. Sick leave and medication use in pregnancy: A European web-based study. BMJ Open. 2017;7(8):e014934.
39. Rouamba T, Valea I, Bognini JD, Kpoda H, Mens PF, Gomes MF, et al. Safety Profile of Drug Use During Pregnancy at Peripheral Health Centres in Burkina Faso: A Prospective Observational Cohort Study. Drugs - Real World Outcomes. 2018;5(3):193-206.
40. Zhang J, Ung COL, Guan X, Shi L. Safety of medication use during pregnancy in mainland China: Based on a national health insurance database in 2015. BMC Pregnancy and Childbirth. 2019;19(1):459.
41. Berard A, Sheehy O. Quebec pregnancy cohort: Prevalence of medication use during gestation and pregnancy outcomes. Therapie. 2014;69(1):71-81.
42. Farooq MO, Reddy SK, Raghu Prasada MS, Nagraj SA, Karupakula S, Natarajan DK. Prescription pattern of the drugs among pregnant inpatients in tertiary care hospital. Journal of Pharmacy Research. 2014;8(7):981-5.
43. Rathod AM, Rathod RM, Jha RK, Gupta VK, Tabish A, Diptendu S. Prescribing trends in antenatal care at a tertiary level teaching hospital of Vidarbha region. Research Journal of Pharmaceutical, Biological and Chemical Sciences. 2012;3(3):865-72.
44. Agarwal M, Nayeem M, Safhi MM, Gupta N, Makeen HA, Sumaily JM. Prescribing pattern of drugs in the department of obstetrics and gynecology in expected mothers in Jazan Region, KSA. International Journal of Pharmacy and Pharmaceutical Sciences. 2014;6(1):658-61.
45. Makiabadi F, Rajeswari R, Jayashree AK. Prescribing Pattern of Drugs in Department of Obstetrics and Gynecology at A Tertiary Care Teaching Hospital, Bangalore, India. Pakistan Journal of Medical and Health Sciences. 2021;15(5):1265-9.
46. Vafai Y, Yeung EH, Hinkle SN, Grewal J, Zhang C, Grantz KL, et al. Prenatal medication use in a prospective pregnancy cohort by pre-pregnancy obesity status. Journal of Maternal-Fetal and Neonatal Medicine. 2021.
47. Sripada R, Suresh Kumar SV, Devanna N, Kandula RR. Pattern of possible drug-drug interactions among different specialties at an indian tertiary care teaching hospital. International Journal of Research in Pharmaceutical Sciences. 2020;11(3):3988-92.
48. Gharoro EP, Igbafe AA. Pattern of drug use amongst antenatal patients in Benin City, Nigeria. Medical science monitor : international medical journal of experimental and clinical research. 2000;6(1):84-7.
49. Lee E, Maneno MK, Smith L, Weiss SR, Zuckerman IH, Wutoh AK, et al. National patterns of medication use during pregnancy. Pharmacoepidemiology and drug safety. 2006;15(8):537-45.

For peer review only - http://bmjopen.bmj.com/site/about/guidelines.xhtml

50. Palmsten K, Hernandez-Diaz S, Chambers CD, Mogun H, Lai S, Gilmer TP, et al. The most commonly dispensed prescription medications among pregnant women enrolled in the U.S. Medicaid Program. Obstetrics and Gynecology. 2015;126(3):465-73.
51. Havard A, Barbieri S, Hanly M, Perez-Concha O, Tran DT, Kennedy D, et al. Medications used disproportionately during pregnancy: Priorities for research on the risks and benefits of medications when used during pregnancy. Pharmacoepidemiology and Drug Safety. 2021;30(1):53-64.
52. Glavind J, Greve T, de Wolff MG, Hansen MK, Henriksen TB. Medication used in Denmark in the latent phase of labor - Do we know what we are doing? Sexual and Reproductive Healthcare. 2020;25:100515.
53. De Jonge L, Zetstra-Van Der Woude PA, Bos HJ, De Jong-Van Den Berg LTW, Bakker MK. Identifying associations between maternal medication use and birth defects using a case-population approach: An exploratory study on signal detection. Drug Safety. 2013;36(11):1069-78.
54. Ilic M, Lupattelli A, Nordeng H. Medical care contact for infertility and related medication use during pregnancy - a european, cross-sectional web-based study. Norsk Epidemiologi. 2021;29(1-2):97-106.
55. Baraka MA, Steurbaut S, Coomans D, Dupont AG. Ethnic differences in drug utilization pattern during pregnancy: A cross-sectional study. Journal of Maternal-Fetal and Neonatal Medicine. 2013;26(9):900-7.
56. Irvine L, Flynn RWV, Libby G, Crombie IK, Evans JMM. Drugs dispensed in primary care during pregnancy: a record-linkage analysis in Tayside, Scotland. Drug safety. 2010;33(7):593-604.
57. Araujo M, Hurault-Delarue C, Sommet A, Damase-Michel C, Benevent J, Lacroix I. Drug prescriptions in French pregnant women between 2015 and 2016: A study in the EGB database. Therapies. 2020.
58. Girit N, Tugrul I, Demirci B, Bozkurt O, Dost T, Birincioglu M, et al. Drug exposure in early pregnancy might be related to the effects of increased maternal progesterone in implantation period. Journal of Psychosomatic Obstetrics and Gynecology. 2018;39(1):7-10.
59. Bornhauser C, Quack Lotscher Katharina C, Seifert B, Simoes-Wust AP. Diet, medication use and drug intake during pregnancy: Data from the consecutive swiss health surveys of 2007 and 2012. Swiss Medical Weekly. 2017;147(51-52):w14572.
60. Galappatthy P, Ranasinghe P, Liyanage CK, Wijayabandara M, Warapitiya DS, Jayakody RL, et al. Core prescribing indicators and the most commonly prescribed medicines in a tertiary health care setting in a developing country. Advances in Pharmacological and Pharmaceutical Sciences. 2021;2021:6625377.
61. Merlob P, Stahl B, Kaplan B. Children born to mothers using multiple drug therapy during their pregnancy. International Journal of Risk and Safety in Medicine. 1996;8(3):237-41.
62. Eze UI, Eferakeya AE, Oparah AC, Enato EF. Assessment of prescription profile of pregnant women visiting antenatal clinics. Pharmacy Practice. 2007;5(3):135-9.
63. Belay M, Kahaliw W, Ergetie Z. Assessment of drug utilization pattern during pregnancy in adama riferral hospital, oromia region, ethiopia. International Journal of Pharmaceutical Sciences and Research. 2013;4(5):1905-11.
64. Van Gelder MMHJ, Vorstenbosch S, Te Winkel B, Van Puijenbroek EP, Roeleveld N. Using Web-Based Questionnaires to Assess Medication Use during Pregnancy: A Validation Study in 2 Prospectively Enrolled Cohorts. American Journal of Epidemiology. 2018;187(2):326-36.

For peer review only - http://bmjopen.bmj.com/site/about/guidelines.xhtml

65. Lynch MM, Amoozegar JB, McClure EM, Squiers LB, Broussard CS, Lind JN, et al. Improving Safe Use of Medications During Pregnancy: The Roles of Patients, Physicians, and Pharmacists. Qual Health Res. 2017;27(13):2071-80.
66. Daw JR, Hanley GE, Greyson DL, Morgan SG. Prescription drug use during pregnancy in developed countries: a systematic review. Pharmacoepidemiol Drug Saf. 2011;20(9):895-902.

For peer review only

For peer review only - http://bmjopen.bmj.com/site/about/guidelines.xhtml

For peer review only

For peer review only - http://bmjopen.bmj.com/site/about/guidelines.xhtml

Figure 1: **Preferred Reporting Items for Systematic Reviews and Meta-Analyses 2020 flow diagram**

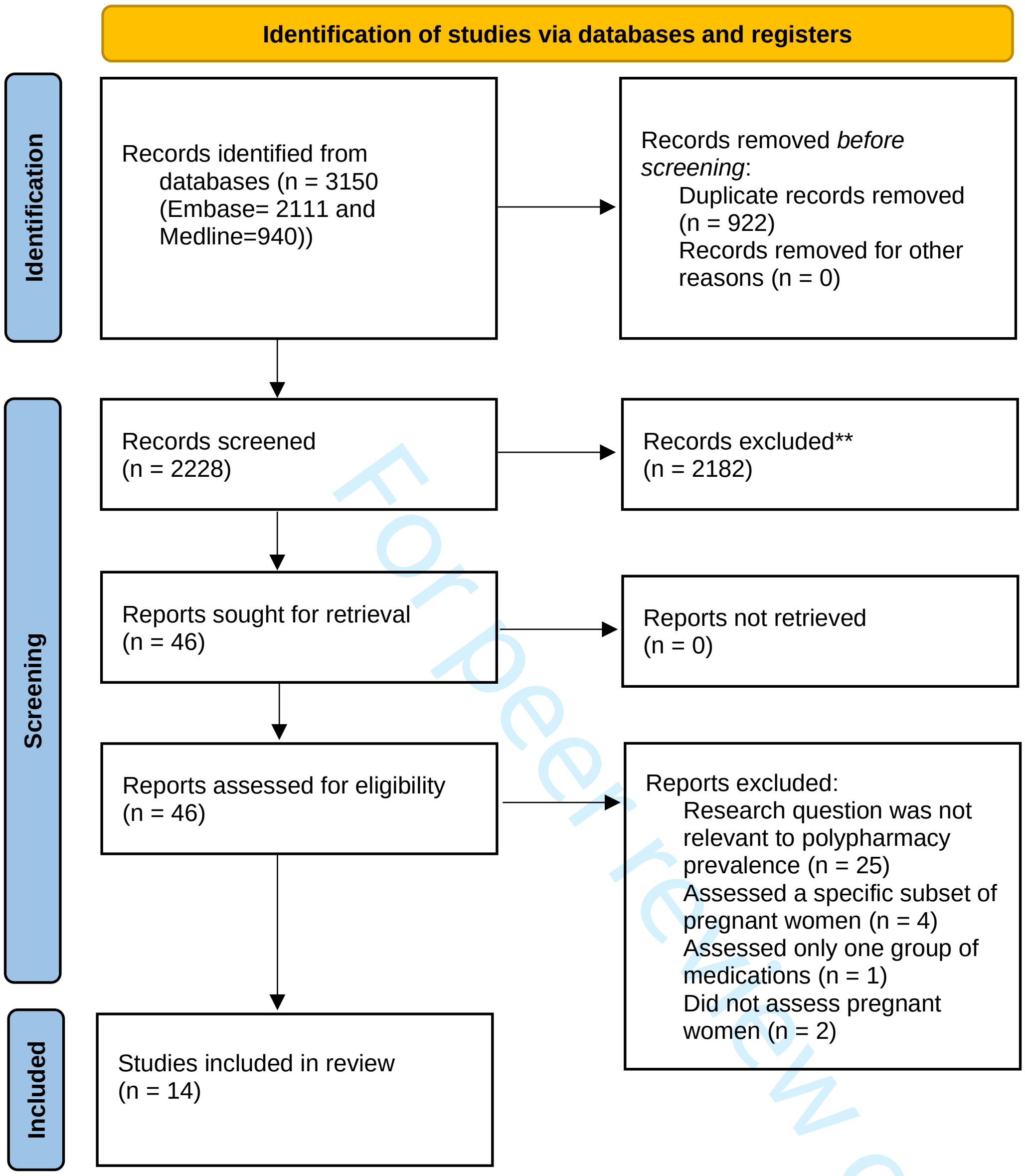

.

From: Page MJ, McKenzie JE, Bossuyt PM, Boutron I, Hoffmann TC, Mulrow CD, et al. The PRISMA 2020 statement: an updated guideline for reporting systematic reviews. BMJ 2021;372:n71. doi: 10.1136/bmj.n71

For more information, visit: http://www.prisma-statement.org/

For peer review only

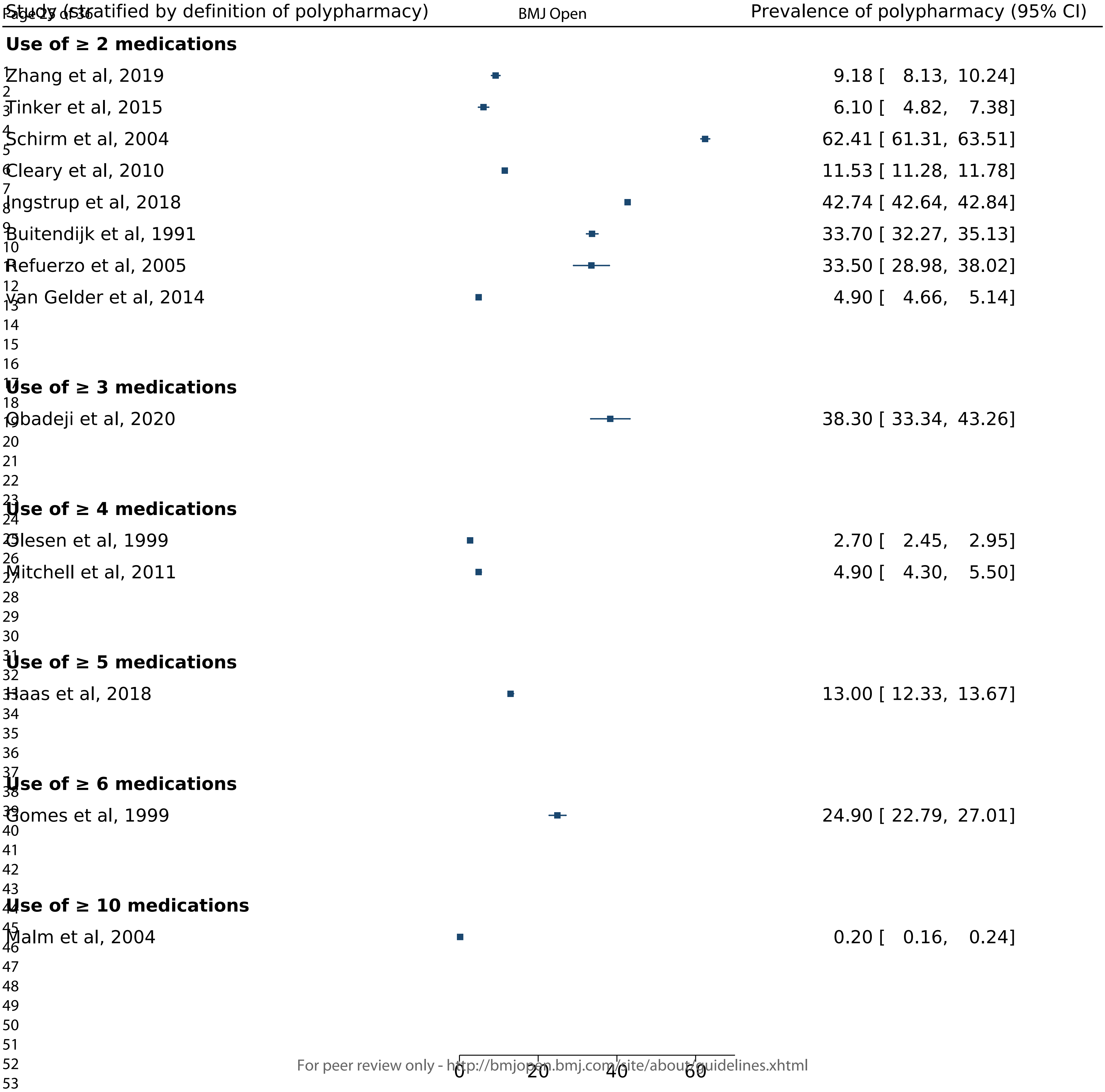

Figure 2: The Prevalence of Polypharmacy During Pregnancy Stratified by Definition of Polypharmacy

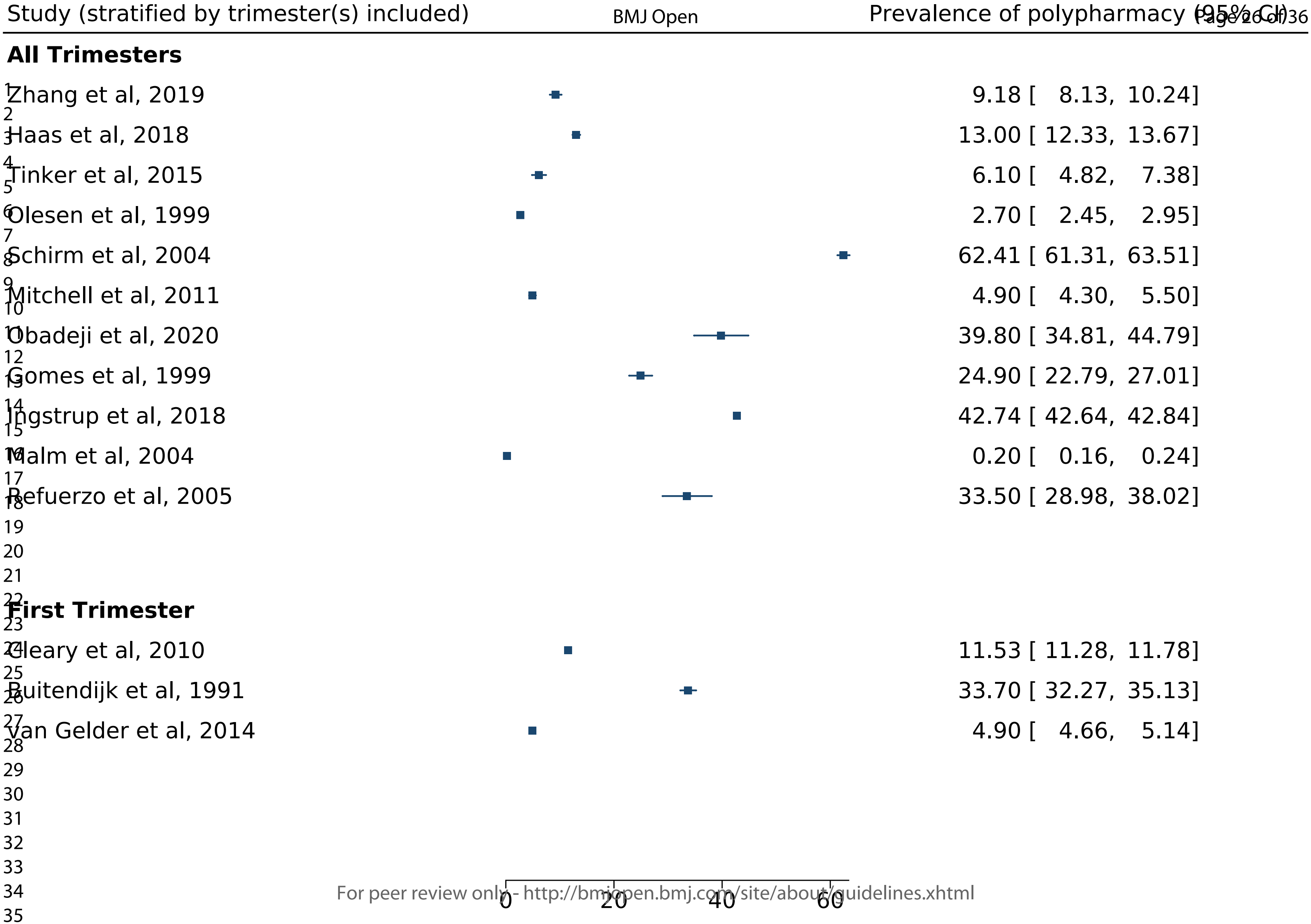

For peer review only - http://bmjopen.bmj.com/site/about/guidelines.xhtml

Figure 3: The Prevalence of Polypharmacy During Pregnancy Stratified by Period of Pregnancy

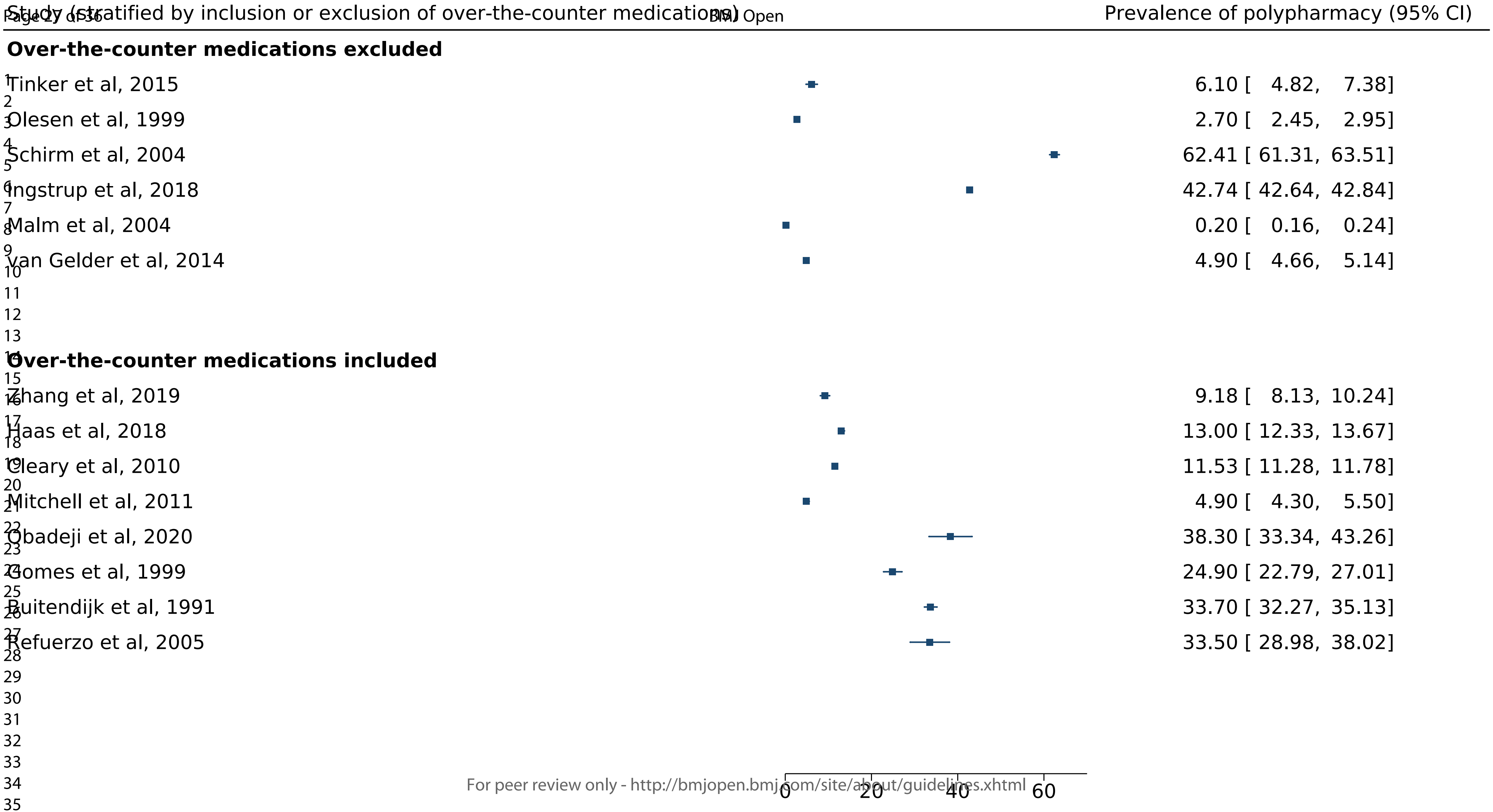

Figure 4: The Prevalence of Polypharmacy During Pregnancy Stratified by Inclusion or Exclusion of OTC Medications

Table S1- Summary of Newcastle-Ottawa Quality Assessment Scale Score for Included Studies

	Selection		Outcome		
**Author**	**Representativeness of the cohort**	**Ascertainment of pregnancy**	**Assessment of polypharmacy**	**Was follow-up long enough**	**Adequacy of follow-up**
**Buitendijk 1991 (29)**	*	*	-	*	*
**Olesen 1998 (31)**	*	*	*	*	*
**Gomes 1999 (22)**	*	*	-	*	*
**Malm 2004 (24)**	*	*	*	*	*
**Schirm 2004 (32)**	*	-	*	*	*
**Refuerzo 2005 (21)**	*	*	-	*	*
**Cleary 2010 (26)**	*	*	-	*	*
**Mitchell 2011 (27)**	*	*	-	*	*
**Van Gelder 2014 (20)**	*	-	*	*	*
**Tinker 2016 (23)**	-	-	-	*	*
**Haas 2018 (6)**	*	*	-	*	*
**Ingstrup 2018 (24)**	*	*	*	*	*
**Zhang 2019 (27)**	*	*	*	*	*
**Obadeji 2020 (29)**	*	*	*	*	*

* Indicates adequate quality in domain. A maximum of one star can be given for each domain

For peer review only

Appendix 1 – Search strategy

The search strategy for Embase and MEDLINE is shown below.

1. polypharmacy/
2. multiple medicatio*.mp.
3. multiple medicine*.mp.
4. multiple drug*.mp.
5. many medicatio*.mp.
6. many medicine*.mp.
7. many drug*.mp.
8. (more adj4 medication*).mp.
9. polydrug*.mp.
10. polymedication.mp.
11. polypharmacy.mp.
12. multi-drug therapy.mp.
13. multidrug therapy.mp.
14. multiple pharmacotherapy.mp.
15. poly pharmacy.mp.
16. polypragmasia.mp.
17. polypragmasy.mp.
18. exp pregnancy/
19. exp Pregnancy Complications/ or exp Pregnancy Disorders/
20. pregnan*.mp.
21. mothers/
22. perinatal.mp.
23. maternal.mp.
24. obstetric*.mp.
25. or/1-17
26. or/18-24
27. 25 and 26

For peer review only

Appendix S2 – Prisma Checklist

Section and Topic	Item #	Checklist item	Location where item is reported
**TITLE**			
Title	1	Identify the report as a systematic review.	Title, abstract, methods
**ABSTRACT**			
Abstract	2	See the PRISMA 2020 for Abstracts checklist.	See below
**INTRODUCTION**			
Rationale	3	Describe the rationale for the review in the context of existing knowledge.	Introduction
Objectives	4	Provide an explicit statement of the objective(s) or question(s) the review addresses.	Introduction
**METHODS**			
Eligibility criteria	5	Specify the inclusion and exclusion criteria for the review and how studies were grouped for the syntheses.	Methods – eligibility criteria
Information sources	6	Specify all databases, registers, websites, organisations, reference lists and other sources searched or consulted to identify studies. Specify the date when each source was last searched or consulted.	Methods – search strategy
Search strategy	7	Present the full search strategies for all databases, registers and websites, including any filters and limits used.	Methods – search strategy, Appendix S1
Selection process	8	Specify the methods used to decide whether a study met the inclusion criteria of the review, including how many reviewers screened each record and each report retrieved, whether they worked independently, and if applicable, details of automation tools used in the process.	Methods - study selection and data abstraction, author contribution
Data collection process	9	Specify the methods used to collect data from reports, including how many reviewers collected data from each report, whether they worked independently, any processes for obtaining or confirming data from study investigators, and if applicable, details of automation tools used in the process.	Methods - study selection and data abstraction, author contribution
Data items	10a	List and define all outcomes for which data were sought. Specify whether all results that were compatible with each outcome domain in each study were sought (e.g., for all measures, time points, analyses), and if not, the methods used to decide which results to collect.	Methods - study selection and data abstraction, outcome measurement
	10b	List and define all other variables for which data were sought (e.g., participant and intervention characteristics, funding sources). Describe any assumptions made about any missing or unclear information.	Methods - study selection and data

For peer review only

Section and Topic	Item #	Checklist item	Location where item is reported
			abstraction, exclusion criteria
Study risk of bias assessment	11	Specify the methods used to assess risk of bias in the included studies, including details of the tool(s) used, how many reviewers assessed each study and whether they worked independently, and if applicable, details of automation tools used in the process.	Methods - study selection and data abstraction
Effect measures	12	Specify for each outcome the effect measure(s) (e.g., risk ratio, mean difference) used in the synthesis or presentation of results.	Methods – outcome measurement
Synthesis methods	13a	Describe the processes used to decide which studies were eligible for each synthesis (e.g., tabulating the study intervention characteristics and comparing against the planned groups for each synthesis (item #5)).	Methods - study selection and data abstraction and summary measures and results synthesis
	13b	Describe any methods required to prepare the data for presentation or synthesis, such as handling of missing summary statistics, or data conversions.	Methods - summary measures and results synthesis
	13c	Describe any methods used to tabulate or visually display results of individual studies and syntheses.	Methods - summary measures and results synthesis
	13d	Describe any methods used to synthesize results and provide a rationale for the choice(s). If meta-analysis was performed, describe the model(s), method(s) to identify the presence and extent of statistical heterogeneity, and software package(s) used.	Methods - summary measures and results synthesis
	13e	Describe any methods used to explore possible causes of heterogeneity among study results (e.g., subgroup analysis, meta-regression).	N/a
	13f	Describe any sensitivity analyses conducted to assess robustness of the synthesized results.	N/a
Reporting bias assessment	14	Describe any methods used to assess risk of bias due to missing results in a synthesis (arising from reporting biases).	Methods - study selection and data abstraction (risk of bias)
Certainty assessment	15	Describe any methods used to assess certainty (or confidence) in the body of evidence for an outcome.	Methods - study selection and data abstraction (risk of bias)
**RESULTS**			
Study selection	16a	Describe the results of the search and selection process, from the number of records identified in the search to the number of studies included in the review, ideally using a flow diagram.	Figure 1, Results

For peer review only

Section and Topic	Item #	Checklist item	Location where item is reported
	16b	Cite studies that might appear to meet the inclusion criteria, but which were excluded, and explain why they were excluded.	Results, references
Study characteristics	17	Cite each included study and present its characteristics.	Results, Table s1
Risk of bias in studies	18	Present assessments of risk of bias for each included study.	Table S1, Results – risk of bias
Results of individual studies	19	For all outcomes, present, for each study: (a) summary statistics for each group (where appropriate) and (b) an effect estimate and its precision (e.g., confidence/credible interval), ideally using structured tables or plots.	Results, Figures 3-4
Results of syntheses	20a	For each synthesis, briefly summarise the characteristics and risk of bias among contributing studies.	Results
	20b	Present results of all statistical syntheses conducted. If meta-analysis was done, present for each the summary estimate and its precision (e.g. confidence/credible interval) and measures of statistical heterogeneity. If comparing groups, describe the direction of the effect.	N/a
	20c	Present results of all investigations of possible causes of heterogeneity among study results.	Results
	20d	Present results of all sensitivity analyses conducted to assess the robustness of the synthesized results.	Results
Reporting biases	21	Present assessments of risk of bias due to missing results (arising from reporting biases) for each synthesis assessed.	Results – risk of bias
Certainty of evidence	22	Present assessments of certainty (or confidence) in the body of evidence for each outcome assessed.	Results, Figures 3-4
**DISCUSSION**			
Discussion	23a	Provide a general interpretation of the results in the context of other evidence.	Discussion - interpretation
	23b	Discuss any limitations of the evidence included in the review.	Discussion – strengths and limitations
	23c	Discuss any limitations of the review processes used.	Discussion – strengths and limitations
	23d	Discuss implications of the results for practice, policy, and future research.	Conclusion
**OTHER INFORMATION**			
Registration and protocol	24a	Provide registration information for the review, including register name and registration number, or state that the review was not registered.	Methods – protocol and registration
	24b	Indicate where the review protocol can be accessed, or state that a protocol was not prepared.	Methods – protocol and registration and references

For peer review only

Section and Topic	Item #	Checklist item	Location where item is reported
	24c	Describe and explain any amendments to information provided at registration or in the protocol.	N/a
Support	25	Describe sources of financial or non-financial support for the review, and the role of the funders or sponsors in the review.	Funding
Competing interests	26	Declare any competing interests of review authors.	Disclosure of interest
Availability of data, code and other materials	27	Report which of the following are publicly available and where they can be found: template data collection forms; data extracted from included studies; data used for all analyses; analytic code; any other materials used in the review.	N/a

From: Page MJ, McKenzie JE, Bossuyt PM, Boutron I, Hoffmann TC, Mulrow CD, et al. The PRISMA 2020 statement: an updated guideline for reporting systematic reviews. BMJ 2021;372:n71. doi: 10.1136/bmj.n71

For more information, visit: http://www.prisma-statement.org/

For peer review only

Section and Topic	Item #	Checklist item	Reported (Yes/No)
**TITLE**			
Title	1	Identify the report as a systematic review.	Yes
**BACKGROUND**			
Objectives	2	Provide an explicit statement of the main objective(s) or question(s) the review addresses.	Yes
**METHODS**			
Eligibility criteria	3	Specify the inclusion and exclusion criteria for the review.	Yes
Information sources	4	Specify the information sources (e.g. databases, registers) used to identify studies and the date when each was last searched.	Yes
Risk of bias	5	Specify the methods used to assess risk of bias in the included studies.	Yes
Synthesis of results	6	Specify the methods used to present and synthesise results.	Yes
**RESULTS**			
Included studies	7	Give the total number of included studies and participants and summarise relevant characteristics of studies.	Yes
Synthesis of results	8	Present results for main outcomes, preferably indicating the number of included studies and participants for each. If meta-analysis was done, report the summary estimate and confidence/credible interval. If comparing groups, indicate the direction of the effect (i.e. which group is favoured).	Yes
**DISCUSSION**			
Limitations of evidence	9	Provide a brief summary of the limitations of the evidence included in the review (e.g. study risk of bias, inconsistency and imprecision).	Yes
Interpretation	10	Provide a general interpretation of the results and important implications.	Yes
**OTHER**			
Funding	11	Specify the primary source of funding for the review.	Yes
Registration	12	Provide the register name and registration number.	Prospero protocol cited in methods and references

From: Page MJ, McKenzie JE, Bossuyt PM, Boutron I, Hoffmann TC, Mulrow CD, et al. The PRISMA 2020 statement: an updated guideline for reporting systematic reviews. BMJ 2021;372:n71. doi: 10.1136/bmj.n71

For peer review only

PRISMA 2020 Checklist

Section and Topic	Item #	Checklist item	Location where item is reported
**TITLE**			
Title	1	Identify the report as a systematic review.	Title p1
**ABSTRACT**			
Abstract	2	See the PRISMA 2020 for Abstracts checklist.	Abstract p2
**INTRODUCTION**			
Rationale	3	Describe the rationale for the review in the context of existing knowledge.	Introduction p5
Objectives	4	Provide an explicit statement of the objective(s) or question(s) the review addresses.	Introduction p5
**METHODS**			
Eligibility criteria	5	Specify the inclusion and exclusion criteria for the review and how studies were grouped for the syntheses.	Methods p6
Information sources	6	Specify all databases, registers, websites, organisations, reference lists and other sources searched or consulted to identify studies. Specify the date when each source was last searched or consulted.	Methods p6
Search strategy	7	Present the full search strategies for all databases, registers and websites, including any filters and limits used.	Appendix S1
Selection process	8	Specify the methods used to decide whether a study met the inclusion criteria of the review, including how many reviewers screened each record and each report retrieved, whether they worked independently, and if applicable, details of automation tools used in the process.	Methods p7
Data collection process	9	Specify the methods used to collect data from reports, including how many reviewers collected data from each report, whether they worked independently, any processes for obtaining or confirming data from study investigators, and if applicable, details of automation tools used in the process.	Methods p7
Data items	10a	List and define all outcomes for which data were sought. Specify whether all results that were compatible with each outcome domain in each study were sought (e.g. for all measures, time points, analyses), and if not, the methods used to decide which results to collect.	Methods p7
	10b	List and define all other variables for which data were sought (e.g. participant and intervention characteristics, funding sources). Describe any assumptions made about any missing or unclear information.	Methods p7
Study risk of bias assessment	11	Specify the methods used to assess risk of bias in the included studies, including details of the tool(s) used, how many reviewers assessed each study and whether they worked independently, and if applicable, details of automation tools used in the process.	Methods p7
Effect measures	12	Specify for each outcome the effect measure(s) (e.g. risk ratio, mean difference) used in the synthesis or presentation of results.	Methods p7
Synthesis methods	13a	Describe the processes used to decide which studies were eligible for each synthesis (e.g. tabulating the study intervention characteristics and comparing against the planned groups for each synthesis (item #5)).	Methods p7
	13b	Describe any methods required to prepare the data for presentation or synthesis, such as handling of missing summary statistics, or data conversions.	Methods p7
	13c	Describe any methods used to tabulate or visually display results of individual studies and syntheses.	Methods p7
	13d	Describe any methods used to synthesize results and provide a rationale for the choice(s). If meta-analysis was performed, describe the model(s), method(s) to identify the presence and extent of statistical heterogeneity, and software package(s) used.	Methods p7
	13e	Describe any methods used to explore possible causes of heterogeneity among study results (e.g. subgroup analysis, meta-regression).	N/A
	13f	Describe any sensitivity analyses conducted to assess robustness of the synthesized results.	N/A
Reporting bias	14	Describe any methods used to assess risk of bias due to missing results in a synthesis (arising from reporting biases).	N/A

For peer review only

PRISMA 2020 Checklist

Section and Topic	Item #	Checklist item	Location where item is reported
assessment			narrative synthesis
Certainty assessment	15	Describe any methods used to assess certainty (or confidence) in the body of evidence for an outcome.	NA narrative synthesis
**RESULTS**			
Study selection	16a	Describe the results of the search and selection process, from the number of records identified in the search to the number of studies included in the review, ideally using a flow diagram.	Figure 1
	16b	Cite studies that might appear to meet the inclusion criteria, but which were excluded, and explain why they were excluded.	Results p8
Study characteristics	17	Cite each included study and present its characteristics.	Results p8 Table 1
Risk of bias in studies	18	Present assessments of risk of bias for each included study.	Results p8
Results of individual studies	19	For all outcomes, present, for each study: (a) summary statistics for each group (where appropriate) and (b) an effect estimate and its precision (e.g. confidence/credible interval), ideally using structured tables or plots.	Table 1
Results of syntheses	20a	For each synthesis, briefly summarise the characteristics and risk of bias among contributing studies.	N/A
	20b	Present results of all statistical syntheses conducted. If meta-analysis was done, present for each the summary estimate and its precision (e.g. confidence/credible interval) and measures of statistical heterogeneity. If comparing groups, describe the direction of the effect.	N/A
	20c	Present results of all investigations of possible causes of heterogeneity among study results.	N/A
	20d	Present results of all sensitivity analyses conducted to assess the robustness of the synthesized results.	N/A
Reporting biases	21	Present assessments of risk of bias due to missing results (arising from reporting biases) for each synthesis assessed.	N/A
Certainty of evidence	22	Present assessments of certainty (or confidence) in the body of evidence for each outcome assessed.	N/A
**DISCUSSION**			
Discussion	23a	Provide a general interpretation of the results in the context of other evidence.	Discussion p12
	23b	Discuss any limitations of the evidence included in the review.	Discussion p11
	23c	Discuss any limitations of the review processes used.	Discussion p11
	23d	Discuss implications of the results for practice, policy, and future research.	Discussion p12
**OTHER INFORMATION**			
Registration and protocol	24a	Provide registration information for the review, including register name and registration number, or state that the review was not registered.	Methods p6
	24b	Indicate where the review protocol can be accessed, or state that a protocol was not prepared.	Methods p6
	24c	Describe and explain any amendments to information provided at registration or in the protocol.	N/A

For peer review only

PRISMA 2020 Checklist

Section and Topic	Item #	Checklist item	Location where item is reported
Support	25	Describe sources of financial or non-financial support for the review, and the role of the funders or sponsors in the review.	Statements and declarations p13
Competing interests	26	Declare any competing interests of review authors.	Statements and declarations p13
Availability of data, code and other materials	27	Report which of the following are publicly available and where they can be found: template data collection forms; data extracted from included studies; data used for all analyses; analytic code; any other materials used in the review.	Supporting information

From: Page MJ, McKenzie JE, Bossuyt PM, Boutron I, Hoffmann TC, Mulrow CD, et al. The PRISMA 2020 statement: an updated guideline for reporting systematic reviews. BMJ 2021;372:n71. doi: 10.1136/bmj.n71

For more information, visit: http://www.prisma-statement.org/

For peer review only
