# Supplementary material for: Prevalence of polypharmacy in pregnancy: a systematic review
Source: BMJ Open. 2023 Mar 6;13(3):e067585. doi: 10.1136/bmjopen-2022-067585 (PMC9990613; doi:10.1136/bmjopen-2022-067585)
Supplement: Reviewer comments [file bmjopen-2022-067585.reviewer_comments.pdf]

PEER REVIEW HISTORY

BMJ Open publishes all reviews undertaken for accepted manuscripts. Reviewers are asked to complete a checklist review form (**http://bmjopen.bmj.com/site/about/resources/checklist.pdf**) and are provided with free text boxes to elaborate on their assessment. These free text comments are reproduced below.

ARTICLE DETAILS

**TITLE (PROVISIONAL)**	Prevalence of polypharmacy in pregnancy: a systematic review
**AUTHORS**	Anand, Astha; Phillips, Katherine; Subramanian, Anuradhaa; Lee, Siang Ing; Wang, Zhaonan; McCowan, Rebecca; Agrawal, Utkarsh; Fagbamigbe, Adeniyi; Nelson-Piercy, Catherine; Brocklehurst, Peter; Damase-Michel, Christine; Loane, Maria; Nirantharakumar, Krishnarajah; Azcoaga-Lorenzo, Amaya

VERSION 1 – REVIEW

**REVIEWER**	Czarniak, Petra<br>Curtin University, School of Pharmacy and Biomedical Sciences
**REVIEW RETURNED**	20-Sep-2022

**GENERAL COMMENTS**	Thank you for the opportunity to review the manuscript 'Prevalence of polypharmacy in pregnancy: a systematic review'. I found the article confusing in various sections and have a few questions and some comments for the author to consider. Please check the grammar and referencing, as there are several inconsistencies.<br><br>Abstract<br>• Line 25 Change 'intervention al' to interventional'<br><br>Introduction<br>• Line 7 Although the authors state that 'medications may be prescribed in pregnancy', please also consider stating that in addition to prescription drugs, over-the-counter (OTC) medications are used extensively. Further, the use of OTC medicines may increase over the course of the pregnancy.<br>• Line 13 Part of the sentence 'With rising medication use' is repetitive as it was stated in Line 10 ie '…has been rising over the past few decades'. Consider rewording.<br>• Line 18 Please provide references for the sentence 'Polypharmacy is broadly defined….'. How do other definitions differ to that provided by the authors?<br>• Line 23 Please consider adding further information about drug interactions<br>• Line 30 Please provide examples of how drug pharmacokinetics is altered in pregnancy<br>• Line 33 What do the authors mean by 'It is unclear what the effect of combining medications might be'. Are the authors referring to potential cytochrome P450 interactions, effects on p-glycoprotein or placental transporters? Please explain.<br><br>Methods<br>• Line 24 Please include the 'study authors' definition of polypharmacy'<br>• With respect to the eligibility criteria, were the authors interested in prescribed and OTC medicines, as well as herbal medicines, supplements, vitamins, illegal drugs, etc. Were there any exclusions?<br>• Line 21 As the current manuscript is a systematic review, why did the authors include systematic reviews in their eligibility criteria?

	• Line 59 Why did the authors decide to limit their search to two databases, rather than three? This may have limited the number of available articles.<br>• Why was the full search strategy for Medline not included as an appendix?<br>• In structuring the literature search for systematic reviews, a useful tool is PICO (Population, Intervention, Comparison, Outcome). Why did the authors not adopt the PICO approach?<br>• Please reference the Newcastle-Ottawa critical appraisal checklist.<br><br>Results<br>• How many records were identified in each database?<br>• Figure 1 (flow diagram) does not appear to be labelled<br>• Table 1 – consider listing the studies in a sequential order according to the year of publication.<br>• Table 1: In each of these studies, which drugs were most commonly taken/ prescribed during pregnancy?<br>• Table 1 – what was the most common medication combination in these studies (as the authors aimed to identify this)?<br>• Line 29-36 I find this confusing. The reader is referred to a study by Mitchell et al. (reference 64 and 65) but neither reference 64 or 65 are from Mitchell et al. A study by Mitchell et al. (reference 26) from 2011 is included in Table 1 but it is unclear how Mitchell et al. could have reported results from the BDS study (reference 64 – Line 29) when reference 64 was published in 2017. The paragraph is confusing, please revise.<br>• Risk of bias within studies – please assign reference numbers to references cited<br>• Figures are not labelled. Figures should appear in sequential order (Figure 4 is referred to before Figure 2)<br>• Figures are not labelled. Figures 2, 3 and 4 seem to be missing<br>• Table S1 requires clarification<br>• Please ensure to define abbreviations before they are used eg OTC<br>• In the methodology, the authors stated that a review of the literature was performed to identify the most common medication combinations. Although data on this has not been reported, it would be helpful to include a list of the most commonly prescribed or self-administered medications during pregnancy.<br><br>Discussion<br>• Consistency and accuracy in reporting data is recommended. For example, the prevalence of polypharmacy in the discussion is stated as ranging from 5 – 62% but in the abstract, it is stated as 4.9 – 61.3%.<br>• The authors state that they did not limit their search to studies published in English to minimise language bias. Who translated articles not published in English? How could the authors be confident that the translation was accurate?<br>• Please revise the sentence 'There are limited studies specifically assessing polypharmacy in pregnancy are limited'.<br>• The authors state that there is no consensus on the definition of polypharmacy. However, a number of published articles have defined polypharmacy as the concurrent use of several medications, with five or more drugs constituting the common definition (Bjerrum L, et al. Polypharmacy: correlations with sex, age and drug regimen A prescription database study. Eur J Clin Pharmacol. 1998;54(3):197-202; Page AT, et al. Polypharmacy among older Australians, 2006–2017: a population-based study. Med J Aust. 2019;211(2):71-75). The definition would be acceptable to various cohorts, including pregnant women.<br>• Line 55 Please include examples of outcomes of some common combinations of medications in pregnant women<br>• Interpretation: This paragraph is confusing as it states 5-62% of pregnant women take two or more medications. How is this related to a previous review which found 27-93% of women filled at least one prescription during pregnancy – one prescription is not polypharmacy.

**REVIEWER**	Anderson, Lorinda<br>Oregon State University, College of Pharmacy
**REVIEW RETURNED**	23-Sep-2022

**GENERAL COMMENTS**	Mostly great job of describing the results and the differences between the studies. Sometime was hard to follow so might be helpful to provide more headings for what is being described.<br><br>Introduction:<br>-Lines 32-33 mention that "even fewer studies assess the outcomes of polypharmacy", however in the results of this review, there were no studies that reported on associated pregnancy outcomes in women exposed to polypharmacy. Therefore, should this sentence be amended to state that there are no studies that directly mention outcomes related to polypharmacy?<br><br>Study Design:<br>-The authors mention that "systematic reviews" were included as a potential study that might be included in this "systematic review" but my understanding is that these are not the primary sources of data and not sure why these would be included as a study type.<br>-An "experienced librarian" was mentioned several times; I'm guessing this is meant to be similar to saying that a statistician was used. However, there is a wide range of what librarians are asked to do and are trained in, so would recommend including more information on what is meant by this. Is the librarian trained specifically on how to do thorough medline and embase searches?<br>-One of the criteria we are asked to look at is whether or not the references are "up to date" and not sure that going back to the beginning of the databased in 1946 and 1974 is current. Granted, it appears that the studies included only dated back to 1991, so not sure this is a big concern. Perhaps depends on when trends of increasing women using medications during pregnancy started to occur.<br><br>OTC and vitamin concerns:<br>-Would like to see the authors of this review give more of a description for what they consider as "medications" when including 2 or more as their definition. Did they report percentages based on the definition of "medications" used in each respective study?<br><br>-Would like to see a column in the summary chart on vitamins, and OTC's and whether the study included or excluded these in their definition of "medications". It was mentioned that some studies included this and others didn't, but would be nice to see this in the chart, and especially as it compares to the definition of polypharmacy given (even if the one used by the authors of this review).<br><br>Strengths and limitations:<br>-Please rework the first sentence starting on line 26. It has the word "limited" in it twice.<br>-The first sentence in the conclusion is never really flushed out in the previous discussion (or if it is, this is not straightforward). It reads that the variability in prevalence of polypharmacy with these studies is dependent on which medications were included. I do see that there is a section titled "prevalence of polypharmacy by medications included" in the text with the descriptions of the studies, however this doesn't appear to be elaborated on much, if at all, in the discussion and to especially make this the big conclusion. Please provide more on why you think the "which medications were included" is the most important variable in why there was such a wide range of results for "polypharmacy". I have a feeling this may also have to do with my previous questions on vitamins and OTC's but could be wrong.

VERSION 1 – AUTHOR RESPONSE

Comment 1

Line 25 Change 'intervention al' to interventional'

Response 1:

Thank you for pointing this out. We have made this change.

Introduction

Comment 2

Line 7 Although the authors state that 'medications may be prescribed in pregnancy', please also consider stating that in addition to prescription drugs, over-the-counter (OTC) medications are used extensively. Further, the use of OTC medicines may increase over the course of the pregnancy.

Response 2:

We agree that use of over-the-counter medication may increase in pregnancy and we discuss the impact of over the counter medication use and polypharmacy in our review. We have altered the wording here to reflect overall medication usage rather than prescribed medications only, as per reviewer advice:

Medications may be taken in pregnancy for the management of pregnancy-related symptoms (such as nausea and vomiting), pre-existing maternal health conditions or pregnancy-related complications.

Comment 3

Line 13 Part of the sentence 'With rising medication use' is repetitive as it was stated in Line 10 ie '…has been rising over the past few decades'. Consider rewording.

Response 3:

Repetitive phrasing has been reworded as advised:

The use of medications amongst pregnant women has been rising over the past few decades (4-6), which could be attributed to a rise in the prevalence of maternal comorbidities, obesity and a rise in the average maternal age(7, 8). With this, the use of multiple medications is also likely to increase (3).

Comment 4

Line 18 Please provide references for the sentence 'Polypharmacy is broadly defined….'. How do other definitions differ to that provided by the authors?

Response 4:

We have referenced a systematic review. In the description of this review in line 19-21 we have included the range of definitions of polypharmacy which Mansoor et al found ("ranging from the use of 2 or more medication to 11 or more medications").

Comment 5

Line 23 Please consider adding further information about drug interactions

Response 5:
We have added a line detailing why drug-drug interactions are important

It has been reported that, as the number of medications prescribed together increases, as does the number of potentially serious drug-drug interactions.

Comment 6

Line 30 Please provide examples of how drug pharmacokinetics is altered in pregnancy

We thank the reviewer for their comment. Accordingly, we have included the following examples of pharmacokinetic changes in pregnancy:

"For example, expanded plasma volume and maternal body fat in pregnancy increases the volume of distribution for hydrophilic and lipophilic drugs leading to lower plasma concentration. Moreover, increased hepatic and renal clearance during pregnancy can lead to subtherapeutic drug concentrations. "

Comment 7

Line 33 What do the authors mean by 'It is unclear what the effect of combining medications might be'. Are the authors referring to potential cytochrome P450 interactions, effects on p-glycoprotein or placental transporters? Please explain.

We acknowledge that this paragraph was unclear. This was intended to mean that, as few trials considering drug safety include pregnant women, it is unclear what the impact of combining medications is in pregnancy. We have removed this sentence. It now reads:

However, few clinical trials are undertaken amongst pregnant women due to concerns around maternal and fetal safety. It is, therefore, unknown whether polypharmacy during pregnancy will worsen known side effects, result in novel adverse events or indeed have a synergistic or beneficial effect.

Methods

Comment 8

Line 24 Please include the 'study authors' definition of polypharmacy'. With respect to the eligibility criteria, were the authors interested in prescribed and OTC medicines, as well as herbal medicines, supplements, vitamins, illegal drugs, etc. Were there any exclusions?

Response 8:

We thank the reviewer for the request for clarification.

The definition of polypharmacy was heterogenous and varied between each study. We retained the study authors' eligibility criteria for including over-the-counter medications or herbal remedies, supplement, vitamins. We have clarified this in the methods by amending.

The study authors' definition of polypharmacy was used and we retained the study authors' eligibility criteria for whether over-the-counter medications were included.

Comment 9

As the current manuscript is a systematic review, why did the authors include systematic reviews in their eligibility criteria?

Response 9:

Systematic reviews were included to review any appropriate studies through snowballing references and to systematically search for any similar existing studies.

Comment 10

Why did the authors decide to limit their search to two databases, rather than three? This may have limited the number of available articles.

Response 10:

We thank the reviewer for their comment. Using a combination of Embase (which includes over 8100 journals) and Medline (which includes over 5000 journals), we felt that there was adequate coverage of published articles in particular for studies addressing polypharmacy. Indeed Bramer et al. show that a combination of Medline/Embase has high coverage.

Bramer, W.M., Giustini, D. & Kramer, B.M.R. Comparing the coverage, recall, and precision of searches for 120 systematic reviews in Embase, MEDLINE, and Google Scholar: a prospective study. Syst Rev 5, 39 (2016). https://doi.org/10.1186/s13643-016-0215-7

Comment 11

Why was the full search strategy for Medline not included as an appendix?

We thank the reviewer for the comment. The search strategy for Embase and Medline were the same. We have now amended Appendix S1 to mention both databases.

Comment 12

In structuring the literature search for systematic reviews, a useful tool is PICO (Population, Intervention, Comparison, Outcome). Why did the authors not adopt the PICO approach?

Response 12

We thank the reviewer. As this is not a systematic review of intervention studies, there was no intervention or comparator. For the search strategy, we combined terms for pregnancy (the population) with polypharmacy (the exposure/ outcome).

Comment 13

Please reference the Newcastle-Ottawa critical appraisal checklist.

Response 13

We thank the reviewer for their input - we have referenced Newcastle-Ottawa critical appraisal checklist.

Results

Comment 14

How many records were identified in each database?

Response 14:

In Medline 940 records identified (prior to duplicates being removed) and in Embase 2111 records identified (prior to duplicates being removed). We have updated the PRISMA diagram to reflect this.

Comment 15

Figure 1 (flow diagram) does not appear to be labelled

Response 15:

Figure 1's label is "Figure 1: Preferred Reporting Items for Systematic Reviews and Meta-Analyses 2020 flow diagram". It appears to have been cut off when converted to pdf. Apologies, this has been amended.

Comment 16

Table 1 – consider listing the studies in a sequential order according to the year of publication.

Response 16:

Thank you for your comment. We have amended the table.

Comment 17

Table 1: In each of these studies, which drugs were most commonly taken/ prescribed during pregnancy?

Table 1 – what was the most common medication combination in these studies (as the authors aimed to identify this)?

Response 17:

We thank the reviewer for this insightful question. The question of common medications amongst pregnant women exposed to polypharmacy is of interest. Moreover, none of the studies commented on which medications were most commonly prescribed pairs amongst pregnant women exposed to polypharmacy.

However, some studies described the most commonly prescribed medications in their cohorts. We have added the following sentence to the Medications Used During Pregnancy section:

The most commonly prescribed or taken medications described in the studies were anti-emetics (6)(21)(25), antibiotics (6)(25-31), analgesia (6)(21)(25) and antacids (21)(28)(31) and vitamins or supplements (6)(27)(31).

Comment 18

Line 29-36 I find this confusing. The reader is referred to a study by Mitchell et al. (reference 64 and 65) but neither reference 64 or 65 are from Mitchell et al. A study by

Mitchell et al. (reference 26) from 2011 is included in Table 1 but it is unclear how Mitchell et al. could have reported results from the BDS study (reference 64 – Line 29) when reference 64 was published in 2017. The paragraph is confusing, please revise.

Response 18:

We thank the reviewer for highlighting the error in referencing. The correct reference is 26. This has been corrected. We have revised the paragraph to read:

Mitchell et al. reported results from two different cohorts; Birth Defect Study (BDS) and National Birth Defects Prevention Study (NBDPS)(26). Both studies contain data from mothers of babies born with birth defects and from a control group of mothers of babies born without birth defects. Mitchell et al reported data from both cases and controls in the BDS and from just the controls of the NBDPS. As pregnancies of mothers of babies born with birth defects are unlikely to be representative of the general population of pregnant women, only data from NBDPS were included in the results of this review.

Comment 19

Risk of bias within studies – please assign reference numbers to references cited

Response 19:

We thank the reviewer for their comment. The reference for Schirm and van Gelder have been moved from line 13 to line 9/10 for clarity.

Comment 20

Figures are not labelled. Figures should appear in sequential order (Figure 4 is referred to before Figure 2) Figures are not labelled. Figures 2, 3 and 4 seem to be missing

Response 20:
Thank you for pointing this out. We have labelled the figures and changed the order. The figures were uploaded separately to the manuscript. We have uploaded the again and they should be accessible.

Comment 21

Table S1 requires clarification

Response 21:

We thank the reviewer for their comment – Table S1 is a summary of the Newcastle-Ottawa Assessment Scale Score for included studies. We have updated the table heading to reflect this.

Comment 22

Please ensure to define abbreviations before they are used eg OTC

Response 22:

We thank the reviewer for their comment. OTC abbreviation is first used in page 9, line 21 where it is defined as over the counter. We have ensured that all abbreviations are defined the first time they are used.

Comment 23

In the methodology, the authors stated that a review of the literature was performed to identify the most common medication combinations. Although data on this has not been reported, it would be helpful to include a list of the most commonly prescribed or self-administered medications during pregnancy.

Response 23:

We thank the reviewer for their comment. None of the studies identified in this review specified which medications were used by pregnant women exposed to polypharmacy. This remains an important unanswered question.

As above, some studies reported the most commonly prescribed or administered medications during pregnancy. We have added the following sentence to the Medications Used During Pregnancy section:

The most commonly prescribed or taken medications described in the studies were anti-emetics (6)(21)(25), antibiotics (6)(25-31), analgesia (6)(21)(25) and antacids (21)(28)(31) and vitamins or supplements (6)(27)(31).

Discussion

Comment 24

Consistency and accuracy in reporting data is recommended. For example, the prevalence of polypharmacy in the discussion is stated as ranging from 5 – 62% but in the abstract, it is stated as 4.9 – 61.3%.

Response 24:

We thank the reviewer for highlighting the typographical error. In the discussion, the figures are rounded. In the abstract the figures read as ranging “from 4.9% (4.3%-5.5%) to 61.3% (61.3%-63.5%)”. This has been corrected so abstract now reads ranging “from 4.9% (4.3%-5.5%) to **62.4**% (61.3%-63.5%)”

Comment 25

The authors state that they did not limit their search to studies published in English to minimise language bias. Who translated articles not published in English? How could the authors be confident that the translation was accurate?

Response 25:

We thank the reviewer for their comment. Where possible, non-English papers were translated by a native speaker. We included one non English study (Gomes et al.). This paper was published in Portuguese. The entire paper was translated using an online translation tool (Google Translate) as per protocol. Two separate authors used this translation service independently and the article was read for sense check.

Comment 26

Please revise the sentence ‘There are limited studies specifically assessing polypharmacy in pregnancy are limited’.

Response 26:

We thank the reviewer for highlighting the grammar - this sentence has been corrected to read "There are limited studies specifically assessing polypharmacy in pregnancy".

Comment 27

The authors state that there is no consensus on the definition of polypharmacy. However, a number of published articles have defined polypharmacy as the concurrent use of several medications, with five or more drugs constituting the common definition (Bjerrum L, et al. Polypharmacy: correlations with sex, age and drug regimen A prescription database study. Eur J Clin Pharmacol. 1998;54(3):197-202; Page AT, et al. Polypharmacy among older Australians, 2006–2017: a population-based study. Med J Aust. 2019;211(2):71-75). The definition would be acceptable to various cohorts, including pregnant women.

Response 27:

We thank the reviewer for their comment. Indeed, the lack of consensus on the definition of polypharmacy can limit the interpretations of the studies assessing polypharmacy. We agree that five or more drugs constitutes one of the common definitions.

In a systematic review by Mansoor et al which aimed to identify studies defining polypharmacy, the most commonly reported definition of polypharmacy was use of five or more medications daily (n = 51, 46.4% of articles). However, the definition varied amongst the papers, with more than half of the papers defining polypharmacy in a different way and definitions of polypharmacy ranged from use of two or more medications daily to eleven or or more medications daily.

(Masnoon N, Shakib S, Kalisch-Ellett L, Caughey GE. What is polypharmacy? A systematic review of definitions. BMC Geriatr. 2017 Oct 10;17(1):230. doi: 10.1186/s12877-017-0621-2. PMID: 29017448; PMCID: PMC5635569.)

Comment 28

Line 55 Please include examples of outcomes of some common combinations of medications in pregnant women

Response 28:
We did not find any studies reporting examples of outcomes of associated with combinations of medications in pregnant women. Apologies that this line was misleading. We have changed this to say:

Association between rates of miscarriage and pre-term birth and medications used during pregnancy have been described in women with major psychiatric illnesses (12) however, none of the studies assessing polypharmacy in this systematic review evaluate the effect of taking multiple medication for the women and their offspring.

Comment 29:

Interpretation: This paragraph is confusing as it states 5-62% of pregnant women take two or more medications. How is this related to a previous review which found 27-93% of women filled at least one prescription during pregnancy – one prescription is not polypharmacy.

Response 29:

We thank the reviewer for their comments. We agree that that this wording is confusing. We have added a comment to show that both our study and previous review show high medication use during pregnancy.

Reviewer 2

Mostly great job of describing the results and the differences between the studies. Sometime was hard to follow so might be helpful to provide more headings for what is being described.

Response:

Thank you for this positive feedback. The following headings/ subheadings have been added to the results section:

- Prevalence of polypharmacy
    - Prevalence by polypharmacy definition
    - Prevalence of polypharmacy by trimester
    - Prevalence of polypharmacy by medications included
        - Over-the-counter medications
        - Exclusions of vitamins and minerals
- Medications used during pregnancy
- Multimorbidity and maternal or offspring outcomes

Introduction:

Comment 1

Lines 32-33 mention that "even fewer studies assess the outcomes of polypharmacy", however in the results of this review, there were no studies that reported on associated pregnancy outcomes in women exposed to polypharmacy. Therefore, should this sentence be amended to state that there are no studies that directly mention outcomes related to polypharmacy?

Response 1:

Thank you for your comment. We agree this sentence is incorrect and we have removed it rather than amending it, to avoid pre-empting the results of this review.

Study Design:

Comment 2

The authors mention that "systematic reviews" were included as a potential study that might be included in this "systematic review" but my understanding is that these are not the primary sources of data and not sure why these would be included as a study type.

Response 2:

We included systematic reviews to review any appropriate studies through snowballing references and to systematically search for any similar existing studies.

Comment 3

An "experienced librarian" was mentioned several times; I'm guessing this is meant to be similar to saying that a statistician was used. However, there is a wide range of what librarians are asked to do and are trained in, so would recommend including more information on what is meant by this. Is the librarian trained specifically on how to do thorough medline and embase searches?

Response 3:

We thank the reviewer for the clarification. Our librarian is a university librarian who is trained to run searches through databases such as medline and embase, with many year's experience based at University of St. Andrew's. As advised, we have included a brief description to clarify that our librarian was trained to undertake searches in large databases and removed the word "experienced".

Comment 4

One of the criteria we are asked to look at is whether or not the references are "up to date" and not sure that going back to the beginning of the databased in 1946 and 1974 is current. Granted, it appears that the studies included only dated back to 1991, so not sure this is a big concern. Perhaps depends on when trends of increasing women using medications during pregnancy started to occur.

Response 4:

We thank the reviewer for their comment. We agree that the pattern of medication use in pregnancy has changed over the decades. We still included the more historic searches for completion and to identify any previous studies with similar aims of assessing the burden of polypharmacy in pregnant women.

Comment 5

OTC and vitamin concerns:

-Would like to see the authors of this review give more of a description for what they consider as "medications" when including 2 or more as their definition. Did they report percentages based on the definition of "medications" used in each respective study?

We thank the reviewer for their comment. We carried forward the definition of "medication" used by the authors of the original study. The percentages are based on the definition of medication used in each respective study, as this data was available for each study.

Comment 6

-Would like to see a column in the summary chart on vitamins, and OTC's and whether the study included or excluded these in their definition of "medications". It was mentioned that some studies included this and others didn't, but would be nice to see this in the chart, and especially as it compares to the definition of polypharmacy given (even if the one used by the authors of this review).

We thank the reviewer for their suggestion. Figure 3 shows the prevalence of polypharmacy, subdivided by whether over-the-counter medications were included in the individual studies. We have incorporated the reviewer's suggestion and added a column to table 1 to reflect this data in a chart for ease of view.

Comment 7

Strengths and limitations:

Please rework the first sentence starting on line 26. It has the word "limited" in it twice.

Response 7:

Thank you for pointing this out - this has been corrected.

Comment 8

The first sentence in the conclusion is never really flushed out in the previous discussion (or if it is, this is not straightforward). It reads that the variability in prevalence of polypharmacy with these studies is dependent on which medications were included. I do see that there is a section titled "prevalence of polypharmacy by medications included" in the text with the descriptions of the studies, however this doesn't appear to be elaborated on much, if at all, in the discussion and to especially make this the big conclusion. Please provide more on why you think the "which medications were included" is the most important variable in why there was such a wide range of results for "polypharmacy". I have a feeling this may also have to do with my previous questions on vitamins and OTC's but could be wrong.

Response 8:

We agree the first sentence required expanding. We have changed this to read:

The reported prevalence of polypharmacy amongst pregnant women varies based on the number of medications considered in the definition, the trimester considered and the types of medications included.

VERSION 2 – REVIEW

**REVIEWER**	Anderson, Lorinda<br>Oregon State University, College of Pharmacy
**REVIEW RETURNED**	22-Nov-2022

**GENERAL COMMENTS**	Excellent job with the updates and edits. The paper is much easier to understand and follow, especially with the addition of more subtitles. I also appreciate the addition of more specific information in the table and figure on OTC's included with each study.
